# Epigenetic profiling linked to multisystem inflammatory syndrome in children (MIS-C): A multicenter, retrospective study

**DOI:** 10.1016/j.eclinm.2022.101515

**Published:** 2022-06-25

**Authors:** Veronica Davalos, Carlos A. García-Prieto, Gerardo Ferrer, Sergio Aguilera-Albesa, Juan Valencia-Ramos, Agustí Rodríguez-Palmero, Montserrat Ruiz, Laura Planas-Serra, Iolanda Jordan, Iosune Alegría, Patricia Flores-Pérez, Verónica Cantarín, Victoria Fumadó, Maria Teresa Viadero, Carlos Rodrigo, Maria Méndez-Hernández, Eduardo López-Granados, Roger Colobran, Jacques G. Rivière, Pere Soler-Palacín, Aurora Pujol, Manel Esteller

**Affiliations:** a Josep Carreras Leukaemia Research Institute (IJC), Badalona, Barcelona, Catalonia, Spain; b Life Sciences Department, Barcelona Supercomputing Center (BSC), Barcelona, Catalonia, Spain; c Centro de Investigación Biomédica en Red de Cancer (CIBERONC), Spain; d Navarra Health Service Hospital, Pamplona, Spain; e University Hospital of Burgos, Burgos, Spain; f Neurometabolic Diseases Laboratory, Bellvitge Biomedical Research Institute (IDIBELL), L'Hospitalet de Llobregat, Barcelona, Catalonia, Spain; g Centro de Investigación Biomédica en Red de Enfermedades Raras (CIBERER), Spain; h Germans Trias i Pujol Research Institute (IGTP), Universitat Autònoma de Barcelona (UAB), Badalona, Barcelona, Spain; i Pediatric Critical Care Unit, Hospital Universitari Sant Joan de Deu, Barcelona, Catalonia, Spain; j Pediatrics Department, Hospital Universitario Niño Jesús, Madrid, Spain; k Unitat de Malalties Infeccioses i Importades, Servei de Pediatría, Infectious and Imported Diseases, Pediatric Unit, Hospital Universitari Sant Joan de Deú, Barcelona, Catalonia, Spain; l Servicio de Pediatría del Hospital Universitario Marqués de Valdecilla, Santander, Spain; m Department of Immunology, La Paz University Hospital, Madrid, Spain; La Paz Institute of Biomedical Research, Madrid, Spain; n Immunology Division, Department of Clinical and Molecular Genetics, Hospital Universitari Vall d'Hebron (HUVH), Vall d'Hebron Research Institute (VHIR), Universitat Autònoma de Barcelona, Barcelona, Catalonia, Spain; o Pediatric Infectious Diseases and Immunodeficiencies Unit, Hospital Universitari Vall d'Hebron (HUVH), Vall d'Hebron Research Institute (VHIR), Universitat Autònoma de Barcelona, Barcelona, Catalonia, Spain; p Institució Catalana de Recerca i Estudis Avançats (ICREA), Barcelona, Catalonia, Spain; q Physiological Sciences Department, School of Medicine and Health Sciences, University of Barcelona (UB), Catalonia, Spain

**Keywords:** Multisystem inflammatory syndrome in children, COVID-19, Kawasaki disease, Epigenetics, DNA methylation

## Abstract

**Background:**

Most children and adolescents infected with the severe acute respiratory syndrome coronavirus 2 (SARS-CoV-2) remain asymptomatic or develop a mild coronavirus disease 2019 (COVID-19) that usually does not require medical intervention. However, a small proportion of pediatric patients develop a severe clinical condition, multisystem inflammatory syndrome in children (MIS-C). The involvement of epigenetics in the control of the immune response and viral activity prompted us to carry out an epigenomic study to uncover target loci regulated by DNA methylation that could be altered upon the appearance of MIS-C.

**Methods:**

Peripheral blood samples were recruited from 43 confirmed MIS-C patients. 69 non-COVID-19 pediatric samples and 15 COVID-19 pediatric samples without MIS-C were used as controls. The cases in the two groups were mixed and divided into discovery (MIS-C = 29 and non-MIS-C = 56) and validation (MIS-C = 14 and non-MIS-C = 28) cohorts, and balanced for age, gender and ethnic background. We interrogated 850,000 CpG sites of the human genome for DNA methylation variants.

**Findings:**

The DNA methylation content of 33 CpG loci was linked with the presence of MIS-C. Of these sites, 18 (54.5%) were located in described genes. The top candidate gene was the immune T-cell mediator ZEB2; and others highly ranked candidates included the regulator of natural killer cell functional competence SH2D1B; VWA8, which contains a domain of the Von Willebrand factor A involved in the pediatric hemostasis disease; and human leukocyte antigen complex member HLA-DRB1; in addition to pro-inflammatory genes such as CUL2 and AIM2. The identified loci were used to construct a DNA methylation profile (EPIMISC) that was associated with MIS-C in both cohorts. The EPIMISC signature was also overrepresented in Kawasaki disease patients, a childhood pathology with a possible viral trigger, that shares many of the clinical features of MIS-C.

**Interpretation:**

We have characterized DNA methylation loci that are associated with MIS-C diagnosis. The identified genes are likely contributors to the characteristic exaggerated host inflammatory response observed in these patients. The described epigenetic signature could also provide new targets for more specific therapies for the disorder.

**Funding:**

Unstoppable campaign of Josep Carreras Leukaemia Foundation, Fundació La Marató de TV3, Cellex Foundation and CERCA Programme/Generalitat de Catalunya.

**Research in context**

**Evidence before this study**

Most members of the pediatric population infected with the SARS-CoV-2 virus, which is responsible for the COVID-19 pandemic, escape severe disease. However, in a few cases, a rare and serious health condition, known as multisystem inflammatory syndrome in children (MIS-C), may occur. The clinical spectrum of MIS-C can affect multiple organ systems, often requiring admission to intensive care unit. Risk factors for the disease are not well defined, and the hyperinflammatory condition resembles another rare disorder known as Kawasaki disease. To our knowledge, this is the first epigenomic study of MIS-C after acute SARS-CoV-2 infection. Our search of PubMed on January 20th, 2022, limited to articles in English, but not by date, using the terms “MIS-C”, “epigenomics”, “DNA methylation”, and “marker”, identified no studies addressing this topic.

**Added value of this study**

Our results indicate the existence of distinct DNA methylation loci that distinguish MIS-C patients from COVID-19 pediatric patients without MIS-C, and from healthy children and adolescents without SARS-CoV-2 infection. The epigenetic sites found were mostly located within genes associated with immune response and pro-inflammatory pathways. Taking advantage of these DNA methylation markers, we produced an epigenomic profile that exhibited great accuracy in predicting MIS-C diagnosis. We have named this profile the EPIMISC.

**Implications of all the available evidence**

Our research has revealed new biomarkers linked to MIS-C onset that provide new information about the pathophysiological mechanisms of the disorder, and highlight its close similarity to Kawasaki disease. The genes identified could also be candidate targets for more precise treatments of the disease. Most importantly, the assessment of the DNA methylation levels of these loci can be swiftly added to the measurement of other biochemical and clinical parameters to improve early MIS-C diagnosis.

Alt-text: Unlabelled box

## Introduction

In late 2019, an unexpected increase in the number of pneumonia cases in China led to the identification of the severe acute respiratory syndrome coronavirus 2 (SARS-CoV-2),^1^ and the subsequent worldwide spread of the derived disease, termed COVID-19. At the time of the writing (January 24th, 2022), more than 350 million confirmed cases and more than 5,6 million deaths have been reported worldwide (https://coronavirus.jhu.edu/map.html). High mortality of COVID-19 patients with serious respiratory failure linked to acute respiratory distress syndrome (ARDS) and interstitial pneumonia have been associated with male sex, old age and concomitant medical conditions, such as diabetes, obesity, hypertension, and cardiovascular pathology.^2^ In comparison to the presentation in adults, most children and adolescents with SARS-CoV-2 infection are fully asymptomatic or have very mild clinical manifestations.^3^ The severity of the disease in the pediatric population also depends on their underlying conditions, and children may manifest ARDS and pneumonia as do adults.^3^ However, a more specific complication appeared in April 2020,^4^ a new and rare syndrome, termed multisystem inflammatory syndrome in children (MIS-C). This is also known as pediatric inflammatory multisystem syndrome temporally associated with SARS-CoV-2 infection (PIMS-TS). MIS-C arises days to weeks after the initial infection.^4^, ^5^, ^6^, ^7^, ^8^, ^9^, ^10^ Unlike severe adult COVID-19 patients, who are characterized by respiratory failure, MIS-C patients show a broad spectrum of additional clinical features (e.g., rash, fever, abdominal and/or chest pain, conjunctival hyperemia, etc.) as a result of multiple organ involvement (e.g., the cardiovascular, gastrointestinal, mucocutaneous, or hematological systems, amongst others). Although it is a rare disease, MIS-C is a serious health condition that require admission to intensive care unit in around 60% of cases, and ultimately lead to death to a not negligible 2% of cases.^5^ The exact pathways that give rise to the clinical manifestations of MIS-C, and the factors predisposing to development of the disease are largely unknown.^4^^−^^10^

In the context of adult COVID-19, in addition to the aforementioned concomitant medical conditions,^2^ genetic studies suggest that several genetic loci are associated with the severity of the disease (summarized in Supplementary Materials). We have also recently shown that epigenetic variation, particularly DNA methylation, which is altered in many human diseases,^11^ is also associated with adult COVID-19 severity.^12^ DNA and RNA viral activity are controlled by DNA methylation changes,^11^ but more importantly, this epigenetic mark is key to proper immune system activity and could predict the efficacy of immune-related therapies.^13^ To investigate if epigenetic changes are involved in MIS-C, we undertook a comprehensive epigenomic study to identify candidate DNA methylation loci linked to the disease that distinguish these patients from standard COVID-19 pediatric patients and from SARS-CoV-2-uninfected children and adolescent subjects of the pre-COVID-19 era.

## Methods

### Study design and participants

Whole blood samples and clinical data from 43 patients with MIS-C were previously collected between April 16th, 2020 and August 17th, 2021 from seven Hospitals in Spain. MIS-C was diagnosed based on the case definition provided by the World Health Organization. Briefly, children with clinical and biochemical evidence of inflammation in at least two systems, without other cause, and with evidence of SARS-CoV2 infection or close contact. The complete description is available in https://www.who.int/news-room/commentaries/detail/multisystem-inflammatory-syndrome-in-children-and-adolescents-with-covid-19. Clinicopathological characteristics of the MIS-C patients studied are summarized in Table 1. Whole blood samples were also obtained from 15 pediatric COVID-19 cases with no evidence of MIS-C, and from 69 healthy children and adolescents collected during the pre-COVID-19 era (before December 2019), in the setting of routine surgical procedures such as circumcision, orchiopexy, inguinal or umbilical hernia repair, adenoidectomy, tonsillectomy or tympanic membrane incision; or from unaffected sibling controls collected in previous studies. The data from these samples are summarized in Tables S1 and S2, respectively. The protocol of this retrospective study was approved by the ethics review boards of the participating institutions. Written informed consent was obtained from all participants. The study protocol is described in the Supplementary Methods.

**Table 1:** Clinicopathological characteristics of the studied MIS-C patients.

Characteristics	MIS-C cohorts		
	Discovery cohort (*N* = 29)	Validation cohort (*N* = 14)	Entire cohort (*N* = 43)
**Gender** - Frequency (%)			
Female	9 (31.0%)	8 (57.1%)	17 (39.5%)
Male	20 (69.0%)	6 (42.9%)	26 (60.5%)
**Age (years)** - Median [range]	8.0 [0.5–17]	6.5 [1–11]	7.0 [0.5–17]
**Age group**- Frequency (%)			
≤2 yr	5 (17.2%)	1 (7.1%)	6 (14.0%)
3–5 yr	4 (13.8%)	5 (35.7%)	9 (20.9%)
6–9 yr	7 (24.1%)	5 (35.7%)	12 (27.9%)
10–13 yr	8 (27.6%)	3 (21.4%)	11 (25.6%)
14–18 yr	5 (17.2%)	0 (0.0%)	5 (11.6%)
**Ethnicity** - Frequency (%)			
West-Eurasia	20 (69.0%)	11 (78.6%)	31 (72.1%)
Central-South America	6 (20.7%)	2 (14.3%)	8 (18.6%)
African	3 (10.3%)	1 (7.1%)	4 (9.3%)
**Underlying conditions** - Frequency (%)			
Previously healthy	26 (89.7%)	14 (100%)	40 (93.0%)
Asthma	3 (10.3%)	0 (0%)	3 (7.0%)
**SARS-CoV-2 status**- Frequency (%)			
IgG and/or PCR positive	26 (89.7%)	13 (92.9%)	39 (90.7%)
Near contact with SARS-CoV-2 positive*	3 (10.3%)	1 (7.1%)	4 (9.3%)
**Detection of additional virus** - Frequency (%)†	5 (17.2%)	0 (0.0%)	5 (11.6%)
**Organ system involvement** - Frequency (%)			
Two systems	4 (13.8%)	2 (14.3%)	6 (14.0%)
Three systems	5 (17.2%)	6 (42.9%)	11 (25.6%)
Four or more systems	20 (69.0%)	6 (42.9%)	26 (60.5%)
**Gastrointestinal involvement**# - Frequency (%)	26 (89.7%)	11 (78.6%)	37 (86.0%)
**Respiratory involvement**# - Frequency (%)	21 (72.4%)	6 (42.9%)	27 (62.8%)
**Cardiovascular involvement**# - Frequency (%)	22 (75.9%)	11 (78.6%)	33 (76.7%)
**Mucocutaneous involvement**# - Frequency (%)	19 (65.5%)	10 (71.4%)	29 (67.4%)
**Hematologic involvement**# - Frequency (%)	21 (72.4%)	8 (57.1%)	29 (67.4%)
**Neurologic involvement**# - Frequency (%)	6 (20.7%)	2 (14.3%)	8 (18.6%)
**Renal involvement**# - Frequency (%)	3 (10.3%)	0 (0.0%)	3 (7.0%)
**Musculoskeletal involvement**# - Frequency (%)	1 (3.4%)	1 (7.1%)	2 (4.7%)
**Highest level of care** - Frequency (%)			
Home	1 (3.4%)	0 (0.0%)	1 (2.3%)
Ward	8 (27.6%)	9 (64.3%)	17 (39.5%)
Intensive care unit	20 (69.0%)	5 (35.7%)	25 (58.1%)
**Oxygen supplementation** - Frequency (%)			
None	9 (31.0%)	10 (71.4%)	19 (44.2%)
Nasal cannula	7 (24.1%)	1 (7.1%)	8 (18.6%)
Non-Invasive Ventilation or High Flow Oxygen	10 (34.5%)	2 (14.3%)	12 (27.9%)
Mechanical Ventilation	2 (6.9%)	1 (7.1%)	3 (7.0%)
Extracorporeal membrane oxygenation	1 (3.4%)	0 (0%)	1 (2.3%)

⁎ For cases of unknown SARS-CoV-2 status.

† Additional viruses: Parainfluenza virus type 4 (HPIV-4), Rhinovirus/Enterovirus (HRV/ENT) and Adenovirus.

# Following the definitions used for organ involvement in Feldstein et al., *N Engl J Med*, 2020.

### DNA methylation data and computational analyses

The DNA methylation status of the studied samples was obtained using the Infinium MethylationEPIC Array (∼850,000 CpG sites) (Supplementary Methods). The MIS-C epigenetic signature, referred to hereafter as EPIMISC, was obtained by first identifying the probes differentially methylated between MIS-C cases and healthy control donors, filtering out in a second step those probes found to be differentially methylated between pediatric COVID-19 cases and healthy controls (Figure S1). This approach enabled us to effectively discover the differentially methylated probes between MIS-C and non-MIS-C cases. This involved deriving a linear model adjusted by the age covariate with the limma R package (v3.46.0), using the methylation values of the discovery dataset. A significance threshold for CpGs with a False Discovery Rate (FDR) adjusted *P-*value <0.05 and an absolute mean methylation beta difference between groups of >0.15 was established. The linear model was adjusted by the age covariate after performing a principal component analysis (PCA) that identified disease status and age as the greatest sources of variation in our dataset (Supplementary Methods). The significantly differential DNA methylation sites (Table S3) were used to train a supervised classification model based on a ridge-regularized logistic regression to predict MIS-C diagnosis using the glmnet R package (v4.1-1). The classification model was optimized by tuning parameters (best performance with alpha = 0 from ridge regression, and regularization parameter lambda = 0.1) after resampling with 10-fold cross-validation carried out three times using the caret package in R (v6.0-86). Once the model and tuning parameters values have been defined after resampling, our model performance was assessed using the receiver operating characteristic (ROC) and calibration curves. Further details are provided in Supplementary Methods.

### Role of funders

Funded by the Josep Carreras Leukaemia Foundation, the Cellex Foundation and the CERCA Programme of the Generalitat de Catalunya. Additional support was provided by the Fundació La Marató de TV3 (202131-32-33), MCIU/AEI/FEDER (RTI2018-094049-B-I00) and AGAUR (2017SGR1080). The sponsors of the study had no role in the study design, data collection, data analysis, data interpretation, or the writing of the manuscript. The authors collected the data, and had full access to all of the data in the study. They also took the final decision and had responsibility for submitting the study results for publication.

## Results

### Patients and epigenomic study

Between April 16th, 2020 and August 17th, 2021, we obtained whole blood samples from 43 patients diagnosed with MIS-C, using the case definition provided by the World Health Organization and summarized in Methods. Table 1 lists the clinicopathological features of the MIS-C patients studied. MIS-C-associated laboratory findings for these cases are summarized in Figure S2. Overall, the median age was 7.0 years old (Interquartile range, IQR = 7), and the majority of the children were male (26 cases; 60.5%) and from a West-Eurasian ethnic background (31 cases, 72.1%). Most patients exhibited a previous healthy status (40 cases, 93%) and IgG and/or PCR positivity for SARS-CoV-2 (39 cases, 90.7%). Most cases had affectation of four or more of their organ systems (26 cases, 60.5%), and were admitted to an intensive care unit (25 cases, 58.1%). As previously described in other MIS-C series, only a few cases presented prominent respiratory symptoms that required mechanical ventilation (3 cases, 7%), in contrast to the classic severe COVID-19 illness, which often requires active and interventional oxygen supplementation. We also collected whole blood samples from 15 pediatric COVID-19 patients with IgG-positive and/or PCR-positive status for SARS-CoV-2, but without MIS-C (Table S1). Finally, we obtained whole blood samples from 69 children collected before December 2019, when the COVID-19 disease first appeared (Table S2).

To optimize our analyses, we compared the MIS-C group (*n* = 43) with the non-MIS-C group (*n* = 84). The latter group comprised the pediatric COVID-19 cases without MIS-C (*n* = 15) and the pediatric controls obtained before the COVID-19 pandemic (*n* = 69). The 127 samples collected were divided into discovery and validation cohorts (85 and 42 cases, respectively) (Table 2). There were no significant differences between the two cohorts with respect to the frequencies of MIS-C and non-MIS-C cases (Fisher's exact test, *P* = 1)*,* gender (Fisher's exact test, *P* = 0.345), age (Mann–Whitney–Wilcoxon test, *P* = 0.282) and ethnicity (Fisher's exact test, *P* = 0.579) (Table 2). DNA from the whole blood samples was purified for all cases and analyzed to determine DNA methylation status. The study aimed to characterize those genomic sites with a distinct DNA methylation status in MIS-C patients compared with the non-MIS-C population. The overall study design is illustrated in Figure S1.

**Table 2:** Clinicopathological characteristics of the discovery and validation cohorts.

Characteristics	Cohorts		
	Discovery cohort	Validation cohort	Entire cohort
	(*N* = 85)	(*N* = 42)	(*N* = 127)
**Cases** - Frequency (%)			
MIS-C	29 (34.1%)	14 (33.3%)	43 (33.9%)
Non-MIS-C	56 (65.9%)	28 (66.7%)	84 (66.1%)
**Gender** - Frequency (%)			
Female	36 (42.4%)	22 (52.4%)	58 (45.7%)
Male	49 (57.6%)	20 (47.6%)	69 (54.3%)
**Age (years)** - Median [range]	9.0 [0–17]	7.5 [0–16]	8.0 [0 - 17]
**Age group**- Frequency (%)			
≤2 yr	11 (12.9%)	3 (7.1%)	14 (11.0%)
3–5 yr	11 (12.9%)	13 (31.0%)	24 (18.9%)
6–9 yr	22 (25.9%)	12 (28.6%)	34 (26.8%)
10–13 yr	23 (27.1%)	7 (16.7%)	30 (23.6%)
14–18 yr	18 (21.2%)	7 (16.7%)	25 (19.7%)
**Ethnicity** - Frequency (%)			
West-Eurasia	61 (71.8%)	25 (59.5%)	86 (67.7%)
Central-South America	7 (8.2%)	4 (9.5%)	11 (8.7%)
African	4 (4.7%)	2 (4.8%)	6 (4.7%)
Unknown	13 (15.3%)	11 (26.2%)	24 (18.9%)

### Epigenomic analysis of MIS-C in the discovery cohort

**Figure 1:**
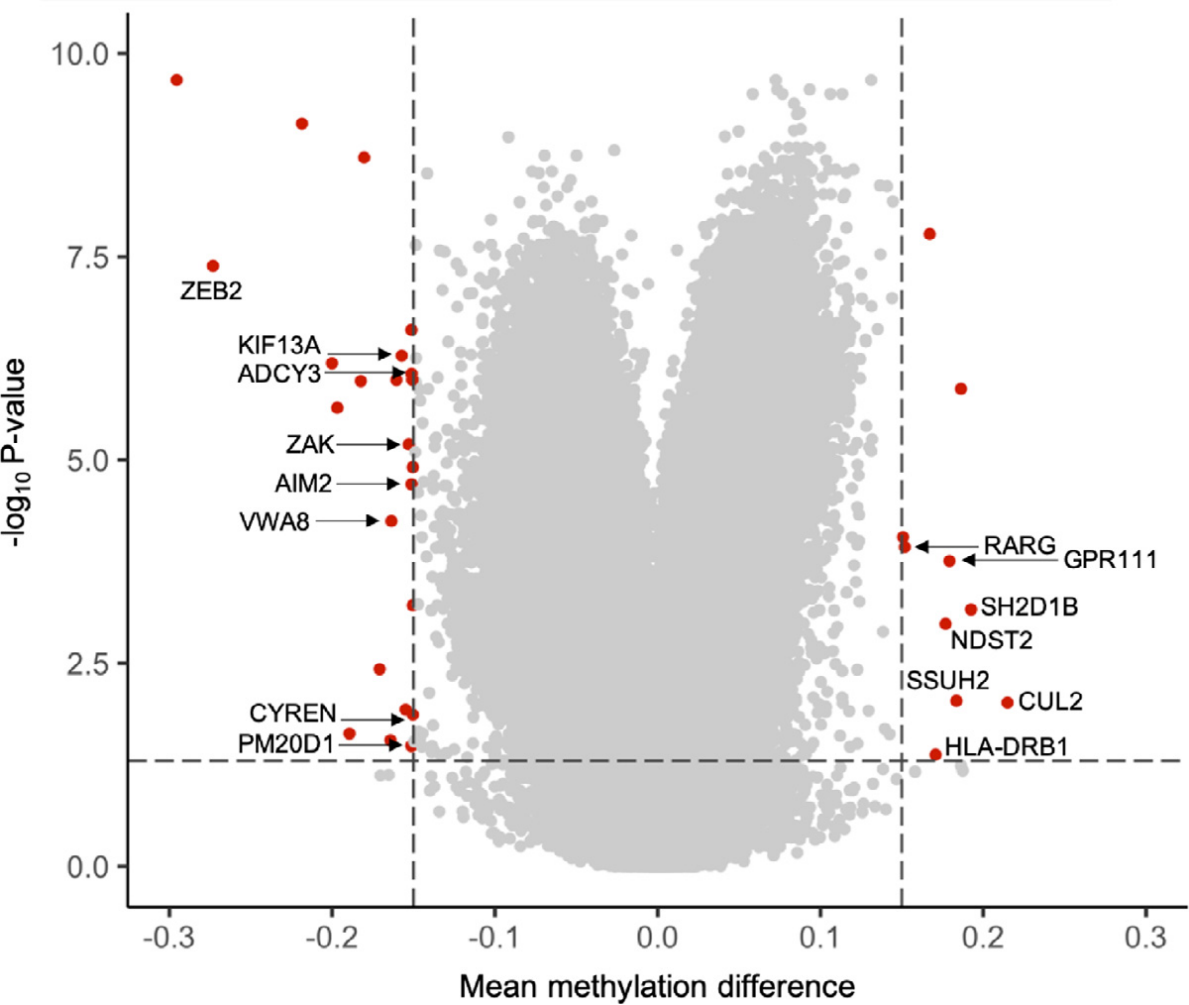
**The volcano plot shows significant differences in the DNA methylation status of 850K CpG sites between MIS-C and non-MISC using the described experimental and bioinformatic pipeline**. Y-axis shows the -log_10_*P*-value and X-axis shows the mean methylation difference according to beta value. A total of 33 CpGs with a delta beta >0.15 and FDR adjusted *P*-value <0.05 are shown in red. For those with an associated coding sequence, the gene name is also indicated. CpG-sites that exhibited a methylation beta value difference <0.15 and/or FDR adjusted *P*-value >0.05 are shown in grey. Dashed lines indicate cut-offs for significance.

Using the experimental and bioinformatic pipeline shown in Figure S1 and described in Supplementary Methods, the DNA methylation analysis of 85 pediatric individuals in the discovery cohort identified 33 CpG sites with a distinct methylation status between MIS-C (*n* = 29) and non-MIS-C (*n* = 56) cases (Table S3). The Volcano plot of the fully adjusted *P*-values from the DNA methylation loci linked to MIS-C diagnosis in the discovery cohort is shown in Figure 1. The genomic annotation of these differentially methylated 33 CpG sites is described in Table S3. Fifteen (45.45%) of the identified sites were located in regions of the genome with no currently annotated gene sequences; three (9.1%) were associated with three long non-coding RNAs (LINC00880, LOC645434, LOC100996286); and the other 15 (45.45%) CpG loci were located within 15 known protein-coding genes (Table 3).

**Table 3:** Epigenetic changes in coding genes associated with MIS-C diagnosis.

Gene symbol	Gene name	Gene function	Adjusted *P*-value
ADCY3	Adenylate cyclase 3	Catalyzes the synthesis of cyclic AMP (cAMP) from ATP. ADCY3 variants have been associated to risk/susceptibility to obesity, diabetes and chronic inflammatory diseases.	<0.001
AIM2	Absent in melanoma 2	Assembles the macromolecular inflammasome complex.	<0.001
CUL2	Cullin 2	Mediator of inflammation.	0.0096
CYREN	Cell cycle regulator of NHEJ	Cell-cycle-specific regulator of classical non-homologous end joining (NHEJ) of DNA double-strand break (DSB) repair.	0.0136
GPR111	G Protein-Coupled Receptor 111	Member of the adhesion G protein-coupled receptors (aGPCRs).	<0.001
HLA-DRB1	Major histocompatibility complex, class II, DR beta 1	Encodes a beta chain of antigen-presenting major histocompatibility complex class II (MHCII) molecule.	0.0421
KIF13A	Kinesin family member 13A	Motor protein that also mediates the trafficking of influenza A virus ribonucleoproteins, and transport of an arenavirus protein.	<0.001
NDST2	N-deacetylase and N-sulfotransferase 2	Enzyme with dual functions in processing glucosamine and heparin polymers.	0.0010
PM20D1	Peptidase M20 domain containing 1	Enzyme that regulates the production of N-fatty-acyl amino acids. Considered a metabolic disease-associated gene also linked to neurodegenerative disorders.	0.0328
RARG	Retinoic acid receptor gamma	Receptor for retinoic acid. Act as transcriptional regulator.	<0.001
SH2D1B	SH2 domain containing 1B	Adaptor protein for the signaling lymphocytic activation molecule family of receptors that enhances immune responses to antigens, including viral proteins such as HIV-Gag.	<0.001
SSUH2	Ssu-2 homolog	A putative chaperone protein.	0.0092
VWA8	Von Willebrand factor A domain-containing protein 8	Mitochondrial ATPase protein.	<0.001
ZAK	ZAK1 Homolog, Leucine Zipper And Sterile-Alpha Motif Kinase	Mitogen-activated protein kinase, also known as MAP3K20.	<0.001
ZEB2	Zinc finger E-box binding homeobox 2	ZEB2 is a DNA-binding transcriptional repressor.	<0.001

To investigate further the activities of the 15 candidate coding genes identified by the MIS-C DNA methylation screening, we performed an enrichment analysis (Supplementary Methods). Significantly enriched Gene Ontology (GO) biological processes (hypergeometric test, FDR adjusted *P-*value < 0.05) included “regulation of inflammatory response to antigenic stimulus” and “regulation of immune response”. All these enriched processes and pathways indicate that a broad exaggerated engagement of the immune response to the SARS-CoV-2 infection contributes to the characteristic hyperinflammatory clinical picture observed in these children.

Of the 15 candidate coding genes derived from the MIS-C epigenomic analysis, among the highest ranked coding genes according to the DNA methylation difference and adjusted *P*-value derived from the MIS-C epigenomic analysis (Table S3), the zinc finger E-box binding homeobox 2 (ZEB2) gene, the G protein-coupled receptor 111 (GPR111) gene, the SH2 domain containing 1B (SH2D1B) gene and the ubiquitin-protein ligase component Cullin-2 (CUL2) exhibit activities that could directly relate to MIS-C (Table 3). ZEB2 promotes terminal differentiation of effector and memory T cell populations during infections and the development of plasmacytoid dendritic cells, monocytes, B-cells, natural killer cells, and macrophages.^14^ GPR111 is involved in tolerance induction, granulopoiesis and the control of cytotoxicity.^15^ SH2D1B is a unique adaptor protein that enhances innate and adaptive immune responses to antigens.^16^^,^^17^ In this regard, the SH2D1B signaling pathway has the potential to be co-opted to produce enhanced vaccination responses.^16^ CUL2 is a mediator of inflammation and, in this regard, its pharmacological inhibition protects against hyperinflammatory responses,^18^ a finding that could be relevant for those MIS-C patients that do not respond to the standard treatment.

Of the other genes with a distinct DNA methylation profile in MIS-C patients (Table 3), the cases of AIM2 (absent in melanoma 2) and PM20D1 (peptidase M20 domain-containing 1) are particularly interesting because methylation events at these loci are also characteristic of adults who develop severe COVID-19 disease.^12^ The AIM2 gene is related to the hyperinflammatory manifestation of MIS-C patients since triggers caspase-1 and unleashes pro-inflammatory cytokines such as IL-1β and IL-18,^19^ which are also involved in the innate immune response to viral infections. Regarding PM20D1, recent data suggest that it contributes to autoimmune disorders and allergies,^20^^,^^21^ all of which are pathologies with an important hyperinflammatory component. In this study, we have identified that a DNA methylation site of the HLA-DRB1 (major histocompatibility complex, class II, DR beta 1) gene is linked to MIS-C. Interestingly, our study of adult COVID-19 cases identified that an epigenetic mark in HLA-C (major histocompatibility complex, class I, C) was associated with the severe disease.^12^ Importantly, allelic genotypes of HLA-DRB1 have been associated with the clinical severity of adult COVID-19 cases,^22^, ^23^, ^24^ and CD8^+^ T-cells from critically ill adult COVID-19 patients show upregulation of the HLA-DRB1 gene.^25^

We also investigated whether the DNA methylation status of MIS-C was distinct from that of non-MIS-C groups for genes that, according to the literature, are likely candidates for adult COVID-19. The 47 genes analyzed were the ACE2 receptor and TMPRSS2 protease, GWAS-derived genes, genes associated with inborn errors of type I IFN immunity in cases with life-threatening COVID-19, and other genes involved in immune host-cell pathways (Table S4). Only one gene, VWA8 (Von Willebrand factor A domain-containing protein 8), was shared in the list of COVID-19 associated loci (Table S4) and in our MIS-C associated DNA methylation sites (Table 3). A single nucleotide polymorphism in VWA8 has been linked to hospitalized cases in COVID-19 cases.^26^ For MIS-C genetic susceptibility very little is known. Three genes with reported sequence variants for MIS-C (SOCS1, XIAP and CYBB)^27^ were not differentially methylated in our cohorts (Table S5), in line with the idea that, for the same candidate target, genetic and epigenetic alteration are usually mutually exclusive*.*

### Testing MIS-C-associated DNA methylation markers in the validation cohort, and development of the EPIMISC signature

The DNA methylation status of single CpG sites linked to the presence of MIS-C in the discovery cohort (*n* = 85) was confirmed in the validation cohort (*n* = 42). Overall, when we individually analyzed the 33 CpGs whose DNA methylation levels differed significantly between the MIS-C and non-MIS-C cases, 20 (60.6%) were also significantly associated with the severe pediatric disorder in the validation cohort (Table S6). Of these 20 CpG sites, seven loci were located in the aforementioned gene coding-containing sequences (35%). Importantly, when we interrogated all the samples as an entire set, comprising the discovery and validation cohorts (*n* = 127), 24 of 33 (72.7%) individual CpG sites remained associated with MIS-C development (Table S6).

**Figure 2:**
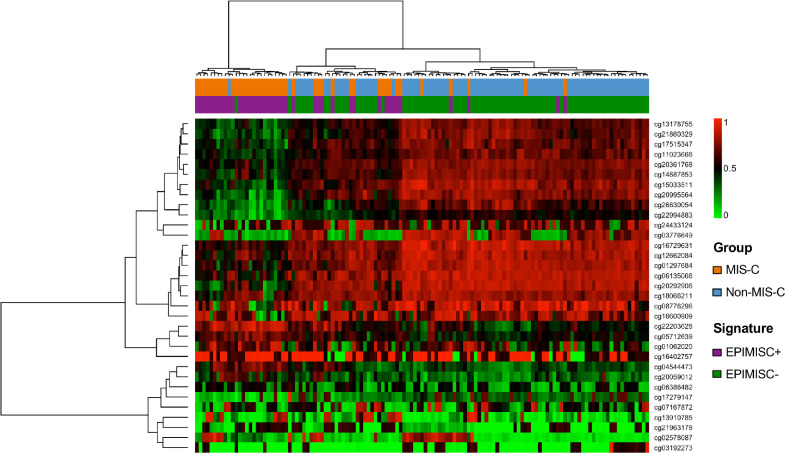
Heatmap representing the entire cohort of MIS-C and non-MIS-C cases clustered by methylation beta values of the 33 CpGs defining the EPIMISC signature. Cluster analysis was performed using the Ward.D clustering method and assuming Manhattan distances.

The discovery of single DNA methylation sites linked to the presence of MIS-C could be very helpful, but the establishment of an overall epigenomic signature could also be of great value to our understanding of the pathophysiological basis of the diseases and its clinical management. To achieve this, we selected the 33 significantly differential DNA methylation sites that were associated with the occurrence of MIS-C (Table S3) to train our discovery set, using a supervised classification model based on ridge-regularized logistic regression (see Supplementary Methods). By this method, we obtained a DNA methylation signature, hereafter referred to as EPIMISC, that was associated with MIS-C diagnosis (EPIMISC positive). It had a specificity of 98.21% (95% confidence interval CI = 90.45% to 99.95%), a sensitivity of 93.10% (95% CI = 77.23% to 99.15%), and positive and negative predictive values (PPV and NPV) of 96.43% (95% CI = 81.65% to 99.91%) and 96.49% (95% CI = 87.89% to 99.57%), respectively. Its accuracy was 96.47% (95% CI = 90.03% to 99.27%) and the Kappa value was 0.9208 (95% CI = 0.8329 to 1). We also plotted the Receiver Operating Characteristic (ROC) curve and calculated the Area Under the Curve [AUC = 95.66% (95% CI = 90.65% to 100%)] together with the calibration curve to further assess and visualize the model's performance (Figure S3). Supervised hierarchical clustering using the EPIMISC signature differentiated two branches that were significantly enriched with respect to each condition, MIS-C *vs.* non-MIS-C (Fisher's exact test, *P* = 4.3e-09) (Figure S4). Most important, we found that the EPIMISC signature kept its value in our validation cohort, being associated with the disease with a specificity of 92.86% (95% CI = 76.50% to 99.12%), a sensitivity of 85.71% (95% CI = 57.19% to 98.22%), and PPV and NPV of 85.71% (95% CI = 57.19% to 98.22%) and 92.86% (95% CI = 76.50% to 99.12%), respectively. The accuracy was 90.48% (95% CI = 77.38% to 97.34%) and the Kappa value was 0.7857 (95% CI = 0.5865 to 0.9849). The ROC curve and AUC [89.29% (95% CI = 78.61% to 99.97%)] alongside the calibration curve were also determined (Figure S5). The EPIMISC signature in the validation cohort also distinguished two branches with respect to MIS-C and non-MIS-C samples (Fisher's exact test, *P* = 3.1e-06) (Figure S6). Finally, for the entire cohort, EPIMISC was associated with MIS-C diagnosis with a specificity of 96.43% (95% CI = 89.92% to 99.26%), a sensitivity of 90.70% (95% CI = 77.86% to 97.41%), and PPV and NPV of 92.86% (95% CI = 80.52% to 98.50%) and 95.29% (95% CI = 88.39% to 98.70%), respectively. Its accuracy was 94.49% (95% CI = 88.97% to 97.76%) and the Kappa value was 0.8762 (95% CI = 0.7872 to 0.9653). The ROC curve and AUC (93.56% [95% CI = 88.74% to 98.39%)] alongside the calibration curve were also determined (Figure S7). The application of the EPIMISC signature for the entire cohort also classified samples as MIS-C or non-MIS-C (Fisher's exact test, *P* = 6.5e-14) (Figure 2). The five cases with concomitant viral infections (Table 1) were all of them EPIMISC positive, whereas the epigenomic signature was observed in 2 of 4 (50%) cases classified clinically as MIS-C but without any biological probe of SARS-CoV-2 infection (Table 1).

To further assess the specificity of the EPIMISC signature for the disease, we run our classification model to establish whether it was also overrepresented in available public DNA methylation datasets (GEO data repository) for other distinct pediatric inflammatory disorders. We found that the EPIMISC signature was not present in juvenile localized scleroderma (GEO GSE175379), juvenile systemic sclerosis (GEO GSE175379), or atopic dermatitis (GEO GSE152084). Similarly, the EPIMISC signature was almost non-existent in the general population (0.4%, 1 of 241 donors) (GEO GSE142512; GEO GSE132181). These samples were collected before the emergence of COVID-19, so the donors had never been exposed to the SARS-CoV-2 virus. Our observation that two of the targeted methylated genes within EPIMISC were shared with severe adult COVID-19 cases (AIM2 and PM20D1) prompted us to investigate whether the EPIMISC signature was also present in non-pediatric COVID-19 cases.^12^ We found that although EPIMISC was almost completely absent from asymptomatic and mild adult COVID-19 patients (1%, 2 of 194), it was present in 24.9% (53 of 213) of adult COVID-19 patients with clinical severity. MIS-C and critically-ill COVID-19 patients show some distinct clinicopathological characteristics, but also some commonalities. This last observation can relate to the targeting of similar cellular networks. For example, the activation of the inflammasome pathobiological pathway represented by the AIM2 gene occurs in COVID-19 adult patients^28^ and it was also associated with the severity of the disease in adult cases^12^ and, at the same time, the AIM2 gene is a key component of the EPIMISC signature identified herein. But targeting of distinct pathobiological pathways between both disorders also occur. For example the genes in our EPIMISC signature showed a significant enriched Gene Ontology (GO) biological processes (hypergeometric test, adjusted *P* < 0.05) of “regulation of natural killer cell mediated immunity (GO:0002715)” and “peptide antigen assembly with MHC protein complex (GO:0002501)” that were not observed in critically-ill COVID-19 adults.^12^ Thus, epigenetic and clinical commonalities and singularities between both disorders occur. Finally, we also wondered if the identified epigenomic profile was overrepresented in diseases involving other viral infections. We observed that the EPIMISC signature was present only in 7.8% (5 out 64) of patients with other viral respiratory infections (GSE167202; Ref.^29^). The interrogated GSE167202 cohort included rhinovirus/enterovirus (33%), influenza A (17%), metapneumovirus (13%), influenza B (11%), other coronavirus (11%), respiratory syncytial virus (9%), parainfluenza (5%), and adenovirus (2%) cases. Most important, the EPIMISC signature was absent in all HIV cases (*n* = 70) analyzed in a recently published cohort (GSE140800; Ref.^30^). Thus, overall, these results further support the specificity of the EPIMISC signature.

MIS-C is considered a new pediatric inflammatory entity associated with SARS-CoV-2 infection, but there is clinical overlap with another disorder, Kawasaki disease,^31^^−^^34^ a childhood febrile and systemic vasculitis thought to be triggered by exposure to a novel ribonucleic acid, as occurs in viral infections. This is a similar scenario to that presented by SARS-CoV-2 and MIS-C. Remarkably, when we run our classification model to assess the presence of the EPIMISC signature in DNA methylation profiles of Kawasaki disease patients available in the GEO database (GEO GSE84624),^35^ we found the EPIMISC signature in 95.8% (23 of 24) of the cases. There are other similarities between the two disorders. For example, beta-catenin contributed to the pathogenesis of Kawasaki disease,^35^ and a highly ranked gene of our EPIMISC signature was ZEB2. This gene is involved in the epithelial-mesenchymal transition (EMT), as it also occurs with the Wnt/beta-catenin signaling, but it is also essential for regulating hematopoiesis.^36^ Another example of features common to the two clinical entities was the suggested activation of neutrophils in Kawasaki disease.^37^ Using a deconvolution approach to calculate hematological cell populations (Supplementary Methods), we found that our MIS-C cases were also enriched in neutrophils relative to the non-MIS-C cases (Mann–Whitney–Wilcoxon test, *P* = 1.7e-05). Finally, although the EPIMISC signature was overrepresented in the Kawasaki disease, four CpGs were distinctly methylated between both disorders. One site was not associated with any known gene (cg16729631), and another was located in the GPR111 gene, which was a highly ranked candidate for the MIS-C cases. The other two sites were located in SSUH2 (ssu-2 homolog), a protein chaperone,^38^ and RARG (retinoic acid receptor gamma) which is associated with rubella virus-induced cytokine immune responses.^39^ These data are germane to similar findings showing that MIS-C and Kawasaki disease share many inflammatory biomarkers, but others are unique such as the high concentration of IFN-gamma-induced CXCL9 in MIS-C cases.^34^ The DNA methylation analysis of 33 reported GWAS-derived candidate genes for Kawasaki disease did not show any significant CpG methylation difference in our cohorts (Table S7). Overall, our results highlight the close epigenetic resemblance of MIS-C and Kawasaki disease, further suggesting that a viral infection could unleash the plethora of clinical manifestations that they share.

## Discussion

To the best of our knowledge, this is the first study to establish the epigenomic profile of MIS-C patients upon diagnosis. Gene Ontology analyses showed enrichment of the differentially methylated sites in genes associated with an immune response triggered by the SARS-CoV-2 infection. This immune overreaction may well explain the hyperinflammatory phenotype manifested in these children and why multiple body organs and tissues are affected. ZEB2, GPR111, SH2D1B, and HLA-DRB1 are examples of targeted genes, all of which are involved in the generation of immune and inflammatory responses to virus. Interestingly, the EPIMISC signature that we found to be associated with the presence of MIS-C in the discovery and validation cohorts was not linked to other pediatric inflammatory disorders that occur without involvement of a viral agent.

Some of the identified MIS-C epigenetic targets, such as AIM2 and PM20D1, and the EPIMISC signature overall, are also present in some severe adult COVID-19 cases, confirming that both processes (MIS-C in pediatrics and severe acute respiratory distress syndrome in adults) are inflammatory post-infectious complications and probably could be differently treated than the initial phase of the viral infection. Although the gastrointestinal and cardiovascular systems are the most frequently affected in MIS-C, respiratory function is also commonly compromised, with a wide spectrum of consequences, from simple cough and shortness of breath to a requirement for mechanical ventilation.^5^^,^^7^

The overlap between the epigenomic landscapes that we found to be associated with MIS-C and Kawasaki disease might have also consequences for understanding the mechanisms involved in the onset of both conditions.^33^ Our findings are in line with the reported appearance of Kawasaki's disease-like features in at least 40% of MIS-C patients.^5^ MIS-C patients with Kawasaki disease-like features are frequently under 5 years of age,^5^ similar to the age of Kawasaki disease patients. In fact, 35% of our patients were younger than 5 years old. The high degree of enrichment of the EPIMISC signature in Kawasaki disease reinforces the invoked role of viral infection in this disorder, as it is also suggested by the peak in cases following the 2009 influenza A H1N1 pandemic.^32^ Since MIS-C and Kawasaki disease have similar underlying DNA methylation defects, epigenetic drugs combined with immunomodulatory agents, targeting viral mimicry and inflammation, could be assessed.

Limitations of the study are mainly associated with sample availability, since MIS-C is a rare and novel disease. First, the number of cases is relatively low, although it is in line with previous studies defining molecular profiles in MIS-C.^27^^,^^40^, ^41^, ^42^, ^43^, ^44^, ^45^, ^46^, ^47^ It should be highlighted, to the best of our knowledge, this is the first epigenomic profiling of MIS-C cases. A second limitation is the lack of ethnic heterogeneity, directly related to the ethnic distribution in the studied population, enriched in West-Eurasia origin. This fact could underestimate key intrinsic features of other populations showing potential enhanced risk of MIS-C in previous studies, such as black children.^7^^,^^48^ A third limitation to consider is the possible existence of additional unmeasured confounding factor, other than age or those that were not statistically significant in our analysis.

In conclusion, we report that MIS-C patients exhibit a well-defined set of epigenetic loci that are associated with the diagnosis of the disorder and support a direct role of a hyperactivated immune response in the characteristic features of overinflammation and multisystem organ involvement. These DNA methylation sites were used to construct an epigenomic signature, EPIMISC, that is associated with the disease. This profile was absent in non-viral inflammatory processes in children, but present to a certain degree in severe adult COVID-19 cases. The profile overlaps with that of another inflammatory syndrome, Kawasaki disease, where a trigger by viral infection can now also be further strengthened. These findings provide essential clues that will help us to understand the immune mechanisms that go awry in MIS-C cases, to identify patients likely to have worse outcomes, and to suggest actionable candidate genes for more specific treatments. Together with genetic, serological and clinical parameters,^49^ the EPIMISC signature could help in patient stratification and to identify highly susceptible patients who require close attention and early active treatments to prevent the progression of the disease. Most importantly, we have identified new biomarkers for diagnosing MIS-C patients that could be useful as the COVID-19 pandemic progresses and seroconversion increases, reducing the value of knowing the history of exposure and serology for defining the MIS-C clinical entity, a key point once COVID-19 turns into an endemic disease worldwide. Finally, the identified epigenetic sites could be useful for following up these patients, including how we monitor the efficacy of immunomodulation therapies and how we can detect at an early stage the MIS-C cases whose disease will rapidly worsen.

## Contributors

VD, CAGP, AP, and ME designed the study, contributed to the analysis, and wrote the first draft of the manuscript. In-depth clinical and pathological characterization and recruitment of patients were carried out by GF, SAA, JVR, ARP, MR, LPS, IJ, IA, PFP, VC, VF, MTV, CR, MMH, ELG, RC, JGR, PSP and AP. All authors helped draft the manuscript or revised it critically for significant intellectual content, and made substantial contributions to the concept and design of the study, and to the acquisition, analysis and interpretation of data.

## Data sharing statement

The complete DNA methylation raw data of the all the studied MIS-C and non-MIS-C samples cases are available on the GEO repository under accession number GSE193879.

## Declaration of interests

Dr. Esteller declares grants from Ferrer International, personal fees from Quimatryx, outside the submitted work. Dr. Rivière reports personal fees from Grifols, CSL behring and Takeda, outside the submitted work. The other authors declare no conflicts of interest.

## Appendix Supplementary materials

Image, application 1

Image, application 2

## References

[1] Zhu N, Zhang D, Wang W (2020). A novel coronavirus from patients with pneumonia in China, 2019. N Engl J Med.

[2] Zhou F, Yu T, Du R (2020). Clinical course and risk factors for mortality of adult inpatients with COVID-19 in Wuhan, China: a retrospective cohort study. Lancet.

[3] Götzinger F, Santiago-García B, Noguera-Julián A (2020). COVID-19 in children and adolescents in Europe: a multinational, multicentre cohort study. Lancet Child Adolesc Health.

[4] Whittaker E, Bamford A, Kenny J (2020). Clinical characteristics of 58 children with a pediatric inflammatory multisystem syndrome temporally associated with SARS-CoV-2. JAMA.

[5] Feldstein LR, Rose EB, Horwitz SM (2020). Multisystem inflammatory syndrome in U.S. children and adolescents. N Engl J Med.

[6] Jiang L, Tang K, Levin M (2020). COVID-19 and multisystem inflammatory syndrome in children and adolescents. Lancet Infect Dis.

[7] Abrams JY, Oster ME, Godfred-Cato SE (2021). Factors linked to severe outcomes in multisystem inflammatory syndrome in children (MIS-C) in the USA: a retrospective surveillance study. Lancet Child Adolesc Health.

[8] Penner J, Abdel-Mannan O, Grant K (2021). 6-month multidisciplinary follow-up and outcomes of patients with paediatric inflammatory multisystem syndrome (PIMS-TS) at a UK tertiary paediatric hospital: a retrospective cohort study. Lancet Child Adolesc Health.

[9] Harwood R, Allin B, Jones CE (2021). A national consensus management pathway for paediatric inflammatory multisystem syndrome temporally associated with COVID-19 (PIMS-TS): results of a national Delphi process. Lancet Child Adolesc Health.

[10] Ahmed M, Advani S, Moreira A (2020). Multisystem inflammatory syndrome in children: a systematic review. EClinicalMedicine.

[11] Berdasco M, Esteller M. (2019). Clinical epigenetics: seizing opportunities for translation. Nat Rev Genet.

[12] Castro de Moura M, Davalos V, Planas-Serra L (2021). Epigenome-wide association study of COVID-19 severity with respiratory failure. EBioMedicine.

[13] Villanueva L, Álvarez-Errico D, Esteller M. (2020). The contribution of epigenetics to cancer immunotherapy. Trends Immunol.

[14] Scott CL, Omilusik KD. (2019). ZEBs: novel players in immune cell development and function. Trends Immunol.

[15] Hamann J, Hsiao CC, Lee CS, Ravichandran KS, Lin HH. (2016). Adhesion GPCRs as modulators of immune cell function. Handb Exp Pharmacol.

[16] O'Connell P, Amalfitano A, Aldhamen YA. (2019). SLAM family receptor signaling in viral infections: HIV and beyond. Vaccines (Basel).

[17] Aldhamen YA, Seregin SS, Schuldt NJ (2012). Vaccines expressing the innate immune modulator EAT-2 elicit potent effector memory T lymphocyte responses despite pre-existing vaccine immunity. J Immunol.

[18] Curtis VF, Ehrentraut SF, Campbell EL (2015). Stabilization of HIF through inhibition of Cullin-2 neddylation is protective in mucosal inflammatory responses. FASEB J.

[19] Kumari P, Russo AJ, Shivcharan S, Rathinam VA. (2020). AIM2 in health and disease: Inflammasome and beyond. Immunol Rev.

[20] Li X, Zhao X, Xing J, Li J (2020). Different epigenome regulation and transcriptome expression of CD4+ and CD8+ T cells from monozygotic twins discordant for psoriasis. Australas J Dermatol.

[21] Imran S, Neeland MR, Koplin J (2021). Epigenetic programming underpins B-cell dysfunction in peanut and multi-food allergy. Clin Transl Immunol.

[22] Amoroso A, Magistroni P, Vespasiano F (2021). HLA and AB0 polymorphisms may influence SARS-CoV-2 infection and COVID-19 severity. Transplantation.

[23] Anzurez A, Naka I, Miki S (2021). Association of HLA-DRB1*09:01 with severe COVID-19. HLA.

[24] Castelli EC, de Castro MV, Naslavsky MS (2021). MHC Variants associated with symptomatic versus asymptomatic SARS-CoV-2 infection in highly exposed individuals. Front Immunol.

[25] Li S, Wu B, Ling Y (2021). Epigenetic landscapes of single-cell chromatin accessibility and transcriptomic immune profiles of T Cells in COVID-19 patients. Front Immunol.

[26] Mousa M, Vurivi H, Kannout H (2021). Genome-wide association study of hospitalized COVID-19 patients in the United Arab Emirates. EBioMedicine.

[27] Chou J, Platt CD, Habiballah S (2021). Mechanisms underlying genetic susceptibility to multisystem inflammatory syndrome in children (MIS-C). J Allergy Clin Immunol.

[28] Junqueira C, Crespo Â, Ranjbar S (2022). FcγR-mediated SARS-CoV-2 infection of monocytes activates inflammation. Nature.

[29] Konigsberg IR, Barnes B, Campbell M (2021). Host methylation predicts SARS-CoV-2 infection and clinical outcome. Commun Med (Lond).

[30] Oriol-Tordera B, Berdasco M, Llano A (2020). Methylation regulation of antiviral host factors, interferon stimulated genes (ISGs) and T-cell responses associated with natural HIV control. PLoS Pathog.

[31] Verdoni L, Mazza A, Gervasoni A (2020). An outbreak of severe Kawasaki-like disease at the Italian epicentre of the SARS-CoV-2 epidemic: an observational cohort study. Lancet.

[32] Ouldali N, Pouletty M, Mariani P (2020). Emergence of Kawasaki disease related to SARS-CoV-2 infection in an epicentre of the French COVID-19 epidemic: a time-series analysis. Lancet Child Adolesc Health.

[33] Sancho-Shimizu V, Brodin P, Cobat A (2021). SARS-CoV-2-related MIS-C: A key to the viral and genetic causes of Kawasaki disease?. J Exp Med.

[34] Rodriguez-Smith JJ, Verweyen EL, Clay GM (2021). Inflammatory biomarkers in COVID-19 associated multisystem inflammatory syndrome in children, Kawasaki disease, and macrophage activation syndrome: a cohort study. Lancet Rheumatol.

[35] Chen KD, Huang YH, Ming-Huey Guo M (2018). The human blood DNA methylome identifies crucial role of β-catenin in the pathogenesis of Kawasaki disease. Oncotarget.

[36] Wang J, Farkas C, Benyoucef A (2021). Interplay between the EMT transcription factors ZEB1 and ZEB2 regulates hematopoietic stem and progenitor cell differentiation and hematopoietic lineage fidelity. PLoS Biol.

[37] Huang LH, Kuo HC, Pan CT, Lin YS, Huang YH, Li SC. (2018). Multiomics analyses identified epigenetic modulation of the S100A gene family in Kawasaki disease and their significant involvement in neutrophil transendothelial migration. Clin Epigenet.

[38] Reinartz A, Ehling J, Franz S (2010). Small intestinal mucosa expression of putative chaperone fls485. BMC Gastroenterol.

[39] Ovsyannikova IG, Dhiman N, Haralambieva IH (2010). Rubella vaccine-induced cellular immunity: evidence of associations with polymorphisms in the Toll-like, vitamin A and D receptors, and innate immune response genes. Hum Genet.

[40] Ramaswamy A, Brodsky NN, Sumida TS (2021). Immune dysregulation and autoreactivity correlate with disease severity in SARS-CoV-2-associated multisystem inflammatory syndrome in children. Immunity.

[41] Porritt RA, Binek A, Paschold L (2021). The autoimmune signature of hyperinflammatory multisystem inflammatory syndrome in children. J Clin Invest.

[42] Beckmann ND, Comella PH, Cheng E (2021). Downregulation of exhausted cytotoxic T cells in gene expression networks of multisystem inflammatory syndrome in children. Nat Commun.

[43] Pfeifer J, Thurner B, Kessel C (2022). Autoantibodies against interleukin-1 receptor antagonist in multisystem inflammatory syndrome in children: a multicentre, retrospective, cohort study. Lancet Rheumatol.

[44] Gruber CN, Patel RS, Trachtman R (2020). Mapping systemic inflammation and antibody responses in multisystem inflammatory syndrome in children (MIS-C). Cell.

[45] Consiglio CR, Cotugno N, Sardh F (2020). The immunology of multisystem inflammatory syndrome in children with COVID-19. Cell.

[46] Vella LA, Giles JR, Baxter AE (2021). Deep immune profiling of MIS-C demonstrates marked but transient immune activation compared to adult and pediatric COVID-19. Sci Immunol.

[47] Esteve-Sole A, Anton J, Pino-Ramirez RM (2021). Similarities and differences between the immunopathogenesis of COVID-19-related pediatric multisystem inflammatory syndrome and Kawasaki disease. J Clin Invest.

[48] Middelburg JG, Crijnen TEM, D'Antiga L (2021). Association of ethnicity with multisystem inflammatory syndrome in children related to SARS-CoV-2 infection: an international case-referent study. Front Pediatr.

[49] Geva A, Patel MM, Newhams MM (2021). Data-driven clustering identifies features distinguishing multisystem inflammatory syndrome from acute COVID-19 in children and adolescents. EClinical Medicine.

