# Supplementary material for: Epigenetic profiling linked to multisystem inflammatory syndrome in children (MIS-C): A multicenter, retrospective study
Source: eClinicalMedicine. 2022 Jun 25;50:101515. doi: 10.1016/j.eclinm.2022.101515 (PMC9233426; doi:10.1016/j.eclinm.2022.101515)
Supplement: Supplementary file 1 [file mmc1.docx]

**Supplementary Materials**

**Supplementary Figure S1.** Graphical schema representing the populations of interest and the screening strategy used to identify epigenetic biomarkers of MIS-C.

**Supplementary Figure S2.** Characteristic laboratory parameters of the MIS-C patients included in the study.

**Supplementary Figure S3.** EPIMISC performance in the discovery cohort.

**Supplementary Figure S4.** Heatmap of the discovery cohort samples, clustered by methylation beta values of the 33 CpGs defining the EPIMISC signature.

**Supplementary Figure S5.** EPIMISC performance in the validation cohort.

**Supplementary Figure S6.** Heatmap of the validation cohort samples, clustered by methylation beta values of the 33 CpGs defining the EPIMISC signature.

**Supplementary Figure S7.** EPIMISC performance in the entire cohort.

**Supplementary Table S1.** Characteristics of the pediatric COVID-19 patients with IgG and/or PCR positive status for SARS-CoV-2, but without MIS-C.

**Supplementary Table S2.** Characteristics of the children and adolescent control donors collected during the pre-COVID-19 period.

**Supplementary Table S3.** Description of the 33 CpG sites with a differential DNA methylation status between the MIS-C and non-MIS-C in the discovery cohort, according to the study pipeline described in Supplementary Figure S1.

**Supplementary Table S4.** Description of the 1337 CpG sites in the 47 genes related to COVID-19, based on published studies.

**Supplementary Table S5.** Description of the 68 CpG sites in the 3 genes related to MIS-C, based on published studies.

**Supplementary Table S6.** EPIMISC derived-CpGs in the validation and entire cohorts, according to the study pipeline described in Supplementary Figure S1.

**Supplementary Table S7.** Description of the 1350 CpG sites in the 33 genes related to Kawasaki disease, based on published studies.

**Supplementary Methods.** Study protocol and extended methods.
