# Supplementary material for: Epigenetic profiling linked to multisystem inflammatory syndrome in children (MIS-C): A multicenter, retrospective study
Source: eClinicalMedicine. 2022 Jun 25;50:101515. doi: 10.1016/j.eclinm.2022.101515 (PMC9233426; doi:10.1016/j.eclinm.2022.101515)
Supplement: Supplementary file 2 [file mmc2.pdf]

Supplementary Materials

Supplementary Figure S1. Graphical schema representing the populations of interest and the screening strategy used to identify epigenetic biomarkers of MIS-C.

Supplementary Figure S2. Characteristic laboratory parameters of the MIS-C patients included in the study.

Supplementary Figure S3. EPIMISC performance in the discovery cohort.

Supplementary Figure S4. Heatmap of the discovery cohort samples, clustered by methylation beta values of the 33 CpGs defining the EPIMISC signature.

Supplementary Figure S5. EPIMISC performance in the validation cohort.

Supplementary Figure S6. Heatmap of the validation cohort samples, clustered by methylation beta values of the 33 CpGs defining the EPIMISC signature.

Supplementary Figure S7. EPIMISC performance in the entire cohort.

Supplementary Table S1. Characteristics of the pediatric COVID-19 patients with IgG and/or PCR positive status for SARS-CoV-2, but without MIS-C.

Supplementary Table S2. Characteristics of the children and adolescent control donors collected during the pre-COVID-19 period.

Supplementary Table S3. Description of the 33 CpG sites with a differential DNA methylation status between the MIS-C and non-MIS-C in the discovery cohort, according to the study pipeline described in Supplementary Figure S1.

Supplementary Table S4. Description of the 1337 CpG sites in the 47 genes related to COVID-19, based on published studies.

Supplementary Table S5. Description of the 68 CpG sites in the 3 genes related to MIS-C, based on published studies.

Supplementary Table S6. EPIMISC derived-CpGs in the validation and entire cohorts, according to the study pipeline described in Supplementary Figure S1.

Supplementary Table S7. Description of the 1350 CpG sites in the 33 genes related to Kawasaki disease, based on published studies.

Supplementary Methods. Study protocol and extended methods.

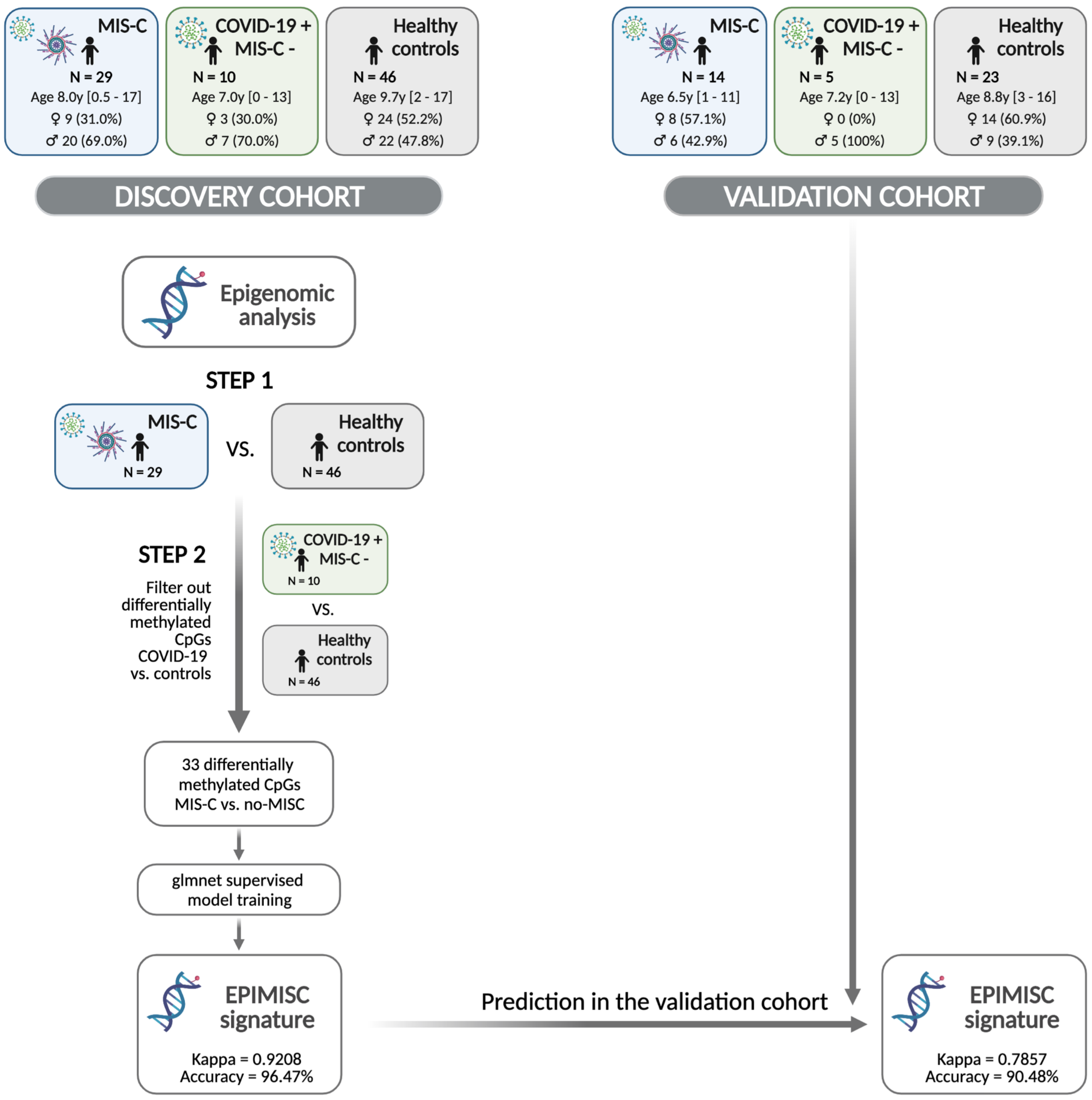

Supplementary Figure S1. Graphical schema representing the populations of interest and the screening strategy used to identify epigenetic biomarkers of MIS-C.

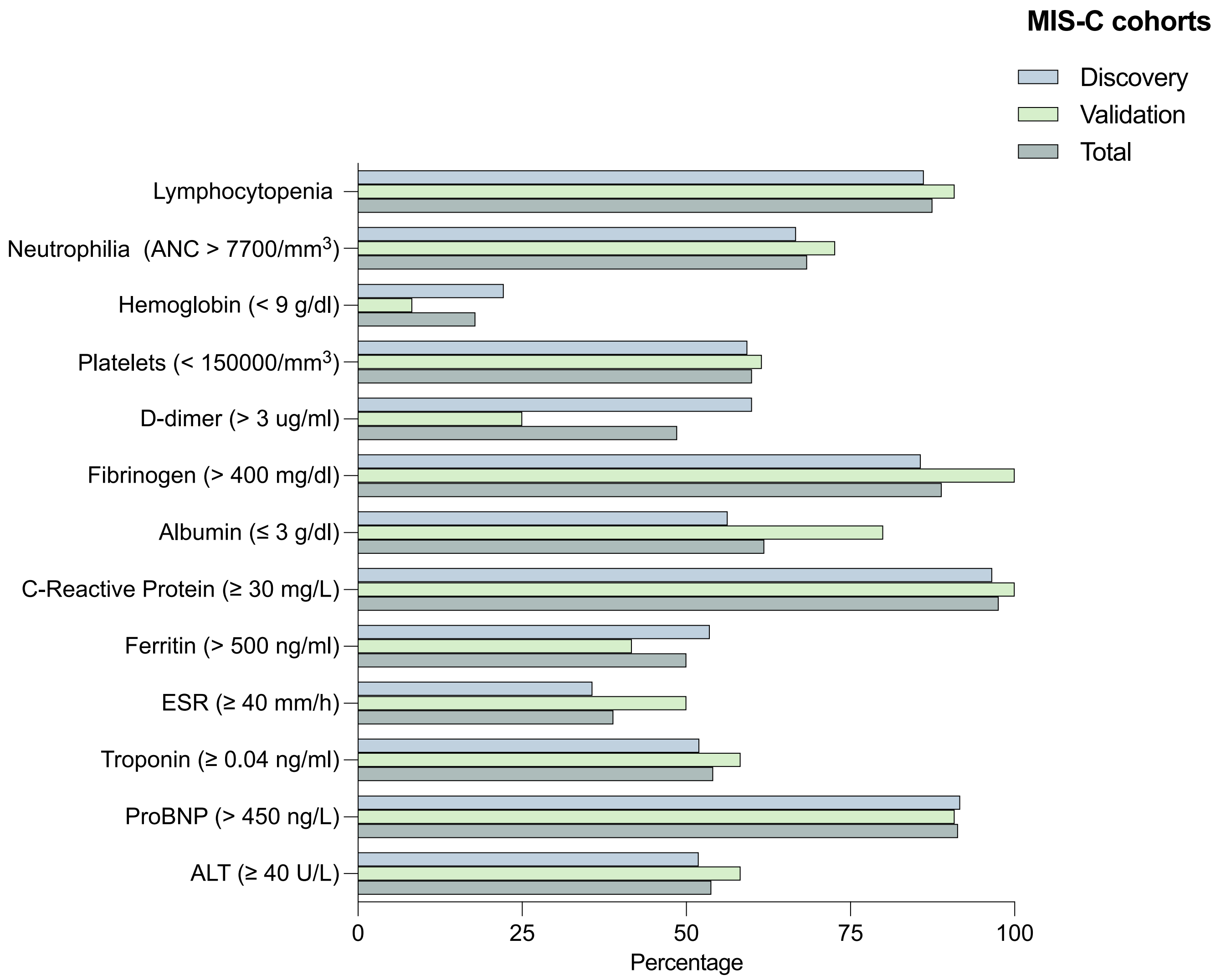

Supplementary Figure S2. Characteristic laboratory parameters of the MIS-C patients included in the study. Following the criteria used for MIS-C patients in Feldstein et al., *N Engl J Med*, 2020. Lymphocytopenia was defined as an absolute lymphocyte count (ALC) of less than 1500 per microliter in patients 8 months of age or older, and less than 4500 per microliter in patients younger than 8 months of age. Abbreviations: ALT, Alanine aminotransferase; ANC, Absolute neutrophil count; ESR, Erythrocyte sedimentation rate; ProBNP, pro-brain natriuretic peptide.

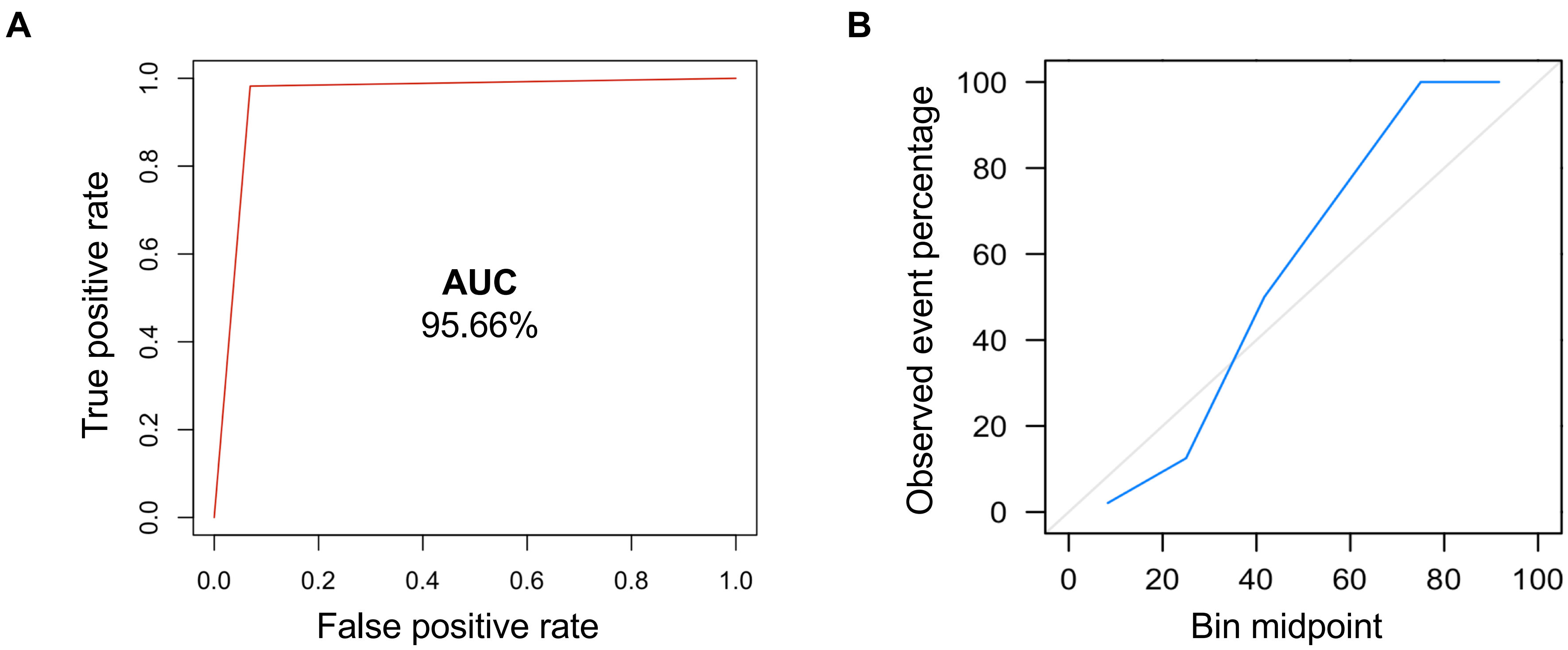

Supplementary Figure S3. EPIMISC performance in the discovery cohort. **A.** Receiver Operating Characteristic (ROC) curve and Area Under the Curve (AUC = 95.66% (95% confidence interval = 90.65% to 100%)) for the EPIMISC signature. **B.** Calibration curve using 6 bins to characterize the consistency between EPIMISC predicted class probabilities and observed event rates.

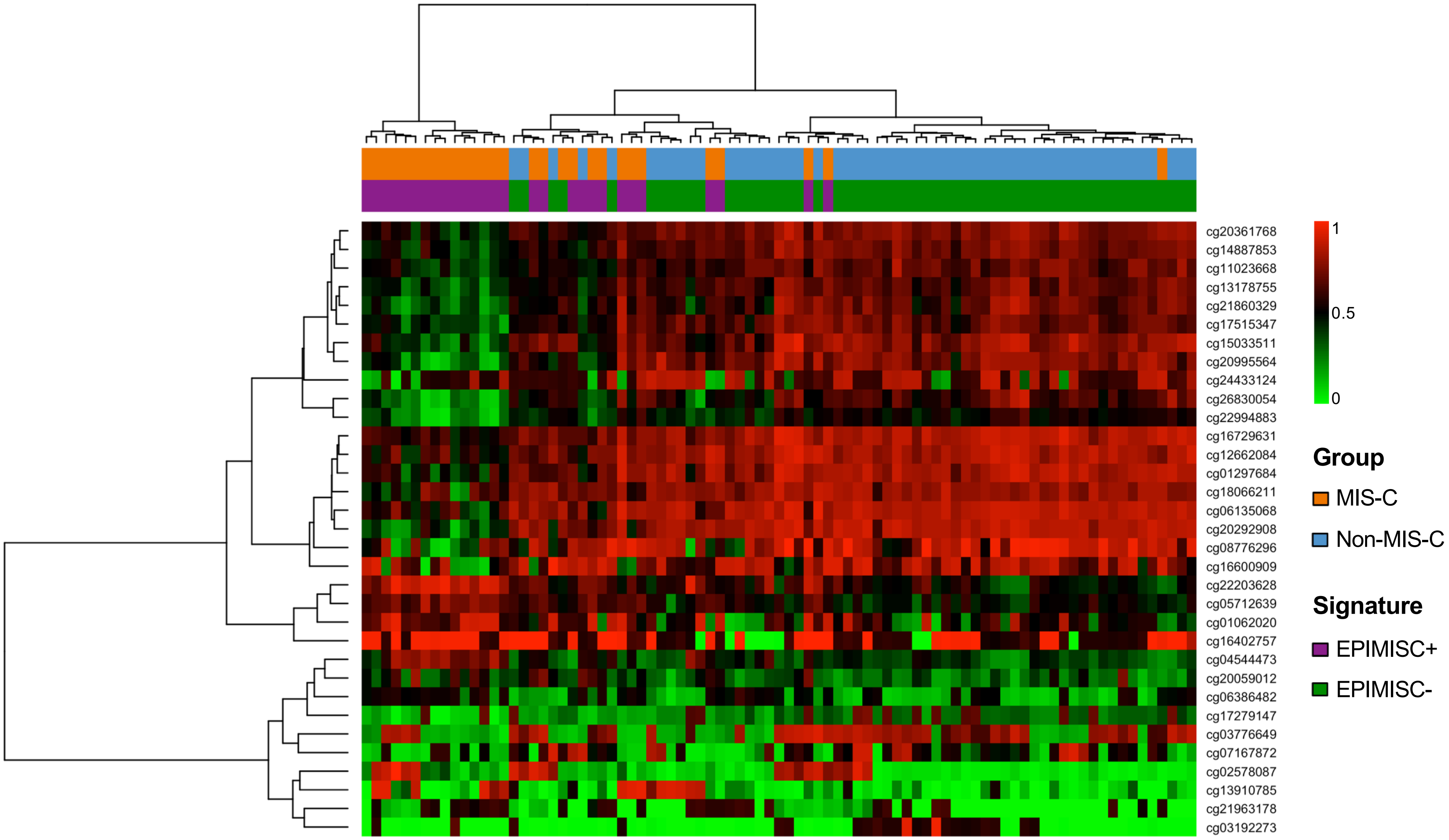

Supplementary Figure S4. Heatmap of the discovery cohort samples, clustered by methylation beta values of the 33 CpGs defining the EPIMISC signature. Cluster analysis was performed using the Ward.D clustering method and assuming Manhattan distances.

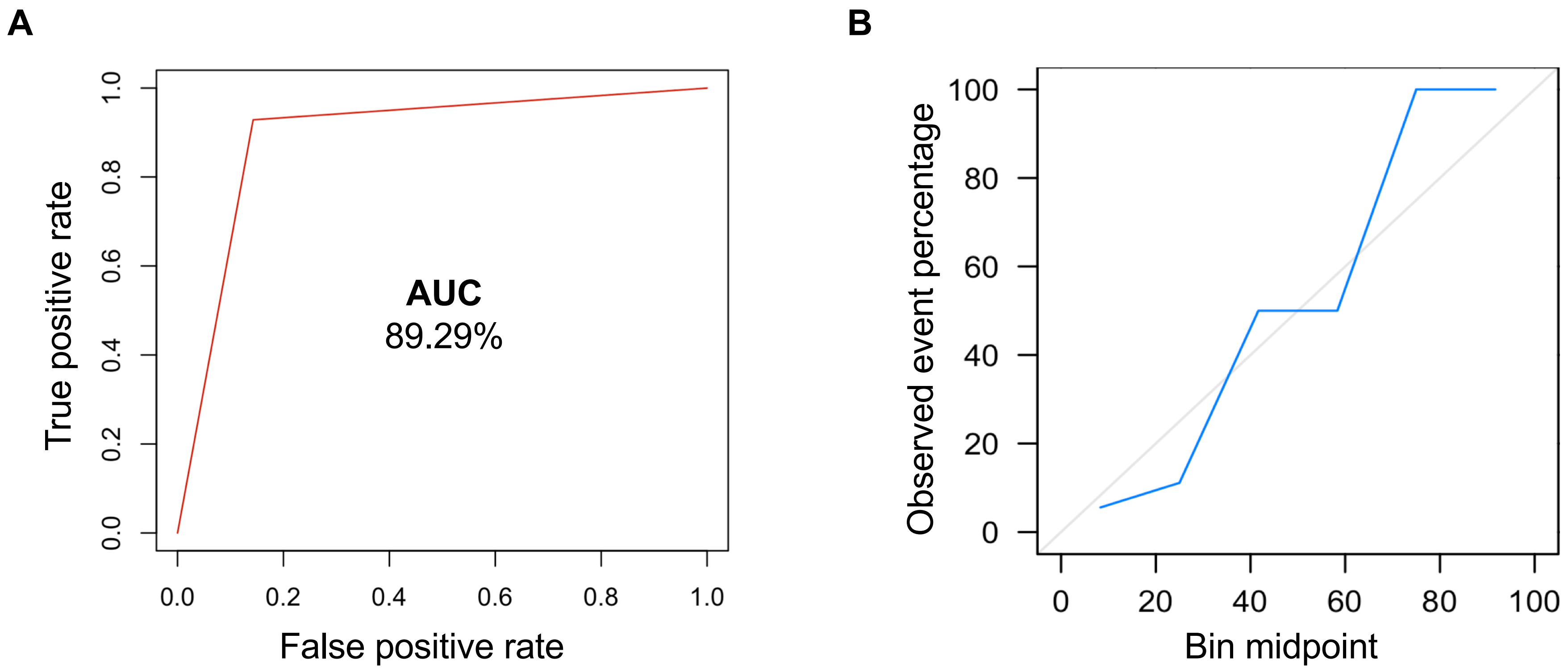

Supplementary Figure S5. EPIMISC performance in the validation cohort. **A.** Receiver Operating Characteristic (ROC) curve and Area Under the Curve (AUC = 89.29% (95% confidence interval = 78.61% to 99.97%)) for the EPIMISC signature. **B**. Calibration curve using 6 bins to characterize the consistency between EPIMISC predicted class probabilities and observed event rates.

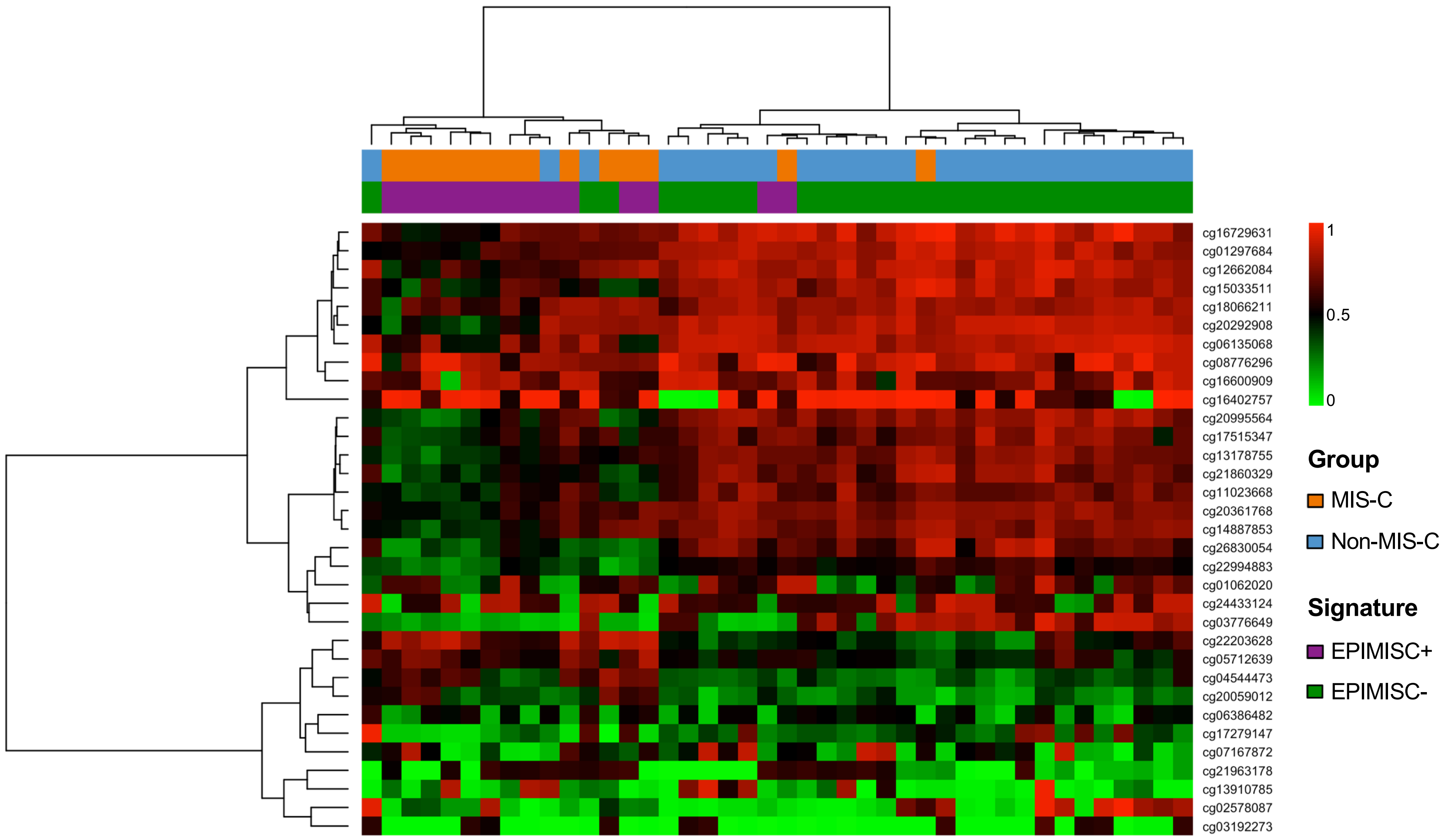

Supplementary Figure S6. Heatmap of the validation cohort samples, clustered by methylation beta values of the 33 CpGs defining the EPIMISC signature. Cluster analysis was performed using the Ward.D clustering method and assuming Manhattan distances.

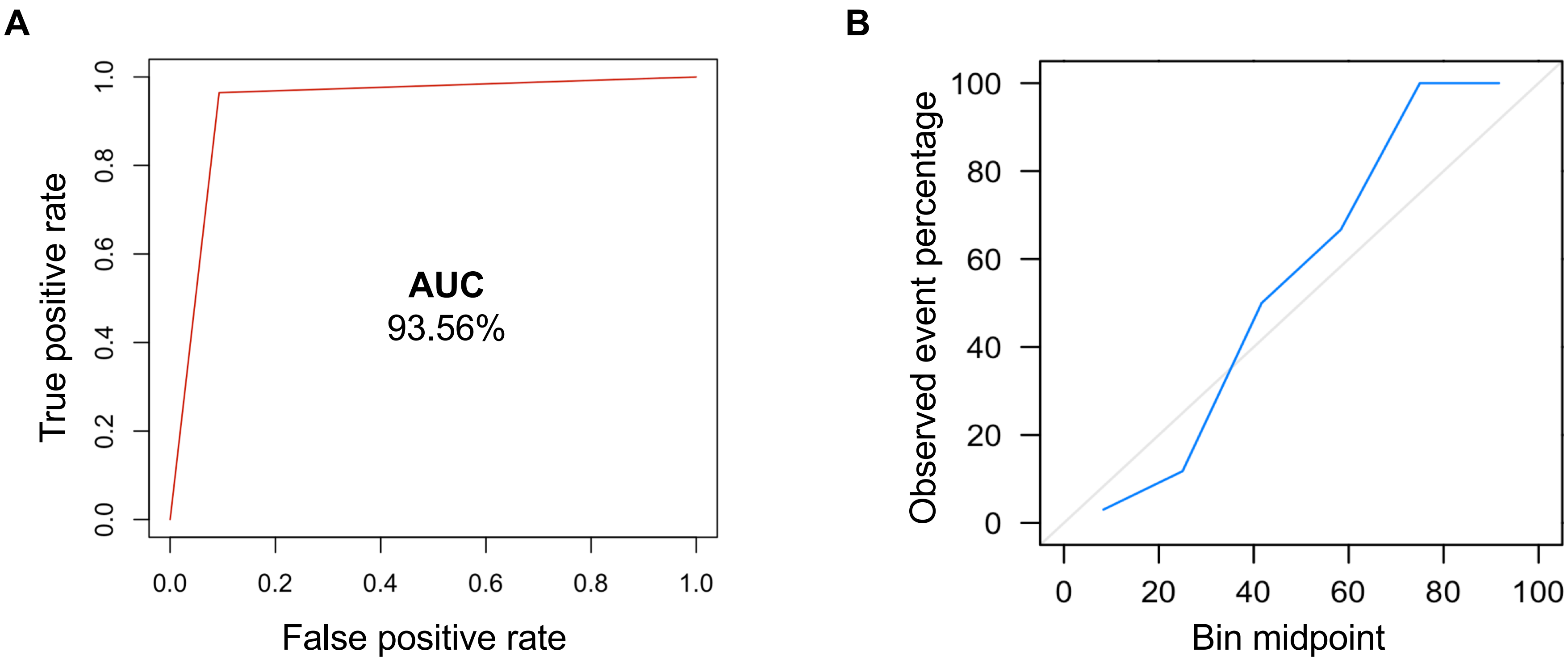

Supplementary Figure S7. EPIMISC performance in the entire cohort. **A.** Receiver Operating Characteristic (ROC) curve and Area Under the Curve (AUC = 93.56% (95% confidence interval = 88.74% to 98.39%)) for the EPIMISC signature. **B**. Calibration curve using 6 bins to characterize the consistency between EPIMISC predicted class probabilities and observed event rates.

Supplementary Table S1. Characteristics of the pediatric COVID-19 patients with IgG and/or PCR positive status for SARS-CoV-2, but without MIS-C.

Characteristics	Pediatric COVID-19 cohorts		
	**Discovery cohort** (N = 10)	**Validation cohort** (N = 5)	**Entire cohort** (N = 15)
**Gender** - Frequency (%)			
Female	3 (30.0%)	0 (0%)	3 (20.0%)
Male	7 (70.0%)	5 (100%)	12 (80.0%)
**Age (years)** - Median [range]	7.0 [0 - 13]	7.2 [0 - 13]	7.1 [0 - 13]
**Age group**- Frequency (%)			
≤ 2 yr	2 (20.0%)	2 (40.0%)	4 (26.7%)
3 – 5 yr	1 (10.0%)	0 (0%)	1 (6.7%)
6 – 9 yr	5 (50.0%)	1 (20.0%)	6 (40.0%)
10 – 13 yr	2 (20.0%)	2 (40.0%)	4 (26.7%)
**Ethnicity** - Frequency (%)			
West-Eurasia	10 (100%)	3 (60.0%)	13 (86.7%)
Central-South America	0 (0%)	1 (20.0%)	1 (6.7%)
African	0 (0%)	1 (20.0%)	1 (6.7%)
**SARS-CoV-2 status**- Frequency (%)			
IgG and/or PCR positive	10 (100%)	5 (100%)	15 (100%)
**Highest level of care** - Frequency (%)			
Home	2 (20.0%)	1 (20.0%)	3 (20.0%)
Ward	4 (40.0%)	2 (40.0%)	6 (40.0%)
Intensive care unit	4 (40.0%)	2 (40.0%)	6 (40.0%)
**Oxygen supplementation** - Frequency (%)			
None	3 (30.0%)	3 (60.0%)	6 (40.0%)
Nasal cannula	4 (40.0%)	0 (0%)	4 (26.7%)
Non-Invasive Ventilation or High Flow Oxygen	3 (30.0%)	1 (20.0%)	4 (26.7%)
Mechanical Ventilation	0 (0%)	1 (20.0%)	1 (6.7%)

Supplementary Table S2. Characteristics of the children and adolescent control donors collected during the pre-COVID-19 period.

Characteristics		Pediatric control donor cohorts		
		**Discovery cohort** (N = 46)	**Validation cohort** (N = 23)	**Entire cohort** (N = 69)
**Gender** - Frequency (%)				
	Female	24 (52.2%)	14 (60.9%)	38 (55.1%)
	Male	22 (47.8%)	9 (39.1%)	31 (44.9%)
**Age (years)** - Median [range]		9.7 [2 - 17]	8.8 [3 - 16]	9.4 [2 - 17]
**Age group**- Frequency (%)				
	≤ 2 yr	4 (8.7%)	0 (0%)	4 (5.8%)
	3 – 5 yr	6 (13.0%)	8 (34.8%)	14 (20.3%)
	6 – 9 yr	10 (21.7%)	6 (26.1%)	16 (23.2%)
	10 – 13 yr	13 (28.3%)	2 (8.7%)	15 (21.7%)
	14 – 18 yr	13 (28.3%)	7 (30.4%)	20 (29.0%)
**Ethnicity** - Frequency (%)				
	West-Eurasia	31 (67.4%)	11 (47.8%)	42 (60.9%)
	Central-South America	1 (2.2%)	1 (4.3%)	2 (2.9%)
	African	1 (2.2%)	0 (20.0%)	1 (1.4%)
	Unknown	13 (28.2%)	11 (47.8%)	24 (34.7%)
**Sampling date** - Frequency (%)				
	Pre-COVID-19 pandemic (before December 2019)	46 (100%)	23 (100%)	69 (100%)

Supplementary Table S3. Description of the 33 CpG sites with a differential DNA methylation status between MIS-C and non-MIS-C in the discovery cohort, according to the study pipeline described in Supplementary Figure S1.

CpG ID	Chromosome location	Gene name	P value	Absolute mean methylation difference
cg20292908	chr5:172203421		< 0.001	0.30
cg20995564	chr2:145172035	ZEB2	< 0.001	0.27
cg06135068	chr16:31034016		< 0.001	0.22
cg16402757	chr10:35311004	CUL2	0.010	0.21
cg26830054	chr10:119762394		< 0.001	0.20
cg15033511	chr17:54994023		< 0.001	0.20
cg01062020	chr1:162382848	SH2D1B	0.001	0.19
cg03776649	chr5:125465533		0.023	0.19
cg22203628	chr11:69241075		< 0.001	0.19
cg02578087	chr3:8671361	SSUH2	0.009	0.18
cg01297684	chr12:56069634		< 0.001	0.18
cg20361768	chr3:156819083	LINC00880	< 0.001	0.18
cg06386482	chr6:47624117	GPR111	< 0.001	0.18
cg21963178	chr10:75571738	NDST2	0.001	0.18
cg24433124	chr6:30755968		0.004	0.17
cg13910785	chr6:32549849	HLA-DRB1	0.042	0.17
cg05712639	chr14:52819386		< 0.001	0.17
cg03192273	chr5:150618948		0.028	0.16
cg21860329	chr13:42265546	VWA8	< 0.001	0.16
cg14887853	chr6:139794538	LOC645434	< 0.001	0.16
cg12662084	chr6:17809126	KIF13A	< 0.001	0.16
cg16600909	chr1:173145001		0.012	0.15
cg13178755	chr2:174023580	ZAK	< 0.001	0.15
cg20059012	chr12:53613154	RARG	< 0.001	0.15
cg07167872	chr1:205819463	PM20D1	0.033	0.15
cg16729631	chr8:131000261		< 0.001	0.15
cg17515347	chr1:159047163	AIM2	< 0.001	0.15
cg11023668	chr2:25095040	ADCY3	< 0.001	0.15
cg22994883	chr7:130615334		< 0.001	0.15
cg04544473	chr4:153021978	LOC100996286	< 0.001	0.15
cg18066211	chr4:99369082		< 0.001	0.15
cg17279147	chr6:14739369		0.001	0.15
cg08776296	chr7:134856544	CYREN	0.014	0.15

Notes: CpG ID corresponds to the unique CpG site identifier in the HumanMethylationEPIC array (Illumina). Chromosomal location denoted according human reference assembly GRCh37/hg19. All depicted P values are FDR adjusted P values. CpG-sites with an absolute mean methylation beta value difference > 0.15 and FDR adjusted P value < 0.05 were considered statistically significant.

Supplementary Table S4. Description of the 1337 CpG sites in the 47 genes related to COVID-19, based on published studies*.

CpG ID	Chromosome location	Gene name	P value	Absolute mean methylation difference
cg23232263	chrX:15579482	ACE2	0.015	0.052
cg05039749	chrX:15583512	ACE2	0.016	0.021
cg22422976	chrX:15584867	ACE2	0.076	0.087
cg25176872	chrX:15584988	ACE2	0.158	0.033
cg16716680	chrX:15585368	ACE2	0.044	0.094
cg04013915	chrX:15586143	ACE2	0.120	0.080
cg20119767	chrX:15612969	ACE2	0.448	0.026
cg05241917	chrX:15616197	ACE2	0.778	0.005
cg10408040	chrX:15619136	ACE2	0.047	0.023
cg05748796	chrX:15619337	ACE2	0.899	0.000
cg16734967	chrX:15620103	ACE2	0.259	0.023
cg08559914	chrX:15620240	ACE2	0.009	0.013
cg03536816	chrX:15621042	ACE2	0.018	0.075
cg18877734	chrX:15621084	ACE2	0.931	0.000
cg21598868	chrX:15621167	ACE2	0.340	0.032
cg18458833	chrX:15621477	ACE2	0.313	0.006
cg19776619	chr19:569801	BSG	0.640	0.001
cg04015604	chr19:569965	BSG	0.575	0.002
cg26529300	chr19:570458	BSG	0.308	0.031
cg01532103	chr19:571107	BSG	0.564	0.002
cg17382033	chr19:571114	BSG	0.698	0.001
cg27641720	chr19:571120	BSG	0.092	0.005
cg02129274	chr19:571177	BSG	0.420	0.002
cg05622915	chr19:571204	BSG	0.266	0.002
cg25660646	chr19:571323	BSG	0.020	0.003
cg17522907	chr19:571416	BSG	0.863	0.000
cg07513435	chr19:571744	BSG	0.807	0.002
cg18341969	chr19:571802	BSG	0.193	0.003
cg10641392	chr19:572279	BSG	0.831	0.001
cg11982443	chr19:572297	BSG	0.021	0.004
cg22431486	chr19:572309	BSG	0.417	0.003
cg21717879	chr19:572359	BSG	0.712	0.001
cg15456960	chr19:572669	BSG	0.588	0.000
cg06894251	chr19:572721	BSG	0.864	0.000
cg06546033	chr19:573090	BSG	0.720	0.002
cg25572267	chr19:573480	BSG	0.357	0.002
cg12953747	chr19:573647	BSG	0.178	0.007
cg01241899	chr19:573758	BSG	0.742	0.001
cg09629510	chr19:574762	BSG	0.009	0.032
cg19271640	chr19:574881	BSG	0.026	0.026

cg03079637	chr19:574899	BSG	0.004	0.033
cg17640894	chr19:574950	BSG	0.020	0.023
cg03233876	chr19:575412	BSG	0.017	0.022
cg23919549	chr19:576047	BSG	0.247	0.004
cg22913585	chr19:577938	BSG	0.193	0.002
cg04698845	chr19:578093	BSG	0.612	0.001
cg00752531	chr19:578440	BSG	0.906	0.000
cg01794046	chr19:578648	BSG	0.059	0.013
cg05475770	chr19:579192	BSG	0.708	0.002
cg11427463	chr19:579361	BSG	0.456	0.015
cg08541599	chr19:579501	BSG	0.252	0.017
cg22842143	chr19:579592	BSG	0.832	0.001
cg06810134	chr19:581326	BSG	0.793	0.002
cg10362365	chr19:581404	BSG	0.936	0.000
cg26721694	chr19:581428	BSG	0.698	0.000
cg08238794	chr19:581564	BSG	0.516	0.004
cg19651003	chr19:583049	BSG	0.050	0.016
cg03830490	chr3:45926669	CCR9	0.936	0.000
cg24603476	chr3:45927105	CCR9	0.024	0.009
cg27198997	chr3:45927589	CCR9	< 0.001	0.017
cg09033997	chr3:45927601	CCR9	0.026	0.006
cg06519172	chr3:45927630	CCR9	0.010	0.012
cg17642041	chr3:45928007	CCR9	0.057	0.021
cg06997537	chr3:45928018	CCR9	0.024	0.031
cg23519441	chr3:45929350	CCR9	0.003	0.035
cg17885452	chr3:45930958	CCR9	0.007	0.039
cg25155774	chr3:45932141	CCR9	0.177	0.007
cg00786084	chr3:45942460	CCR9	0.001	0.041
cg14558191	chr3:45943637	CCR9	0.233	0.003
cg14525884	chr3:45944114	CCR9	0.358	0.003
cg01588525	chr3:45944247	CCR9	0.809	0.000
cg04015765	chr19:7805430	CD209	0.647	0.012
cg26704759	chr19:7807617	CD209	0.027	0.019
cg01452452	chr19:7807999	CD209	0.682	0.003
cg01618851	chr19:7812265	CD209	0.945	0.002
cg21848746	chr19:7812568	CD209	0.149	0.026
cg06122489	chr19:7813167	CD209	0.054	0.016
cg07608333	chr19:7813422	CD209	0.073	0.024
cg02794892	chr19:7813901	CD209	0.274	0.014
cg04412303	chr19:7813963	CD209	0.529	0.010
cg12147572	chr19:7827744	CLEC4M	0.444	0.003
cg07584894	chr19:7827802	CLEC4M	0.353	0.003
cg00480077	chr19:7827894	CLEC4M	0.425	0.003
cg21372914	chr19:7828144	CLEC4M	0.170	0.010
cg18515510	chr19:7831896	CLEC4M	0.907	0.002
cg11615959	chr19:7834128	CLEC4M	0.218	0.004

cg13815445	chr11:88028475	CTSC	0.004	0.025
cg21044820	chr11:88030631	CTSC	0.183	0.004
cg12710531	chr11:88032774	CTSC	0.104	0.004
cg21562812	chr11:88041491	CTSC	0.090	0.004
cg09555097	chr11:88049447	CTSC	0.388	0.012
cg25636441	chr11:88059527	CTSC	0.006	0.010
cg03596479	chr11:88059873	CTSC	0.033	0.030
cg08522340	chr11:88068493	CTSC	0.463	0.041
cg09706192	chr11:88068895	CTSC	0.074	0.043
cg16118839	chr11:88069169	CTSC	< 0.001	0.075
cg09280946	chr11:88070661	CTSC	0.133	0.001
cg16588061	chr11:88070780	CTSC	0.354	0.002
cg15581365	chr11:88071006	CTSC	0.503	0.001
cg01742905	chr11:88071023	CTSC	0.502	0.001
cg11733478	chr11:88071037	CTSC	0.003	0.013
cg12747487	chr11:88071039	CTSC	0.007	0.009
cg11868029	chr11:88071041	CTSC	0.715	0.001
cg25902627	chr11:88071152	CTSC	0.112	0.006
cg24637319	chr11:88071195	CTSC	0.793	0.000
cg12391538	chr11:88071409	CTSC	0.228	0.004
cg15571376	chr11:88071458	CTSC	0.205	0.002
cg03183608	chr11:88071760	CTSC	0.879	0.000
cg13985445	chr9:90340577	CTSL1	0.016	0.022
cg11154542	chr9:90340652	CTSL1	0.023	0.016
cg14236855	chr9:90341041	CTSL1	0.439	0.002
cg15242570	chr9:90341048	CTSL1	0.859	0.002
cg14243623	chr9:90341158	CTSL1	0.118	0.010
cg14557714	chr9:90341385	CTSL1	0.409	0.001
cg11306701	chr9:90341458	CTSL1	0.905	0.003
cg14190128	chr9:90342638	CTSL1	0.402	0.005
cg21177940	chr9:90344705	CTSL1	0.159	0.009
cg08033130	chr3:45983597	CXCR6	0.637	0.031
cg19145607	chr3:45983792	CXCR6	0.794	0.011
cg07983767	chr3:45984158	CXCR6	0.373	0.017
cg03682895	chr3:45984585	CXCR6	0.019	0.007
cg05705212	chr3:45984743	CXCR6	< 0.001	0.037
cg08450017	chr3:45984838	CXCR6	< 0.001	0.107
cg05979583	chr3:45984851	CXCR6	< 0.001	0.101
cg13480043	chr3:45984883	CXCR6	0.012	0.032
cg25226014	chr3:45984942	CXCR6	< 0.001	0.084
cg01178899	chr3:45985168	CXCR6	< 0.001	0.087
cg04544784	chr3:45986281	CXCR6	< 0.001	0.018
cg05963956	chr3:45986287	CXCR6	0.177	0.004
cg26896911	chr3:45986498	CXCR6	< 0.001	0.067
cg26466027	chr3:45989744	CXCR6	0.562	0.012
cg20427486	chr2:162849137	DPP4	0.390	0.007

cg03688421	chr2:162857123	DPP4	0.036	0.013
cg04512966	chr2:162859453	DPP4	0.010	0.033
cg17205440	chr2:162864033	DPP4	0.002	0.027
cg04444758	chr2:162871045	DPP4	0.104	0.011
cg08652858	chr2:162872807	DPP4	0.867	0.000
cg10934831	chr2:162874136	DPP4	0.722	0.001
cg17278689	chr2:162874215	DPP4	0.978	0.002
cg02902394	chr2:162874748	DPP4	0.992	0.001
cg00273390	chr2:162874787	DPP4	0.444	0.003
cg20817669	chr2:162875463	DPP4	< 0.001	0.032
cg02039580	chr2:162878125	DPP4	0.007	0.017
cg08318371	chr2:162881135	DPP4	0.872	0.001
cg24098944	chr2:162885448	DPP4	0.765	0.001
cg00235671	chr2:162886026	DPP4	< 0.001	0.036
cg16172329	chr2:162907368	DPP4	0.374	0.003
cg24094122	chr2:162915893	DPP4	< 0.001	0.061
cg15353603	chr2:162915902	DPP4	< 0.001	0.063
cg03241330	chr2:162920114	DPP4	< 0.001	0.037
cg12649175	chr2:162927003	DPP4	0.486	0.010
cg17714581	chr2:162928426	DPP4	0.888	0.001
cg09316885	chr2:162929318	DPP4	< 0.001	0.042
cg24827032	chr2:162929690	DPP4	0.085	0.026
cg19350270	chr2:162929832	DPP4	0.456	0.014
cg16617101	chr2:162930153	DPP4	0.606	0.007
cg14022057	chr2:162930175	DPP4	0.531	0.005
cg01907512	chr2:162930212	DPP4	0.710	0.001
cg10112391	chr2:162930376	DPP4	0.087	0.007
cg20053454	chr2:162930454	DPP4	0.244	0.002
cg10507402	chr2:162930666	DPP4	0.073	0.001
cg09601770	chr2:162930671	DPP4	0.674	0.001
cg25912827	chr2:162930805	DPP4	0.821	0.000
cg21565914	chr2:162931175	DPP4	0.488	0.013
cg23246095	chr2:162931638	DPP4	0.321	0.005
cg20539283	chr2:162932048	DPP4	0.967	0.000
cg14779065	chr2:162932295	DPP4	0.049	0.010
cg04037813	chr19:4675281	DPP9	0.328	0.004
cg13513411	chr19:4676202	DPP9	0.574	0.001
cg01098142	chr19:4676492	DPP9	0.605	0.002
cg00491603	chr19:4676557	DPP9	0.012	0.013
cg18328612	chr19:4676623	DPP9	0.920	0.001
cg18317135	chr19:4677230	DPP9	0.906	0.001
cg07230018	chr19:4677282	DPP9	0.825	0.004
cg09508448	chr19:4679533	DPP9	0.012	0.013
cg03775330	chr19:4682727	DPP9	0.697	0.001
cg15544596	chr19:4683273	DPP9	0.731	0.002
cg10344433	chr19:4684712	DPP9	0.010	0.006

cg22519738	chr19:4684891	DPP9	0.758	0.000
cg22521640	chr19:4685815	DPP9	0.951	0.001
cg22768335	chr19:4687000	DPP9	0.610	0.006
cg14531512	chr19:4687389	DPP9	0.845	0.001
cg03247293	chr19:4687469	DPP9	0.073	0.010
cg25730440	chr19:4688549	DPP9	0.542	0.003
cg02907425	chr19:4688820	DPP9	0.274	0.005
cg15746870	chr19:4689573	DPP9	0.738	0.001
cg13514178	chr19:4689839	DPP9	0.154	0.007
cg09356750	chr19:4691020	DPP9	0.233	0.005
cg14477543	chr19:4691599	DPP9	0.764	0.001
cg00163735	chr19:4692258	DPP9	0.384	0.005
cg19526579	chr19:4692861	DPP9	0.170	0.005
cg00503832	chr19:4693592	DPP9	0.784	0.001
ch.19.253092F	chr19:4694205	DPP9	0.045	0.006
cg23356090	chr19:4695683	DPP9	0.039	0.021
cg24142638	chr19:4697453	DPP9	0.415	0.006
cg15355185	chr19:4700434	DPP9	0.530	0.004
cg21853992	chr19:4700601	DPP9	0.102	0.007
cg03734301	chr19:4702458	DPP9	0.471	0.003
cg01152629	chr19:4705838	DPP9	0.700	0.003
cg07204134	chr19:4707215	DPP9	0.307	0.003
cg10165241	chr19:4709493	DPP9	< 0.001	0.066
cg11781328	chr19:4712833	DPP9	0.571	0.004
cg07317566	chr19:4713160	DPP9	0.503	0.008
cg07469449	chr19:4715647	DPP9	0.905	0.001
cg22802746	chr19:4717348	DPP9	0.895	0.000
cg07582623	chr19:4718742	DPP9	0.529	0.002
cg22561727	chr19:4719848	DPP9	0.463	0.003
cg08361380	chr19:4720405	DPP9	0.011	0.024
cg18347423	chr19:4723614	DPP9	0.769	0.000
cg14964934	chr19:4723942	DPP9	0.052	0.001
cg00371891	chr19:4723959	DPP9	0.128	0.004
cg16723130	chr19:4723970	DPP9	0.491	0.002
cg24423897	chr19:4723980	DPP9	0.982	0.001
cg02362719	chr19:4724564	DPP9	0.182	0.002
cg04163916	chr19:4724653	DPP9	0.007	0.006
cg15404665	chr19:4724679	DPP9	0.990	0.000
cg07317664	chr19:4724991	DPP9	0.114	0.006
cg12739264	chr19:4725282	DPP9	0.897	0.002
cg18295261	chr14:55736570	FBXO34	0.501	0.004
cg22468235	chr14:55737358	FBXO34	0.149	0.004
cg04071872	chr14:55737596	FBXO34	0.589	0.010
cg18427465	chr14:55737629	FBXO34	0.070	0.034
cg03911395	chr14:55737812	FBXO34	0.067	0.004
cg18251245	chr14:55737854	FBXO34	0.194	0.003

cg21761232	chr14:55737906	FBXO34	0.632	0.001
cg10184292	chr14:55737941	FBXO34	0.200	0.002
cg00390969	chr14:55737947	FBXO34	0.156	0.003
cg08336832	chr14:55737949	FBXO34	0.938	0.000
cg08430068	chr14:55738118	FBXO34	0.026	0.001
cg03088104	chr14:55738121	FBXO34	0.016	0.001
cg10378898	chr14:55738124	FBXO34	0.002	0.001
cg14947789	chr14:55738283	FBXO34	0.277	0.001
cg16351352	chr14:55738545	FBXO34	0.183	0.002
cg11449689	chr14:55738743	FBXO34	0.760	0.000
cg07017275	chr14:55738748	FBXO34	0.571	0.001
cg18846554	chr14:55738783	FBXO34	0.059	0.001
cg03362590	chr14:55738839	FBXO34	0.501	0.001
cg15990800	chr14:55738852	FBXO34	0.010	0.005
cg20664017	chr14:55738888	FBXO34	0.983	0.000
cg00043265	chr14:55738961	FBXO34	0.770	0.000
cg02577881	chr14:55739068	FBXO34	0.053	0.008
cg12572748	chr14:55739560	FBXO34	0.037	0.021
cg19885224	chr14:55740146	FBXO34	0.014	0.041
cg07085167	chr14:55740496	FBXO34	0.814	0.001
cg12786198	chr14:55741714	FBXO34	0.008	0.024
cg07001857	chr14:55745250	FBXO34	0.099	0.012
cg07489121	chr14:55747523	FBXO34	0.502	0.004
cg17984612	chr14:55753104	FBXO34	0.328	0.004
cg17290573	chr14:55753374	FBXO34	0.072	0.025
cg14053764	chr14:55761432	FBXO34	< 0.001	0.093
cg05890727	chr14:55764647	FBXO34	< 0.001	0.128
cg01518344	chr14:55766026	FBXO34	0.318	0.003
cg03487305	chr14:55767032	FBXO34	0.131	0.007
cg10940698	chr14:55767824	FBXO34	0.889	0.000
cg04853218	chr14:55769688	FBXO34	0.007	0.030
cg21709263	chr14:55784552	FBXO34	0.021	0.012
cg08139688	chr14:55793634	FBXO34	0.020	0.016
cg04971161	chr14:55794598	FBXO34	0.012	0.020
cg12034641	chr14:55799643	FBXO34	< 0.001	0.088
cg00703299	chr14:55800645	FBXO34	0.728	0.003
cg04236980	chr14:55800924	FBXO34	< 0.001	0.079
cg20068747	chr14:55804636	FBXO34	< 0.001	0.115
cg12242949	chr14:55806966	FBXO34	0.004	0.024
cg14897683	chr14:55807184	FBXO34	0.971	0.000
cg25483140	chr14:55807333	FBXO34	0.297	0.004
cg01565905	chr14:55815940	FBXO34	0.001	0.054
cg23234828	chr14:55818341	FBXO34	0.475	0.008
cg23236908	chr14:55819305	FBXO34	0.011	0.010
cg07529087	chr15:91410508	FURIN	0.003	0.016
cg11117530	chr15:91410755	FURIN	0.925	0.000

cg21792018	chr15:91410847	FURIN	0.931	0.000
cg24958468	chr15:91411462	FURIN	0.029	0.009
cg23208881	chr15:91411495	FURIN	0.993	0.000
cg02864248	chr15:91411838	FURIN	0.199	0.007
cg26377677	chr15:91412118	FURIN	0.859	0.002
cg19348484	chr15:91413236	FURIN	< 0.001	0.071
cg07381960	chr15:91413642	FURIN	0.011	0.014
cg13083201	chr15:91413783	FURIN	0.069	0.009
cg12539442	chr15:91414030	FURIN	0.466	0.006
cg24387797	chr15:91414270	FURIN	< 0.001	0.021
cg11582017	chr15:91414499	FURIN	0.157	0.004
cg24191278	chr15:91414591	FURIN	0.884	0.000
cg12297162	chr15:91414593	FURIN	0.171	0.002
cg24483306	chr15:91414797	FURIN	0.333	0.002
cg24259688	chr15:91414844	FURIN	0.743	0.002
cg01930518	chr15:91414850	FURIN	0.572	0.007
cg18274515	chr15:91415532	FURIN	0.525	0.001
cg04791659	chr15:91415812	FURIN	0.025	0.024
cg05466844	chr15:91415964	FURIN	0.933	0.002
cg23662846	chr15:91416006	FURIN	0.013	0.033
cg07162861	chr15:91416008	FURIN	0.266	0.031
cg12722937	chr15:91416047	FURIN	0.255	0.008
cg10698959	chr15:91416060	FURIN	0.436	0.005
cg00207731	chr15:91416118	FURIN	0.006	0.035
cg17019377	chr15:91417710	FURIN	0.003	0.051
cg13821613	chr15:91418757	FURIN	0.775	0.002
cg05469396	chr15:91419421	FURIN	0.207	0.005
cg22145651	chr15:91419540	FURIN	0.721	0.002
cg07281959	chr15:91421381	FURIN	0.884	0.000
cg25614263	chr15:91421585	FURIN	0.035	0.009
cg18926580	chr15:91422143	FURIN	0.983	0.000
cg03177204	chr15:91422169	FURIN	0.095	0.013
cg27503675	chr15:91422702	FURIN	0.491	0.003
cg05958346	chr15:91422794	FURIN	0.672	0.001
cg08372296	chr15:91422917	FURIN	0.846	0.002
cg19780300	chr15:91422948	FURIN	0.769	0.002
cg17396638	chr15:91423337	FURIN	0.923	0.006
cg25242556	chr15:91423375	FURIN	0.355	0.001
cg12075901	chr15:91423415	FURIN	0.897	0.001
cg25639557	chr15:91423574	FURIN	0.579	0.002
cg00758797	chr15:91424646	FURIN	0.974	0.000
cg09112160	chr15:91424658	FURIN	0.664	0.002
cg14231966	chr15:91424718	FURIN	0.926	0.001
cg05122887	chr15:91424836	FURIN	0.683	0.002
cg12015851	chr15:91425292	FURIN	0.078	0.014
cg16992008	chr3:45961867	FYCO1	0.739	0.002

cg20652158	chr3:45965449	FYCO1	0.856	0.008
cg19951818	chr3:45969823	FYCO1	0.816	0.001
cg03152139	chr3:45970474	FYCO1	0.080	0.012
ch.3.946759R	chr3:45979073	FYCO1	0.846	0.000
cg05873635	chr3:45980975	FYCO1	0.549	0.003
cg16320329	chr3:45981161	FYCO1	0.005	0.034
cg27123613	chr3:45982438	FYCO1	0.368	0.004
cg17542594	chr3:45990547	FYCO1	0.299	0.003
cg06187522	chr3:46000100	FYCO1	< 0.001	0.038
cg07343122	chr3:46000900	FYCO1	0.990	0.000
cg24899967	chr3:46003807	FYCO1	< 0.001	0.042
cg21080682	chr3:46008371	FYCO1	0.520	0.003
cg08257567	chr3:46009762	FYCO1	0.189	0.005
cg13941085	chr3:46010476	FYCO1	0.005	0.010
cg08042841	chr3:46014624	FYCO1	0.458	0.003
cg07949967	chr3:46017480	FYCO1	0.188	0.011
cg16286502	chr3:46020045	FYCO1	0.006	0.046
cg19375371	chr3:46021163	FYCO1	0.004	0.057
cg09251548	chr3:46022682	FYCO1	0.608	0.002
ch.3.948191R	chr3:46024851	FYCO1	0.084	0.003
cg09923715	chr3:46027565	FYCO1	0.940	0.000
cg03695289	chr3:46027639	FYCO1	0.769	0.001
cg04732163	chr3:46029147	FYCO1	0.053	0.009
cg11211486	chr3:46033800	FYCO1	0.532	0.011
cg04415535	chr3:46034485	FYCO1	0.004	0.062
cg18682028	chr3:46035439	FYCO1	< 0.001	0.066
cg24208206	chr3:46036571	FYCO1	0.421	0.011
cg04799181	chr3:46037014	FYCO1	0.272	0.003
cg15068835	chr3:46037408	FYCO1	0.081	0.003
cg12920938	chr3:46037457	FYCO1	0.225	0.002
cg05573133	chr3:46037470	FYCO1	0.053	0.002
cg03245407	chr3:46037472	FYCO1	0.364	0.002
cg04619627	chr3:46037496	FYCO1	0.182	0.011
cg12609415	chr3:46037548	FYCO1	0.123	0.023
cg16042668	chr3:46037592	FYCO1	0.546	0.018
cg24919972	chr3:46037608	FYCO1	0.326	0.011
cg06585734	chr3:46037942	FYCO1	0.322	0.012
cg12062507	chr3:46037976	FYCO1	< 0.001	0.057
cg00397283	chr19:10444479	ICAM3	0.822	0.002
cg07976603	chr19:10444506	ICAM3	0.921	0.004
cg14667838	chr19:10445908	ICAM3	0.497	0.006
cg13981319	chr19:10446556	ICAM3	0.990	0.000
cg05323251	chr19:10449942	ICAM3	0.841	0.007
cg14145194	chr19:10450022	ICAM3	0.391	0.009
cg23671824	chr19:10450323	ICAM3	0.804	0.001
cg13021446	chr19:10450346	ICAM3	0.002	0.002

cg00911290	chr19:10450361	ICAM3	0.614	0.001
cg06855803	chr19:10450364	ICAM3	0.056	0.005
cg16659963	chr19:10450375	ICAM3	0.166	0.004
cg18054725	chr19:10450472	ICAM3	0.118	0.004
cg26972937	chr19:10450862	ICAM3	0.930	0.001
cg08782606	chr19:10450872	ICAM3	0.128	0.019
cg21901386	chr19:10451423	ICAM3	0.944	0.000
cg05202822	chr19:10451653	ICAM3	0.927	0.000
cg03279442	chr2:163150027	IFIH1	0.007	0.090
cg24105617	chr2:163155125	IFIH1	0.734	0.009
cg07181667	chr2:163157840	IFIH1	0.102	0.006
cg03463287	chr2:163163925	IFIH1	0.035	0.013
cg08888522	chr2:163172908	IFIH1	0.503	0.006
cg00531628	chr2:163174891	IFIH1	0.050	0.003
cg07348311	chr2:163174896	IFIH1	0.008	0.004
cg19533050	chr2:163175044	IFIH1	0.002	0.002
cg24885055	chr2:163175046	IFIH1	0.575	0.001
cg06347928	chr2:163175126	IFIH1	0.215	0.004
cg23553385	chr2:163175143	IFIH1	0.730	0.001
cg18340987	chr2:163175241	IFIH1	0.388	0.001
cg02029939	chr2:163175255	IFIH1	0.001	0.004
cg02276086	chr2:163175335	IFIH1	0.164	0.004
cg21810130	chr2:163175381	IFIH1	0.156	0.002
cg09755666	chr2:163175400	IFIH1	0.472	0.003
cg16971745	chr2:163175403	IFIH1	0.783	0.001
cg23280745	chr2:163175536	IFIH1	0.734	0.001
cg19965693	chr2:163175743	IFIH1	0.222	0.021
cg21060789	chr2:163176242	IFIH1	0.350	0.004
cg26974214	chr10:91151885	IFIT1	0.002	0.021
cg16395953	chr10:91152122	IFIT1	< 0.001	0.008
cg11748577	chr10:91152280	IFIT1	< 0.001	0.011
cg15019617	chr10:91152447	IFIT1	0.208	0.002
cg05552874	chr10:91153143	IFIT1	0.230	0.027
cg07508980	chr10:91164540	IFIT1	0.483	0.004
cg06920054	chr21:34696533	IFNAR1	0.086	0.003
cg23202109	chr21:34696546	IFNAR1	0.042	0.004
cg14069320	chr21:34696571	IFNAR1	0.053	0.002
cg11758840	chr21:34696588	IFNAR1	0.011	0.007
cg04842828	chr21:34696676	IFNAR1	0.510	0.001
cg14352951	chr21:34696688	IFNAR1	0.575	0.001
cg15382669	chr21:34697036	IFNAR1	0.032	0.006
cg00207965	chr21:34697220	IFNAR1	0.072	0.009
cg15509148	chr21:34697369	IFNAR1	0.712	0.001
cg03866569	chr21:34697528	IFNAR1	0.339	0.006
cg09885409	chr21:34697563	IFNAR1	0.023	0.008
cg06121323	chr21:34697899	IFNAR1	0.538	0.002

cg20274167	chr21:34699063	IFNAR1	0.010	0.078
cg14911223	chr21:34700152	IFNAR1	0.238	0.009
cg12016862	chr21:34705183	IFNAR1	0.030	0.016
cg12078491	chr21:34718964	IFNAR1	< 0.001	0.038
cg22029234	chr21:34720480	IFNAR1	0.037	0.005
cg00622702	chr21:34727950	IFNAR1	0.873	0.002
cg04049819	chr21:34729667	IFNAR1	0.103	0.014
cg05667472	chr21:34730291	IFNAR1	0.701	0.002
cg20000468	chr21:34601686	IFNAR2	0.096	0.009
cg01475000	chr21:34601704	IFNAR2	0.013	0.028
cg12269436	chr21:34601738	IFNAR2	0.929	0.002
cg17977197	chr21:34601762	IFNAR2	0.733	0.004
cg17725807	chr21:34601817	IFNAR2	0.088	0.023
cg22454603	chr21:34602027	IFNAR2	0.775	0.001
cg21980821	chr21:34602030	IFNAR2	0.212	0.003
cg20215695	chr21:34602070	IFNAR2	0.918	0.000
cg10309613	chr21:34602084	IFNAR2	0.675	0.001
cg05770313	chr21:34602116	IFNAR2	0.578	0.001
cg00937568	chr21:34602128	IFNAR2	0.506	0.002
cg21355791	chr21:34602157	IFNAR2	0.075	0.002
cg26110456	chr21:34602354	IFNAR2	0.110	0.001
cg18656581	chr21:34602557	IFNAR2	0.075	0.007
cg02431704	chr21:34602713	IFNAR2	0.008	0.007
cg06665941	chr21:34602869	IFNAR2	0.067	0.005
cg24768245	chr21:34602948	IFNAR2	0.035	0.009
cg13208562	chr21:34603264	IFNAR2	0.004	0.005
cg22030766	chr21:34608899	IFNAR2	0.638	0.002
cg24851988	chr21:34617885	IFNAR2	0.548	0.004
cg27342941	chr21:34619110	IFNAR2	0.470	0.017
cg04857551	chr21:34619143	IFNAR2	0.056	0.007
cg06339338	chr21:34620140	IFNAR2	0.084	0.079
cg00288436	chr21:34625037	IFNAR2	0.462	0.006
cg12122356	chr21:34632557	IFNAR2	0.705	0.009
cg24949115	chr21:34634409	IFNAR2	0.292	0.011
cg17748222	chr21:34634995	IFNAR2	0.309	0.003
cg16134430	chr21:34636155	IFNAR2	0.683	0.002
cg01467417	chr2:113874068	IL1RN	0.995	0.001
cg10816993	chr2:113874415	IL1RN	0.052	0.042
cg00303311	chr2:113874822	IL1RN	0.772	0.001
cg03703171	chr2:113875226	IL1RN	0.928	0.001
cg06658391	chr2:113875233	IL1RN	< 0.001	0.018
cg11783497	chr2:113875292	IL1RN	0.074	0.010
cg14497465	chr2:113875377	IL1RN	< 0.001	0.025
cg17669033	chr2:113875512	IL1RN	0.240	0.007
cg10938446	chr2:113875723	IL1RN	0.310	0.002
cg18783495	chr2:113876804	IL1RN	0.004	0.018

cg23536855	chr2:113882233	IL1RN	0.004	0.025
cg04572943	chr2:113882976	IL1RN	0.178	0.028
cg00795815	chr2:113884024	IL1RN	0.055	0.024
cg25928199	chr2:113884376	IL1RN	0.099	0.015
cg23041410	chr2:113884447	IL1RN	0.055	0.008
cg01991967	chr2:113884734	IL1RN	0.914	0.002
cg02543462	chr2:113885116	IL1RN	0.106	0.025
cg03989987	chr2:113885277	IL1RN	0.010	0.050
cg02377053	chr2:113885838	IL1RN	0.001	0.012
cg25265126	chr2:113891507	IL1RN	< 0.001	0.050
cg17067544	chr7:22765321	IL6	0.004	0.038
cg10140158	chr7:22765750	IL6	0.788	0.004
cg01770232	chr7:22766155	IL6	0.824	0.008
cg00087425	chr7:22766829	IL6	0.076	0.012
cg15703690	chr7:22766992	IL6	< 0.001	0.030
cg20509117	chr7:22767314	IL6	0.314	0.033
cg13104385	chr7:22767384	IL6	0.300	0.027
cg05265849	chr7:22767390	IL6	0.417	0.030
cg07998387	chr7:22767571	IL6	0.008	0.031
cg02335517	chr7:22768438	IL6	0.073	0.010
cg03172226	chr19:50162898	IRF3	0.112	0.021
cg06444294	chr19:50164473	IRF3	0.944	0.001
cg18517766	chr19:50164488	IRF3	0.199	0.006
cg27662875	chr19:50165232	IRF3	0.866	0.001
cg16253779	chr19:50165958	IRF3	0.983	0.000
cg17540624	chr19:50167792	IRF3	0.161	0.002
cg23029494	chr19:50167926	IRF3	0.174	0.002
cg11519876	chr19:50168004	IRF3	0.307	0.001
cg12107692	chr19:50168026	IRF3	0.725	0.000
cg21035241	chr19:50168129	IRF3	0.848	0.001
cg13102229	chr19:50168131	IRF3	0.258	0.002
cg25264707	chr19:50168190	IRF3	0.023	0.003
cg05182906	chr19:50168328	IRF3	0.683	0.001
cg22136800	chr19:50168459	IRF3	0.218	0.003
cg09250473	chr19:50168907	IRF3	0.108	0.006
cg07333040	chr19:50168911	IRF3	0.630	0.003
cg07215350	chr19:50169079	IRF3	0.760	0.000
cg23891596	chr19:50169132	IRF3	0.952	0.002
cg06490385	chr19:50169153	IRF3	0.645	0.000
cg22761099	chr19:50169171	IRF3	0.526	0.001
cg09712606	chr19:50169182	IRF3	0.558	0.001
cg05495029	chr19:50169187	IRF3	0.814	0.001
cg06639101	chr19:50169228	IRF3	0.257	0.001
cg18477816	chr11:612588	IRF7	0.348	0.005
cg03755158	chr11:612680	IRF7	0.024	0.012
cg27271532	chr11:612762	IRF7	0.004	0.021

cg05309505	chr11:612837	IRF7	0.806	0.001
cg20989454	chr11:613478	IRF7	0.852	0.001
cg16486109	chr11:613632	IRF7	0.059	0.042
cg17114584	chr11:613792	IRF7	0.137	0.021
cg22199238	chr11:613857	IRF7	0.092	0.021
cg23540139	chr11:614521	IRF7	0.103	0.013
cg08926253	chr11:614761	IRF7	0.203	0.018
cg22016995	chr11:614787	IRF7	0.627	0.001
cg12037516	chr11:614954	IRF7	0.852	0.001
cg16541031	chr11:615519	IRF7	0.938	0.001
cg13406746	chr11:615583	IRF7	0.930	0.000
cg05460606	chr11:615635	IRF7	0.346	0.001
cg09396517	chr11:615642	IRF7	0.957	0.001
cg06770435	chr11:615716	IRF7	0.057	0.001
cg18956798	chr11:615721	IRF7	0.128	0.001
cg18131537	chr11:615732	IRF7	0.293	0.001
cg24064264	chr11:615945	IRF7	0.930	0.000
cg07652350	chr11:616009	IRF7	0.288	0.001
cg22559350	chr11:616011	IRF7	0.119	0.001
cg13145591	chr11:616038	IRF7	0.548	0.001
cg13378284	chr11:616089	IRF7	0.525	0.002
cg18971819	chr11:616112	IRF7	0.037	0.008
cg09703963	chr11:616879	IRF7	0.984	0.002
cg00645579	chr11:617140	IRF7	< 0.001	0.088
cg01842473	chr11:617407	IRF7	0.125	0.002
cg01714160	chr11:617467	IRF7	0.626	0.000
cg15780465	chr11:617476	IRF7	0.692	0.001
cg18769584	chr3:45866619	LZTFL1	0.826	0.001
cg00482389	chr3:45867935	LZTFL1	< 0.001	0.078
cg01865626	chr3:45874300	LZTFL1	0.774	0.001
cg02909320	chr3:45879990	LZTFL1	0.727	0.001
cg03032607	chr3:45882686	LZTFL1	0.519	0.009
cg06799455	chr3:45882924	LZTFL1	0.036	0.007
cg08661012	chr3:45883249	LZTFL1	0.341	0.003
cg17500645	chr3:45883368	LZTFL1	0.011	0.002
cg18713168	chr3:45883381	LZTFL1	0.724	0.000
cg14999852	chr3:45883529	LZTFL1	0.277	0.001
cg12857957	chr3:45883621	LZTFL1	0.977	0.001
cg05485520	chr3:45883628	LZTFL1	0.432	0.003
cg08186124	chr3:45883676	LZTFL1	0.915	0.000
cg18770816	chr3:45883693	LZTFL1	0.069	0.002
cg21878420	chr3:45883716	LZTFL1	0.487	0.002
cg24259629	chr3:45883796	LZTFL1	0.076	0.002
cg01693650	chr3:45883855	LZTFL1	0.286	0.002
cg15061025	chr3:45883859	LZTFL1	< 0.001	0.025
cg04817148	chr3:45884404	LZTFL1	0.068	0.004

cg13426237	chr3:45884877	LZTFL1	0.800	0.001
cg19432791	chr3:45894556	LZTFL1	0.208	0.018
cg00634029	chr3:45900436	LZTFL1	0.974	0.012
cg22850860	chr3:45902663	LZTFL1	0.014	0.021
cg01884851	chr3:45906498	LZTFL1	< 0.001	0.113
cg06730002	chr3:45906856	LZTFL1	0.003	0.075
cg09709426	chr3:45911521	LZTFL1	0.830	0.002
cg23890383	chr3:45913499	LZTFL1	< 0.001	0.026
cg10650214	chr3:45914188	LZTFL1	0.166	0.015
cg03986594	chr3:45922512	LZTFL1	< 0.001	0.061
cg09355011	chr3:45926450	LZTFL1	< 0.001	0.047
cg13312303	chr3:45951308	LZTFL1	0.227	0.006
cg25434241	chr3:45953557	LZTFL1	< 0.001	0.022
cg03297297	chr3:45957266	LZTFL1	< 0.001	0.071
cg25897930	chr3:45957319	LZTFL1	0.005	0.048
cg10418021	chr3:45957347	LZTFL1	< 0.001	0.079
cg12924105	chr3:45957730	LZTFL1	< 0.001	0.104
cg12988275	chr3:45958440	LZTFL1	0.444	0.002
cg00728099	chr3:45958469	LZTFL1	0.203	0.007
cg20933330	chr3:45958552	LZTFL1	0.004	0.018
cg15634935	chr13:103347028	METTL21C	0.609	0.003
cg10371253	chr13:103347386	METTL21C	0.701	0.001
cg00530029	chr13:103348317	METTL21C	0.067	0.020
cg13407144	chr10:33468337	NRP1	0.580	0.001
ch.10.844982F	chr10:33469591	NRP1	0.033	0.002
cg22620273	chr10:33474766	NRP1	0.756	0.001
cg25179876	chr10:33483109	NRP1	0.035	0.048
cg27039218	chr10:33485389	NRP1	0.381	0.004
cg09603237	chr10:33486310	NRP1	0.948	0.001
cg12045634	chr10:33491867	NRP1	0.262	0.008
cg02543939	chr10:33492000	NRP1	0.082	0.005
cg16087345	chr10:33493031	NRP1	0.347	0.005
cg04287203	chr10:33496604	NRP1	0.231	0.004
cg23444399	chr10:33499848	NRP1	0.057	0.008
cg13114217	chr10:33499997	NRP1	0.925	0.000
cg12841561	chr10:33500134	NRP1	0.588	0.006
cg26295541	chr10:33500231	NRP1	0.094	0.006
ch.10.845914F	chr10:33501874	NRP1	0.239	0.005
cg00717280	chr10:33502502	NRP1	0.539	0.002
cg14982967	chr10:33502525	NRP1	0.257	0.004
cg26086245	chr10:33502540	NRP1	0.689	0.002
cg06512724	chr10:33502889	NRP1	0.061	0.007
cg05504719	chr10:33503366	NRP1	0.002	0.028
cg01874729	chr10:33503472	NRP1	0.036	0.006
cg23912035	chr10:33505747	NRP1	0.886	0.001
cg20687152	chr10:33507026	NRP1	0.095	0.005

cg09277467	chr10:33512002	NRP1	0.007	0.019
cg06338245	chr10:33515103	NRP1	0.403	0.003
cg24638819	chr10:33515115	NRP1	0.880	0.000
cg04721557	chr10:33515198	NRP1	0.525	0.001
cg14219813	chr10:33515236	NRP1	0.643	0.004
cg15809806	chr10:33518295	NRP1	0.009	0.008
cg08358427	chr10:33522703	NRP1	0.604	0.004
cg03442322	chr10:33524393	NRP1	0.094	0.007
cg25763127	chr10:33527263	NRP1	0.519	0.002
cg20701074	chr10:33532360	NRP1	0.082	0.007
cg00346755	chr10:33534164	NRP1	0.610	0.004
cg02977812	chr10:33537894	NRP1	< 0.001	0.003
cg21379625	chr10:33538715	NRP1	< 0.001	0.051
cg09030553	chr10:33538770	NRP1	0.981	0.000
cg15391489	chr10:33539640	NRP1	0.330	0.004
cg07913781	chr10:33541110	NRP1	0.097	0.006
cg03602698	chr10:33542985	NRP1	0.733	0.001
cg17470096	chr10:33546865	NRP1	0.718	0.001
cg08839819	chr10:33547946	NRP1	0.493	0.002
cg12622378	chr10:33552748	NRP1	0.227	0.004
cg09084320	chr10:33553435	NRP1	0.821	0.001
cg10419493	chr10:33561323	NRP1	0.755	0.001
cg24892069	chr10:33562205	NRP1	0.418	0.026
cg17812637	chr10:33562403	NRP1	0.678	0.014
cg11345700	chr10:33565458	NRP1	0.081	0.005
cg03473377	chr10:33568029	NRP1	0.702	0.005
cg16131207	chr10:33575017	NRP1	0.029	0.020
cg17706662	chr10:33576866	NRP1	0.031	0.016
cg20330217	chr10:33578803	NRP1	0.637	0.002
cg25713793	chr10:33590732	NRP1	0.127	0.010
cg03935991	chr10:33592746	NRP1	0.157	0.008
cg05989059	chr10:33594454	NRP1	0.132	0.007
cg14965866	chr10:33596300	NRP1	0.060	0.011
cg22279720	chr10:33603872	NRP1	< 0.001	0.057
cg10312802	chr10:33604657	NRP1	0.119	0.005
cg21998542	chr10:33605101	NRP1	0.832	0.005
cg12054892	chr10:33607723	NRP1	0.072	0.030
cg05293915	chr10:33608477	NRP1	0.995	0.000
cg24517944	chr10:33608887	NRP1	0.073	0.006
cg19131541	chr10:33608914	NRP1	0.719	0.006
cg17425818	chr10:33612892	NRP1	0.004	0.057
cg06898048	chr10:33614690	NRP1	0.015	0.025
cg06859734	chr10:33617564	NRP1	0.588	0.005
cg25733480	chr10:33619701	NRP1	0.073	0.004
cg13090418	chr10:33621421	NRP1	0.571	0.016
cg27270412	chr10:33622332	NRP1	0.942	0.001

cg07233135	chr10:33623121	NRP1	0.295	0.003
cg20827580	chr10:33623389	NRP1	0.627	0.001
cg23313663	chr10:33623410	NRP1	0.593	0.001
cg17026287	chr10:33623747	NRP1	0.536	0.000
cg13779055	chr10:33623762	NRP1	0.845	0.000
cg23198568	chr10:33623905	NRP1	0.723	0.001
cg09009410	chr10:33624100	NRP1	0.576	0.001
cg13186597	chr10:33624123	NRP1	0.503	0.002
cg00826728	chr10:33624131	NRP1	0.276	0.003
cg12870705	chr10:33624623	NRP1	0.035	0.002
cg09365847	chr10:33624868	NRP1	0.977	0.000
cg24892156	chr10:33625310	NRP1	0.690	0.006
cg09151598	chr12:113343409	OAS1	0.211	0.010
cg17445535	chr12:113343413	OAS1	0.537	0.003
cg25668626	chr12:113343603	OAS1	0.549	0.003
cg24189072	chr12:113344374	OAS1	0.119	0.003
cg13483603	chr12:113344754	OAS1	0.027	0.002
cg19789466	chr12:113344923	OAS1	0.761	0.015
cg04951822	chr12:113345598	OAS1	0.836	0.001
cg06168856	chr12:113346982	OAS1	< 0.001	0.068
cg21197031	chr12:113354037	OAS1	0.811	0.000
cg04708790	chr12:113355675	OAS1	0.756	0.005
cg19609748	chr12:113356835	OAS1	0.982	0.000
cg12877361	chr12:113356935	OAS1	0.544	0.009
cg21730677	chr12:113357028	OAS1	0.985	0.000
cg18217049	chr12:113357226	OAS1	0.725	0.002
cg20726456	chr12:113415459	OAS2	0.009	0.019
cg19371652	chr12:113415883	OAS2	0.833	0.002
cg11601443	chr12:113415930	OAS2	0.092	0.013
cg04880620	chr12:113415945	OAS2	0.469	0.007
cg03996150	chr12:113416055	OAS2	0.859	0.001
cg12560128	chr12:113416061	OAS2	0.588	0.001
cg11318133	chr12:113416268	OAS2	0.107	0.004
cg00175901	chr12:113416290	OAS2	0.003	0.003
cg00085448	chr12:113416442	OAS2	0.908	0.002
cg20870559	chr12:113416518	OAS2	0.798	0.001
cg16399664	chr12:113417284	OAS2	0.748	0.001
cg16366376	chr12:113425484	OAS2	0.486	0.002
cg06255132	chr12:113426510	OAS2	0.986	0.000
cg12247536	chr12:113428107	OAS2	0.899	0.000
cg03320783	chr12:113430736	OAS2	0.553	0.017
cg06217905	chr12:113435438	OAS2	0.744	0.002
cg16382882	chr12:113435923	OAS2	0.164	0.004
cg25682386	chr12:113435980	OAS2	0.713	0.002
cg06154315	chr12:113436953	OAS2	0.637	0.011
cg24531077	chr12:113440283	OAS2	0.773	0.007

cg20800221	chr12:113443790	OAS2	0.603	0.003
cg03210276	chr12:113445157	OAS2	0.009	0.011
cg07253769	chr12:113447342	OAS2	0.571	0.008
cg03240232	chr12:113448327	OAS2	0.034	0.020
cg08147692	chr12:113375402	OAS3	0.299	0.003
cg22447989	chr12:113375561	OAS3	0.026	0.046
cg22260958	chr12:113375880	OAS3	0.317	0.010
cg25800166	chr12:113375896	OAS3	0.805	0.004
cg24515746	chr12:113375961	OAS3	0.015	0.016
cg25319449	chr12:113376135	OAS3	0.856	0.000
cg01128221	chr12:113376260	OAS3	0.950	0.001
cg22026360	chr12:113376264	OAS3	0.448	0.001
cg18120998	chr12:113376286	OAS3	0.937	0.000
cg16458507	chr12:113376293	OAS3	0.092	0.005
cg03331867	chr12:113376492	OAS3	0.444	0.003
cg20102336	chr12:113376681	OAS3	0.637	0.002
cg09167772	chr12:113377933	OAS3	0.612	0.001
cg27066543	chr12:113381877	OAS3	0.443	0.002
cg24948564	chr12:113384576	OAS3	0.132	0.009
cg25297038	chr12:113386963	OAS3	0.867	0.001
cg11236111	chr12:113402109	OAS3	0.356	0.002
cg11229468	chr12:113405865	OAS3	0.781	0.001
cg22044401	chr12:113405926	OAS3	0.469	0.002
cg18448515	chr12:113406128	OAS3	0.183	0.004
cg02066269	chr5:76505273	PDE8B	0.184	0.008
cg08967003	chr5:76505571	PDE8B	0.809	0.000
cg23942789	chr5:76505840	PDE8B	0.010	0.014
cg06339657	chr5:76505932	PDE8B	0.599	0.005
cg25373100	chr5:76506484	PDE8B	0.208	0.001
cg02329935	chr5:76506492	PDE8B	0.119	0.001
cg13554086	chr5:76507100	PDE8B	0.824	0.000
cg06025835	chr5:76507298	PDE8B	0.034	0.006
cg16461538	chr5:76507484	PDE8B	0.095	0.004
cg22203582	chr5:76507680	PDE8B	< 0.001	0.011
cg13077359	chr5:76512781	PDE8B	0.201	0.005
cg01676318	chr5:76514167	PDE8B	0.061	0.012
cg21060420	chr5:76520875	PDE8B	0.613	0.005
cg16812042	chr5:76527030	PDE8B	0.001	0.023
cg14878361	chr5:76527359	PDE8B	< 0.001	0.033
cg15788355	chr5:76535026	PDE8B	< 0.001	0.054
cg08189790	chr5:76547849	PDE8B	0.231	0.006
cg19829058	chr5:76549927	PDE8B	0.039	0.013
cg11062417	chr5:76578232	PDE8B	0.054	0.011
cg19436353	chr5:76586024	PDE8B	0.580	0.002
cg13003600	chr5:76586623	PDE8B	0.107	0.014
cg15375344	chr5:76598038	PDE8B	0.776	0.002

cg08281791	chr5:76607845	PDE8B	0.845	0.002
cg10292028	chr5:76621420	PDE8B	0.735	0.001
cg26964901	chr5:76630355	PDE8B	0.005	0.013
cg04307776	chr5:76652757	PDE8B	0.439	0.015
cg18631313	chr5:76653473	PDE8B	0.541	0.003
cg12559197	chr5:76654783	PDE8B	0.010	0.062
cg17430199	chr5:76663105	PDE8B	0.695	0.009
cg20872940	chr5:76663728	PDE8B	< 0.001	0.051
cg16334075	chr5:76670503	PDE8B	0.017	0.010
cg26357116	chr5:76675528	PDE8B	0.015	0.049
cg10189799	chr5:76686823	PDE8B	0.998	0.002
cg11460929	chr5:76690047	PDE8B	0.974	0.000
cg19701224	chr5:76704767	PDE8B	0.718	0.001
cg03195910	chr5:76704995	PDE8B	0.337	0.009
cg01035629	chr5:76705019	PDE8B	0.223	0.006
cg22011212	chr5:76706050	PDE8B	0.717	0.010
cg13714288	chr5:76713158	PDE8B	0.063	0.012
cg03695340	chr5:76721970	PDE8B	0.401	0.003
cg05010493	chr5:76722964	PDE8B	0.628	0.002
ch.5.1450929R	chr5:76723053	PDE8B	0.509	0.003
cg15219523	chr2:209129921	PIKFYVE	0.562	0.011
cg03701609	chr2:209129993	PIKFYVE	0.258	0.015
cg02669268	chr2:209130300	PIKFYVE	0.799	0.001
cg09509267	chr2:209130442	PIKFYVE	0.858	0.010
cg03378289	chr2:209130693	PIKFYVE	0.437	0.001
cg01046174	chr2:209130700	PIKFYVE	0.866	0.000
cg00707300	chr2:209130779	PIKFYVE	0.707	0.001
cg24830471	chr2:209130825	PIKFYVE	0.830	0.001
cg24987806	chr2:209130885	PIKFYVE	0.623	0.003
cg15399923	chr2:209130889	PIKFYVE	0.954	0.000
cg09615745	chr2:209130978	PIKFYVE	0.071	0.001
cg25064853	chr2:209131103	PIKFYVE	0.394	0.002
cg05268564	chr2:209131274	PIKFYVE	0.153	0.003
cg02241586	chr2:209131508	PIKFYVE	0.897	0.000
cg19351166	chr2:209133632	PIKFYVE	0.630	0.012
cg11969526	chr2:209145835	PIKFYVE	0.044	0.008
cg24476365	chr2:209163171	PIKFYVE	0.062	0.038
cg03065139	chr2:209169833	PIKFYVE	0.365	0.008
cg18100085	chr2:209176730	PIKFYVE	< 0.001	0.022
cg10725403	chr2:209180074	PIKFYVE	0.851	0.003
cg03671627	chr2:209185667	PIKFYVE	0.856	0.006
cg24468036	chr2:209197652	PIKFYVE	0.030	0.016
cg08621874	chr2:209198208	PIKFYVE	0.518	0.002
cg00273392	chr2:209198437	PIKFYVE	0.005	0.011
cg27443844	chr2:209206217	PIKFYVE	0.802	0.001
cg24539923	chr7:100769903	SERPINE1	0.956	0.000

cg19722814	chr7:100769933	SERPINE1	0.003	0.010
cg25826546	chr7:100770060	SERPINE1	0.179	0.004
cg20438404	chr7:100770192	SERPINE1	0.708	0.001
cg08506775	chr7:100770286	SERPINE1	0.003	0.013
cg02273392	chr7:100770414	SERPINE1	0.466	0.002
cg15874872	chr7:100770434	SERPINE1	0.075	0.005
cg20583316	chr7:100770476	SERPINE1	0.999	0.000
cg01971264	chr7:100773079	SERPINE1	0.061	0.012
cg08792542	chr7:100773718	SERPINE1	0.356	0.005
cg12584355	chr7:100773851	SERPINE1	0.170	0.006
cg11353706	chr7:100774810	SERPINE1	0.568	0.006
cg01975495	chr7:100779017	SERPINE1	0.063	0.024
cg02704552	chr7:100781178	SERPINE1	0.445	0.008
cg04746914	chr3:45797767	SLC6A20	0.223	0.010
cg01451880	chr3:45801044	SLC6A20	0.061	0.003
cg09251680	chr3:45801144	SLC6A20	0.889	0.000
cg07430193	chr3:45801965	SLC6A20	0.259	0.012
cg02414266	chr3:45802101	SLC6A20	0.500	0.010
cg17323778	chr3:45803563	SLC6A20	0.280	0.006
cg25727101	chr3:45804291	SLC6A20	0.011	0.011
cg01147011	chr3:45807290	SLC6A20	0.016	0.007
cg05403822	chr3:45807402	SLC6A20	0.003	0.030
cg26339753	chr3:45807440	SLC6A20	0.088	0.005
cg01311379	chr3:45807578	SLC6A20	0.206	0.012
cg04709528	chr3:45807687	SLC6A20	0.166	0.010
cg25344330	chr3:45807712	SLC6A20	0.647	0.001
cg26208450	chr3:45813982	SLC6A20	0.969	0.000
cg07606990	chr3:45814035	SLC6A20	0.072	0.009
cg26643209	chr3:45818166	SLC6A20	0.584	0.005
cg24261921	chr3:45821484	SLC6A20	0.462	0.004
cg27269492	chr3:45821536	SLC6A20	0.630	0.005
cg00256353	chr3:45823127	SLC6A20	0.188	0.011
cg07288822	chr3:45825528	SLC6A20	0.133	0.004
cg15576918	chr3:45834369	SLC6A20	< 0.001	0.034
cg04491419	chr3:45835519	SLC6A20	0.045	0.008
cg05461503	chr3:45837170	SLC6A20	0.062	0.025
cg24940967	chr3:45837197	SLC6A20	0.646	0.010
cg12359315	chr3:45837458	SLC6A20	0.379	0.002
cg20953187	chr3:45837898	SLC6A20	0.010	0.004
cg09700085	chr3:45838028	SLC6A20	0.013	0.018
cg04970117	chr3:45838037	SLC6A20	0.861	0.000
cg02716516	chr3:45838074	SLC6A20	0.318	0.001
cg20829379	chr3:45838083	SLC6A20	0.843	0.000
cg25924911	chr3:45838094	SLC6A20	0.509	0.001
cg22497313	chr3:45838198	SLC6A20	0.061	0.005
cg08826127	chr3:45838226	SLC6A20	0.988	0.000

cg16309260	chr3:45838338	SLC6A20	0.001	0.021
cg04012198	chr3:45838475	SLC6A20	0.218	0.014
cg17165824	chr3:45838670	SLC6A20	0.981	0.000
cg13719314	chr3:45839377	SLC6A20	0.014	0.024
cg12944551	chr2:191839759	STAT1	0.003	0.017
cg17486692	chr2:191839947	STAT1	0.922	0.001
cg22595870	chr2:191840398	STAT1	0.181	0.004
cg21683635	chr2:191841735	STAT1	0.240	0.005
cg00467636	chr2:191842259	STAT1	0.169	0.005
cg03496077	chr2:191842287	STAT1	0.129	0.005
cg08946713	chr2:191844998	STAT1	< 0.001	0.065
cg24308210	chr2:191848451	STAT1	0.172	0.003
cg00137918	chr2:191850909	STAT1	0.220	0.004
cg19881581	chr2:191859930	STAT1	0.349	0.003
cg10311754	chr2:191861148	STAT1	0.448	0.002
cg13444194	chr2:191872935	STAT1	0.826	0.002
cg07599830	chr2:191873753	STAT1	0.140	0.006
cg25714134	chr2:191873795	STAT1	0.303	0.003
cg16380614	chr2:191874140	STAT1	0.297	0.004
cg14951497	chr2:191875807	STAT1	0.002	0.039
cg00676801	chr2:191876673	STAT1	0.001	0.051
cg18761133	chr2:191878353	STAT1	0.504	0.006
cg26038976	chr2:191878357	STAT1	0.005	0.003
cg24997845	chr2:191878460	STAT1	0.102	0.004
cg01085225	chr2:191878686	STAT1	0.335	0.001
cg25856179	chr2:191878705	STAT1	0.036	0.001
cg11065262	chr2:191878861	STAT1	0.495	0.003
cg25579739	chr2:191878969	STAT1	0.085	0.001
cg11255154	chr2:191879104	STAT1	0.524	0.006
cg07052015	chr2:191879114	STAT1	0.728	0.001
cg00493400	chr2:191879127	STAT1	0.354	0.001
cg21968799	chr2:191879152	STAT1	0.889	0.000
cg19050596	chr2:191879164	STAT1	0.726	0.001
cg18766080	chr2:191879342	STAT1	0.906	0.002
cg22833204	chr2:191879510	STAT1	0.024	0.032
cg12159504	chr2:191879541	STAT1	0.980	0.003
cg07730886	chr2:191879796	STAT1	0.037	0.010
cg07780782	chr17:40465546	STAT3	< 0.001	0.041
cg11264678	chr17:40466210	STAT3	0.003	0.045
cg25330422	chr17:40467382	STAT3	0.024	0.042
cg04949579	chr17:40469283	STAT3	0.014	0.013
cg18852825	chr17:40469642	STAT3	0.043	0.017
cg06769931	chr17:40470564	STAT3	0.002	0.015
cg22128509	chr17:40474338	STAT3	0.934	0.001
cg26107598	chr17:40474351	STAT3	0.088	0.006
cg22197416	chr17:40474492	STAT3	0.390	0.005

cg02206695	chr17:40474743	STAT3	0.894	0.000
cg04439252	chr17:40475282	STAT3	0.042	0.021
cg08308989	chr17:40476724	STAT3	0.480	0.004
cg03647657	chr17:40477020	STAT3	0.688	0.002
cg00476906	chr17:40477266	STAT3	0.001	0.015
cg06903086	chr17:40481955	STAT3	0.923	0.001
cg03164379	chr17:40483184	STAT3	0.818	0.014
cg00508048	chr17:40485194	STAT3	0.004	0.018
cg02776195	chr17:40486927	STAT3	0.121	0.007
cg08652441	chr17:40489513	STAT3	< 0.001	0.055
cg05487134	chr17:40489569	STAT3	0.001	0.072
cg24312520	chr17:40489584	STAT3	0.001	0.075
cg24718015	chr17:40489721	STAT3	0.585	0.002
cg17833746	chr17:40489785	STAT3	0.001	0.048
cg19438966	chr17:40491386	STAT3	< 0.001	0.016
cg01763935	chr17:40493229	STAT3	< 0.001	0.057
cg25745154	chr17:40493923	STAT3	0.224	0.009
cg25867318	chr17:40494745	STAT3	0.733	0.008
cg01619417	chr17:40507235	STAT3	0.023	0.055
cg08965162	chr17:40507919	STAT3	0.468	0.005
cg12873903	chr17:40514805	STAT3	0.376	0.003
cg06270615	chr17:40516068	STAT3	0.127	0.015
cg17748242	chr17:40517479	STAT3	0.151	0.003
cg15764884	chr17:40521020	STAT3	0.705	0.002
cg06350351	chr17:40526246	STAT3	< 0.001	0.012
cg17283534	chr17:40530242	STAT3	0.876	0.001
cg04598300	chr17:40531272	STAT3	0.041	0.006
cg25857307	chr17:40536562	STAT3	0.963	0.000
cg11897378	chr17:40539235	STAT3	0.002	0.018
cg07736922	chr17:40539702	STAT3	0.003	0.004
cg03131395	chr17:40540099	STAT3	0.212	0.002
cg18112163	chr17:40540197	STAT3	0.298	0.001
cg09804439	chr17:40540457	STAT3	0.749	0.000
cg06378498	chr17:40540460	STAT3	0.780	0.000
cg19557623	chr17:40540543	STAT3	0.748	0.001
cg15704988	chr17:40540562	STAT3	0.613	0.001
cg04517036	chr17:40540569	STAT3	0.834	0.000
cg01337508	chr17:40540572	STAT3	0.448	0.003
cg07158701	chr17:40540601	STAT3	0.857	0.001
cg17411949	chr17:40540650	STAT3	0.858	0.001
cg13655938	chr17:40540819	STAT3	0.930	0.001
cg09476841	chr17:40540860	STAT3	0.681	0.001
cg04076832	chr17:40540865	STAT3	0.005	0.006
cg20716209	chr17:40541270	STAT3	0.255	0.001
cg07322015	chr17:40541283	STAT3	0.969	0.000
cg03372410	chr2:168811754	STK39	0.879	0.002

cg21032473	chr2:168818347	STK39	0.151	0.003
cg01298735	chr2:168821882	STK39	0.140	0.005
cg19848140	chr2:168822706	STK39	< 0.001	0.038
cg08559214	chr2:168839903	STK39	0.038	0.007
cg07319162	chr2:168843898	STK39	0.946	0.000
cg06067477	chr2:168844818	STK39	0.021	0.013
cg07903472	chr2:168846657	STK39	0.550	0.004
cg08774104	chr2:168854864	STK39	0.069	0.007
cg08568260	chr2:168858485	STK39	0.002	0.018
cg17020782	chr2:168869861	STK39	0.938	0.000
cg17674892	chr2:168869998	STK39	< 0.001	0.043
cg08262269	chr2:168877862	STK39	0.007	0.008
cg06990256	chr2:168883365	STK39	0.300	0.003
cg12853654	chr2:168883913	STK39	0.015	0.013
cg05753044	chr2:168884280	STK39	0.655	0.001
cg09897958	chr2:168887429	STK39	0.795	0.001
cg08362162	chr2:168898872	STK39	0.276	0.011
cg19694335	chr2:168906876	STK39	0.023	0.008
cg05998565	chr2:168907444	STK39	0.063	0.022
cg13345122	chr2:168912885	STK39	0.640	0.010
cg14787753	chr2:168917992	STK39	0.094	0.006
cg03177034	chr2:168921027	STK39	0.531	0.001
cg11328284	chr2:168932113	STK39	0.176	0.044
cg02619264	chr2:168942650	STK39	0.393	0.003
cg19026692	chr2:168947421	STK39	0.475	0.009
cg24867784	chr2:168964414	STK39	0.089	0.003
cg10987981	chr2:168969276	STK39	0.059	0.007
cg05478553	chr2:168970426	STK39	0.014	0.015
cg10123669	chr2:168970900	STK39	0.188	0.047
cg26671568	chr2:168973489	STK39	0.052	0.008
cg02537014	chr2:168974265	STK39	0.002	0.009
cg26970032	chr2:168994440	STK39	0.414	0.008
cg03351487	chr2:169003820	STK39	0.149	0.050
cg16875057	chr2:169006003	STK39	< 0.001	0.055
cg12367789	chr2:169012049	STK39	0.102	0.004
cg09857870	chr2:169012428	STK39	0.648	0.012
cg03414543	chr2:169013814	STK39	0.514	0.003
cg12059950	chr2:169017661	STK39	0.009	0.027
cg08054187	chr2:169018258	STK39	0.033	0.011
cg27660720	chr2:169023572	STK39	0.853	0.005
cg10150255	chr2:169025365	STK39	0.150	0.011
cg06754557	chr2:169025892	STK39	0.470	0.004
cg22585786	chr2:169037808	STK39	0.003	0.015
cg22359623	chr2:169038705	STK39	0.202	0.004
cg03792447	chr2:169041897	STK39	0.898	0.007
cg20661391	chr2:169048192	STK39	0.077	0.004

cg11968742	chr2:169050271	STK39	0.049	0.018
cg12682817	chr2:169051315	STK39	0.001	0.021
cg26164761	chr2:169055243	STK39	0.030	0.061
cg07992385	chr2:169058521	STK39	< 0.001	0.045
cg08795597	chr2:169059614	STK39	0.032	0.012
cg10016283	chr2:169065139	STK39	0.091	0.009
cg17252489	chr2:169069249	STK39	0.136	0.018
cg08240917	chr2:169071559	STK39	0.934	0.001
cg13431028	chr2:169073609	STK39	0.057	0.006
cg06501606	chr2:169077032	STK39	0.057	0.005
cg15310214	chr2:169078031	STK39	0.417	0.002
cg24083378	chr2:169079247	STK39	0.345	0.003
cg21899461	chr2:169097849	STK39	0.001	0.060
cg11775828	chr2:169100915	STK39	< 0.001	0.055
cg24695024	chr2:169101015	STK39	< 0.001	0.063
cg03331300	chr2:169102866	STK39	0.722	0.000
cg02114728	chr2:169102929	STK39	0.473	0.002
cg22401940	chr2:169103153	STK39	0.047	0.006
cg23092844	chr2:169103738	STK39	0.482	0.001
cg07857142	chr2:169103971	STK39	0.372	0.001
cg21871154	chr2:169104283	STK39	0.698	0.000
cg19282745	chr2:169104342	STK39	0.976	0.001
cg02689023	chr2:169104449	STK39	0.014	0.003
cg24498376	chr2:169104453	STK39	0.108	0.002
cg26872305	chr2:169104523	STK39	0.684	0.001
cg22659356	chr2:169104591	STK39	0.209	0.003
cg08487618	chr2:169104715	STK39	< 0.001	0.035
cg10055458	chr2:169104734	STK39	0.007	0.017
cg03867702	chr2:169104828	STK39	0.005	0.042
cg22872692	chr2:169104856	STK39	0.042	0.025
cg18147479	chr2:169105070	STK39	0.655	0.010
cg23178697	chr2:169105091	STK39	0.188	0.014
cg18370778	chr12:64845191	TBK1	0.001	0.049
cg01859195	chr12:64845239	TBK1	< 0.001	0.059
cg06092729	chr12:64845451	TBK1	0.006	0.023
cg04466273	chr12:64845556	TBK1	0.637	0.001
cg22499688	chr12:64845600	TBK1	0.504	0.002
cg04671518	chr12:64845608	TBK1	0.577	0.001
cg27083278	chr12:64845641	TBK1	0.155	0.004
cg10074892	chr12:64845717	TBK1	0.901	0.000
cg07153406	chr12:64845733	TBK1	0.015	0.004
cg14498209	chr12:64845763	TBK1	0.784	0.001
cg07640083	chr12:64845769	TBK1	0.813	0.001
cg15709784	chr12:64845926	TBK1	0.043	0.001
cg21722680	chr12:64846210	TBK1	0.399	0.001
cg16604658	chr12:64847188	TBK1	0.005	0.045

cg23175599	chr12:64848891	TBK1	0.971	0.001
cg10069609	chr12:64852389	TBK1	0.100	0.007
cg04885749	chr12:64855513	TBK1	< 0.001	0.036
cg15343732	chr12:64862422	TBK1	0.759	0.001
cg09902198	chr12:64877389	TBK1	0.018	0.075
cg09999583	chr12:64878162	TBK1	0.037	0.007
cg13540592	chr12:64889343	TBK1	0.003	0.009
cg01089930	chr12:64890764	TBK1	0.256	0.006
cg19796955	chr12:64895570	TBK1	0.045	0.004
cg22510067	chr2:137747619	THSD7B	0.091	0.007
cg08084967	chr2:137747719	THSD7B	0.363	0.005
cg08967662	chr2:137747749	THSD7B	0.163	0.006
cg25714147	chr2:137747982	THSD7B	0.031	0.008
cg07412043	chr2:137748366	THSD7B	0.763	0.000
cg15750500	chr2:137748382	THSD7B	0.072	0.007
cg06848148	chr2:137748455	THSD7B	0.535	0.002
cg08231096	chr2:137748460	THSD7B	0.014	0.018
cg11746464	chr2:137748833	THSD7B	0.752	0.009
cg08222788	chr2:137754925	THSD7B	0.233	0.017
cg06745308	chr2:137755968	THSD7B	0.034	0.018
cg16561226	chr2:137808428	THSD7B	0.050	0.036
cg24449653	chr2:137813993	THSD7B	0.171	0.028
cg03897905	chr2:137814066	THSD7B	0.820	0.000
cg05764108	chr2:137814211	THSD7B	0.741	0.004
cg15834121	chr2:137814271	THSD7B	0.973	0.000
cg16579593	chr2:137814280	THSD7B	0.364	0.003
cg14255655	chr2:137891808	THSD7B	0.957	0.000
cg02375649	chr2:137912216	THSD7B	0.016	0.018
cg01103321	chr2:137913067	THSD7B	0.487	0.007
cg04578489	chr2:137913401	THSD7B	0.955	0.003
cg08633789	chr2:137913470	THSD7B	0.009	0.017
cg01709492	chr2:137921113	THSD7B	0.784	0.004
cg12906989	chr2:137925983	THSD7B	0.481	0.008
cg11737758	chr2:137931242	THSD7B	0.020	0.040
cg00743354	chr2:137935851	THSD7B	0.250	0.019
cg01439023	chr2:137936057	THSD7B	0.151	0.011
cg23554286	chr2:137951070	THSD7B	0.038	0.019
cg08200809	chr2:137959323	THSD7B	0.326	0.004
cg06319861	chr2:137964351	THSD7B	0.742	0.002
cg01086974	chr2:137986170	THSD7B	< 0.001	0.039
cg13431597	chr2:137987479	THSD7B	0.655	0.002
cg04667011	chr2:138011944	THSD7B	0.333	0.011
cg05853130	chr2:138019701	THSD7B	0.677	0.008
cg25498731	chr2:138045743	THSD7B	0.241	0.011
cg25073409	chr2:138091239	THSD7B	0.182	0.016
cg22012786	chr2:138106132	THSD7B	0.286	0.007

cg05463672	chr2:138224937	THSD7B	0.551	0.005
cg03526165	chr2:138264722	THSD7B	0.712	0.004
cg11795053	chr2:138399663	THSD7B	0.015	0.025
cg17836195	chr2:138416978	THSD7B	0.038	0.008
cg21924447	chr2:138427803	THSD7B	< 0.001	0.038
cg21495956	chr2:138434773	THSD7B	0.527	0.006
cg26648758	chr19:4816176	TICAM1	0.851	0.002
cg00351190	chr19:4816855	TICAM1	0.022	0.013
cg10221736	chr19:4818359	TICAM1	0.931	0.000
cg26372557	chr19:4818615	TICAM1	0.714	0.002
cg00423871	chr19:4827986	TICAM1	0.518	0.005
cg02752037	chr19:4829518	TICAM1	< 0.001	0.040
cg04674143	chr19:4831543	TICAM1	0.113	0.001
cg22770755	chr19:4831688	TICAM1	0.679	0.001
cg02430562	chr19:4831721	TICAM1	0.162	0.001
cg13119687	chr19:4831825	TICAM1	0.158	0.001
cg02759622	chr19:4831885	TICAM1	0.585	0.001
cg00087799	chr19:4831891	TICAM1	0.616	0.001
cg16722792	chr19:4831939	TICAM1	0.958	0.000
cg23801786	chr19:4831951	TICAM1	0.494	0.003
cg19751098	chr19:4831974	TICAM1	0.535	0.002
cg15457276	chr19:4832024	TICAM1	0.329	0.005
cg19797341	chr19:4832695	TICAM1	0.037	0.013
cg07550579	chr19:4832918	TICAM1	< 0.001	0.041
cg07649585	chr19:4832993	TICAM1	0.931	0.003
cg09295300	chr4:186989025	TLR3	0.304	0.005
cg00305797	chr4:186989236	TLR3	0.631	0.018
cg01036205	chr4:186989523	TLR3	0.732	0.005
cg15851014	chr4:186989764	TLR3	0.903	0.016
cg06498520	chr4:186990006	TLR3	0.010	0.014
cg17671280	chr4:186990202	TLR3	0.244	0.010
cg25367691	chr4:186990324	TLR3	< 0.001	0.010
cg12697789	chr4:186990425	TLR3	0.923	0.000
cg11273820	chr4:187002591	TLR3	0.474	0.005
cg14827929	chr4:187002617	TLR3	0.836	0.002
cg01414772	chr4:187002764	TLR3	0.427	0.004
cg00306510	chr4:187003000	TLR3	0.455	0.022
cg05824016	chrX:15651523	TMEM27	0.913	0.002
cg11336696	chrX:15663437	TMEM27	0.086	0.020
cg15226856	chrX:15665166	TMEM27	0.907	0.000
cg01282374	chrX:15666287	TMEM27	0.982	0.001
cg11478498	chrX:15672946	TMEM27	0.060	0.051
cg20473453	chrX:15683007	TMEM27	0.216	0.018
cg26062907	chrX:15683192	TMEM27	0.255	0.011
cg05779963	chrX:15683204	TMEM27	0.588	0.007
cg12134159	chrX:15683282	TMEM27	0.265	0.029

cg05219621	chrX:15683403	TMEM27	0.330	0.034
cg06454337	chrX:15683427	TMEM27	0.657	0.010
cg13876499	chrX:15683696	TMEM27	0.523	0.014
cg27087885	chrX:15683977	TMEM27	0.488	0.016
cg18156003	chr21:42836963	TMPRSS2	0.913	0.000
cg19020860	chr21:42837277	TMPRSS2	0.098	0.011
cg19974120	chr21:42839625	TMPRSS2	0.901	0.001
cg12203615	chr21:42840167	TMPRSS2	0.092	0.017
cg02610779	chr21:42840442	TMPRSS2	0.313	0.013
cg02978411	chr21:42840898	TMPRSS2	0.318	0.005
cg02702369	chr21:42844174	TMPRSS2	0.282	0.007
cg16385367	chr21:42844842	TMPRSS2	0.225	0.011
cg00255189	chr21:42847772	TMPRSS2	0.624	0.004
cg02642997	chr21:42849701	TMPRSS2	0.532	0.007
cg05218995	chr21:42851888	TMPRSS2	0.946	0.001
cg02432628	chr21:42853308	TMPRSS2	0.055	0.007
cg13698049	chr21:42870391	TMPRSS2	0.133	0.009
cg07808966	chr21:42873179	TMPRSS2	0.628	0.006
cg07919906	chr21:42873733	TMPRSS2	0.589	0.005
cg13489049	chr21:42875961	TMPRSS2	0.054	0.012
cg01157146	chr21:42877638	TMPRSS2	0.456	0.008
cg22025068	chr21:42878726	TMPRSS2	0.056	0.017
cg16084872	chr21:42879074	TMPRSS2	0.002	0.016
cg02613803	chr21:42879131	TMPRSS2	0.183	0.007
cg00739644	chr21:42879658	TMPRSS2	0.200	0.019
cg26337277	chr21:42879998	TMPRSS2	0.104	0.002
cg05563672	chr21:42880077	TMPRSS2	0.753	0.000
cg19315202	chr21:42880095	TMPRSS2	0.741	0.001
cg00689211	chr21:42880107	TMPRSS2	0.569	0.003
cg15014277	chr21:42880145	TMPRSS2	0.610	0.001
cg01036509	chr21:42880231	TMPRSS2	0.518	0.002
cg16371860	chr21:42880448	TMPRSS2	0.269	0.010
cg24901042	chr21:42880554	TMPRSS2	0.130	0.008
cg26309194	chr21:42880557	TMPRSS2	0.534	0.005
cg14982276	chr21:42880582	TMPRSS2	0.045	0.005
cg12384236	chr21:42880714	TMPRSS2	0.097	0.003
cg22484980	chr11:68815449	TPCN2	0.007	0.031
cg26755111	chr11:68815586	TPCN2	0.022	0.014
cg23247191	chr11:68815682	TPCN2	0.501	0.007
cg22915154	chr11:68816030	TPCN2	0.071	0.019
cg25170956	chr11:68816067	TPCN2	0.530	0.003
cg01930488	chr11:68816150	TPCN2	0.637	0.003
cg26054232	chr11:68816191	TPCN2	0.040	0.004
cg02529490	chr11:68816232	TPCN2	0.117	0.018
cg15335525	chr11:68816274	TPCN2	0.003	0.008
cg19359505	chr11:68816308	TPCN2	0.873	0.000

cg08278582	chr11:68816414	TPCN2	0.139	0.001
cg10490196	chr11:68816451	TPCN2	0.522	0.001
cg27481141	chr11:68816453	TPCN2	0.481	0.001
cg06096695	chr11:68816637	TPCN2	0.861	0.000
cg23121140	chr11:68816938	TPCN2	0.735	0.001
cg06624991	chr11:68818378	TPCN2	0.063	0.012
cg10256045	chr11:68819068	TPCN2	0.650	0.002
cg11999939	chr11:68821415	TPCN2	0.883	0.002
cg07827609	chr11:68821754	TPCN2	0.531	0.007
cg27389775	chr11:68822072	TPCN2	0.012	0.016
cg07959156	chr11:68822506	TPCN2	0.242	0.006
cg17980216	chr11:68822533	TPCN2	0.198	0.004
cg00603982	chr11:68822544	TPCN2	0.520	0.002
cg05951984	chr11:68823811	TPCN2	0.024	0.037
cg12411713	chr11:68825135	TPCN2	0.564	0.002
cg01241272	chr11:68825150	TPCN2	0.590	0.003
cg11648404	chr11:68830440	TPCN2	0.369	0.004
cg22748539	chr11:68830527	TPCN2	0.560	0.001
cg23671189	chr11:68830561	TPCN2	0.790	0.000
cg06093500	chr11:68832773	TPCN2	0.859	0.002
cg08478914	chr11:68839802	TPCN2	0.375	0.002
cg02263035	chr11:68840389	TPCN2	0.510	0.008
cg11361442	chr11:68841024	TPCN2	0.133	0.012
cg17345676	chr11:68842069	TPCN2	0.227	0.019
cg09801706	chr11:68842350	TPCN2	0.320	0.003
cg26828599	chr11:68842998	TPCN2	0.471	0.003
cg15572478	chr11:68843521	TPCN2	0.191	0.019
cg06837675	chr11:68845932	TPCN2	0.820	0.003
cg03472453	chr11:68846042	TPCN2	0.782	0.002
cg13952997	chr11:68846099	TPCN2	0.222	0.005
cg20267284	chr11:68846972	TPCN2	0.002	0.066
cg15986248	chr11:68847470	TPCN2	0.020	0.007
cg18503234	chr11:68847839	TPCN2	0.919	0.013
cg10412127	chr11:68848139	TPCN2	0.001	0.040
cg26433582	chr11:68848232	TPCN2	0.655	0.002
cg05948321	chr11:68848277	TPCN2	0.017	0.024
cg16006626	chr11:68849960	TPCN2	0.935	0.000
cg17467664	chr11:68851580	TPCN2	0.515	0.002
cg01993067	chr11:68851601	TPCN2	0.260	0.003
cg01403660	chr11:68851641	TPCN2	0.891	0.002
cg20256574	chr11:68851821	TPCN2	0.624	0.002
cg09651302	chr11:68851880	TPCN2	0.398	0.003
cg21186438	chr11:68851932	TPCN2	0.937	0.000
cg16998906	chr11:68853398	TPCN2	0.015	0.011
cg24868307	chr11:68853686	TPCN2	0.524	0.003
cg18053228	chr11:68853915	TPCN2	0.379	0.006

cg16005564	chr11:68853957	TPCN2	0.051	0.006
cg08847919	chr11:68854196	TPCN2	0.192	0.003
cg04888485	chr11:68855216	TPCN2	0.699	0.005
cg25042173	chr11:68855823	TPCN2	0.080	0.007
cg01385198	chr11:68856225	TPCN2	0.003	0.022
cg16709353	chr11:68856817	TPCN2	0.746	0.005
cg02584610	chr11:68856883	TPCN2	0.635	0.003
cg12152352	chr11:68856906	TPCN2	0.004	0.032
cg13874366	chr11:68856951	TPCN2	0.181	0.009
cg13834366	chr11:68857566	TPCN2	0.898	0.000
cg01030178	chr8:67037892	TRIM55	0.031	0.010
cg24515709	chr8:67038022	TRIM55	0.367	0.004
cg27035277	chr8:67038272	TRIM55	0.685	0.003
cg11515052	chr8:67038980	TRIM55	0.963	0.006
cg23322523	chr8:67039041	TRIM55	0.011	0.013
cg25914706	chr8:67039068	TRIM55	0.634	0.002
cg07180197	chr8:67039155	TRIM55	0.005	0.024
cg27465837	chr8:67039178	TRIM55	0.013	0.022
cg19975482	chr8:67039256	TRIM55	0.141	0.007
cg04145765	chr8:67039461	TRIM55	0.097	0.008
cg14418968	chr8:67040331	TRIM55	0.010	0.015
cg25922284	chr8:67042176	TRIM55	0.004	0.075
cg00793977	chr8:67042666	TRIM55	< 0.001	0.020
cg10986302	chr8:67045991	TRIM55	0.886	0.001
cg21165595	chr8:67046248	TRIM55	0.362	0.008
cg26104239	chr8:67047851	TRIM55	0.175	0.004
cg10479107	chr8:67049448	TRIM55	0.086	0.024
cg25483191	chr8:67050040	TRIM55	0.623	0.001
cg09173500	chr8:67053592	TRIM55	0.620	0.002
cg23973532	chr8:67054592	TRIM55	0.475	0.016
cg01352267	chr8:67064467	TRIM55	0.043	0.008
cg26700215	chr8:67070006	TRIM55	0.839	0.001
cg04041976	chr8:67085823	TRIM55	0.554	0.002
cg16888621	chr8:67085866	TRIM55	0.752	0.001
cg07515324	chr19:10461336	TYK2	0.143	0.008
cg02319549	chr19:10463037	TYK2	0.780	0.001
cg13971552	chr19:10463352	TYK2	0.866	0.002
cg03889617	chr19:10463828	TYK2	0.963	0.000
cg21480173	chr19:10463831	TYK2	0.941	0.000
cg23306063	chr19:10463879	TYK2	0.280	0.005
cg02512212	chr19:10464100	TYK2	0.041	0.011
cg18038269	chr19:10464320	TYK2	0.282	0.002
cg11575644	chr19:10464711	TYK2	0.128	0.004
cg19925475	chr19:10464767	TYK2	0.478	0.003
cg00590639	chr19:10464771	TYK2	0.388	0.000
cg23184299	chr19:10464922	TYK2	0.613	0.004

cg11369662	chr19:10465257	TYK2	0.698	0.000
cg06917623	chr19:10467366	TYK2	0.809	0.002
cg25613873	chr19:10468706	TYK2	0.844	0.001
cg07488370	chr19:10472265	TYK2	0.116	0.008
cg18892020	chr19:10472451	TYK2	0.440	0.004
cg00079643	chr19:10472658	TYK2	0.427	0.006
cg14025953	chr19:10472966	TYK2	0.847	0.001
cg11057783	chr19:10473307	TYK2	0.851	0.001
cg12184486	chr19:10474184	TYK2	0.554	0.001
cg01547781	chr19:10474464	TYK2	0.490	0.004
cg10144807	chr19:10474912	TYK2	0.154	0.007
cg05314616	chr19:10475269	TYK2	0.871	0.002
cg06935109	chr19:10476554	TYK2	0.353	0.001
cg26244993	chr19:10477232	TYK2	0.524	0.006
cg26142436	chr19:10479250	TYK2	0.264	0.007
cg19307883	chr19:10484624	TYK2	0.610	0.007
cg15197202	chr19:10488965	TYK2	0.153	0.008
cg22554952	chr19:10489088	TYK2	0.067	0.005
cg18611797	chr19:10489256	TYK2	0.086	0.009
cg25395945	chr19:10489394	TYK2	0.200	0.015
cg24226087	chr19:10490782	TYK2	0.664	0.001
cg18856252	chr19:10491173	TYK2	0.727	0.001
cg15079565	chr19:10491255	TYK2	0.959	0.000
cg11331422	chr19:10491258	TYK2	0.822	0.000
cg25676786	chr19:10491363	TYK2	0.031	0.004
cg16068727	chr19:10491392	TYK2	0.456	0.002
cg21941105	chr19:10491405	TYK2	0.208	0.002
cg15981620	chr19:10491431	TYK2	0.014	0.003
cg06622468	chr19:10491460	TYK2	0.954	0.002
cg08896629	chr19:10491577	TYK2	0.989	0.000
cg22140136	chr19:10491633	TYK2	0.680	0.003
cg11400613	chr19:10491835	TYK2	0.144	0.005
cg26265787	chr19:10491889	TYK2	0.025	0.030
cg18069484	chr11:67764105	UNC93B1	0.335	0.002
cg17066594	chr11:67764654	UNC93B1	0.226	0.047
cg15617336	chr11:67764948	UNC93B1	0.758	0.000
cg08480739	chr11:67765021	UNC93B1	0.025	0.010
cg03267954	chr11:67765141	UNC93B1	0.047	0.013
cg20272935	chr11:67765720	UNC93B1	0.037	0.028
cg09145322	chr11:67766631	UNC93B1	0.117	0.010
cg19469649	chr11:67766769	UNC93B1	0.187	0.007
cg07744435	chr11:67768362	UNC93B1	0.300	0.010
cg01711344	chr11:67770404	UNC93B1	0.013	0.034
cg11027221	chr11:67770665	UNC93B1	< 0.001	0.032
cg16904500	chr11:67770959	UNC93B1	0.089	0.002
cg15260365	chr11:67771606	UNC93B1	0.415	0.005

cg25756680	chr11:67771726	UNC93B1	0.999	0.001
cg04108360	chr11:67771791	UNC93B1	0.076	0.019
cg03235688	chr11:67771801	UNC93B1	0.001	0.026
cg13868942	chr11:67771812	UNC93B1	0.008	0.020
cg02044612	chr11:67772000	UNC93B1	0.415	0.006
cg11200858	chr11:67772273	UNC93B1	0.022	0.021
cg11733617	chr11:67772601	UNC93B1	0.237	0.005
cg22447416	chr11:67772826	UNC93B1	0.989	0.001
cg27027206	chr13:42151743	VWA8	< 0.001	0.077
cg21497029	chr13:42161739	VWA8	0.001	0.017
cg25568501	chr13:42162968	VWA8	0.635	0.002
cg03810562	chr13:42165082	VWA8	0.481	0.006
cg27099134	chr13:42180866	VWA8	0.123	0.005
cg03795663	chr13:42188218	VWA8	0.211	0.003
cg02243249	chr13:42188453	VWA8	0.484	0.002
cg10341691	chr13:42219219	VWA8	0.604	0.002
cg12099998	chr13:42222230	VWA8	0.061	0.012
cg09580278	chr13:42222676	VWA8	0.073	0.006
cg08659034	chr13:42224070	VWA8	0.024	0.015
cg12434040	chr13:42227088	VWA8	0.402	0.013
cg11430223	chr13:42232607	VWA8	0.096	0.021
cg22241686	chr13:42242375	VWA8	0.006	0.054
cg21860329	chr13:42265546	VWA8	< 0.001	0.164
cg07584516	chr13:42269266	VWA8	< 0.001	0.111
cg12396757	chr13:42271163	VWA8	0.074	0.012
cg21829495	chr13:42295758	VWA8	0.717	0.002
cg23573337	chr13:42325829	VWA8	0.044	0.011
cg05252898	chr13:42362736	VWA8	0.001	0.036
cg07717466	chr13:42362761	VWA8	< 0.001	0.051
cg06130225	chr13:42386036	VWA8	0.983	0.010
cg13629862	chr13:42387252	VWA8	< 0.001	0.029
cg02008703	chr13:42395382	VWA8	0.295	0.020
cg26177408	chr13:42402429	VWA8	0.147	0.018
cg12005153	chr13:42412158	VWA8	0.924	0.001
cg22945982	chr13:42443309	VWA8	0.136	0.013
cg23336397	chr13:42453261	VWA8	0.354	0.014
cg13058467	chr13:42477894	VWA8	0.760	0.001
cg02523709	chr13:42478085	VWA8	0.060	0.007
cg24929734	chr13:42478092	VWA8	0.114	0.008
cg08767171	chr13:42491015	VWA8	0.693	0.003
cg25682700	chr13:42493215	VWA8	0.624	0.007
cg00146247	chr13:42516684	VWA8	0.168	0.004
cg15806257	chr13:42519184	VWA8	0.058	0.009
cg11224904	chr13:42530006	VWA8	0.003	0.067
cg07217215	chr13:42535514	VWA8	0.122	0.013
cg13431152	chr3:46062321	XCR1	0.132	0.012

cg09257432	chr3:46062588	XCR1	0.134	0.009
cg04860142	chr3:46062662	XCR1	0.827	0.001
cg19967421	chr3:46062881	XCR1	0.792	0.000
cg12691230	chr3:46064611	XCR1	0.263	0.005
cg10360403	chr3:46064716	XCR1	0.142	0.008
cg08459918	chr3:46065328	XCR1	0.337	0.007
cg15888522	chr3:46068932	XCR1	0.005	0.012
cg10096268	chr3:46070193	XCR1	0.269	0.005
cg12931860	chr3:46070399	XCR1	0.850	0.001

Notes: CpG ID corresponds to the unique CpG site identifier in the HumanMethylationEPIC array (Illumina). Chromosomal location denoted according human reference assembly GRCh37/hg19. All depicted P values are FDR adjusted P values. CpG-sites with an absolute mean methylation beta value difference > 0.15 and FDR adjusted P value < 0.05 were considered statistically significant.

*Included studies:

Bastard P, Rosen LB, Zhang Q, et al. Autoantibodies against type I IFNs in patients with life-threatening COVID-19. *Science* 2020; **370**: eabd4585.

Mousa M, Vurivi H, Kannout H, et al. Genome-wide association study of hospitalized COVID-19 patients in the United Arab Emirates. *EBioMedicine* 2021; **74**: 103695.

Pairo-Castineira E, Clohisey S, Klaric L, et al. Genetic mechanisms of critical illness in Covid-19. *Nature* 2020; **591**: 92–98.

Severe Covid-19 GWAS Group, Ellinghaus D, Degenhardt F, et al. Genomewide association study of severe Covid-19 with respiratory failure. *N Engl J* Med 2020; **383**: 1522–34.

Singh H, Choudhari R, Nema V, Khan AA. ACE2 and TMPRSS2 polymorphisms in various diseases with special reference to its impact on COVID-19 disease. *Microb Pathog* 2020; **150**: 104621.

van der Made CI, Simons A, Schuurs-Hoeijmakers J, et al. Presence of genetic variants among young men with severe COVID-19. *JAMA* 2020; **324**: 1–11.

Zhang Q, Bastard P, Liu Z, et al. Inborn errors of type I IFN immunity in patients with life-threatening COVID-19. *Science* 2020; **370**: eabd4570.

Supplementary Table S5. Description of the 68 CpG sites in the 3 genes related to MIS-C, based on published studies*.

CpG ID	Chromosome location	Gene name	P value	Absolute mean methylation difference
cg09938226	chrX:37641307	CYBB	0.765	0.039
cg03198297	chrX:37646886	CYBB	0.914	0.026
cg17037297	chrX:37649522	CYBB	0.354	0.074
cg12290270	chrX:37656559	CYBB	0.713	0.050
cg21794913	chrX:37656644	CYBB	0.048	0.036
cg21065784	chrX:37656759	CYBB	0.978	0.049
cg07525792	chrX:37659262	CYBB	0.409	0.017
cg15235844	chrX:37671328	CYBB	0.151	0.030
cg03014241	chr16:11348611	SOCS1	0.816	0.040
cg10784813	chr16:11348678	SOCS1	0.766	0.012
cg04004558	chr16:11348684	SOCS1	0.946	0.016
cg04266460	chr16:11348956	SOCS1	0.002	0.003
cg06220235	chr16:11349023	SOCS1	< 0.001	0.003
cg01717706	chr16:11349308	SOCS1	0.069	0.002
cg26012103	chr16:11349372	SOCS1	0.079	0.001
cg00487159	chr16:11349568	SOCS1	0.369	0.002
cg11973052	chr16:11349832	SOCS1	< 0.001	0.001
cg27003951	chr16:11350045	SOCS1	< 0.001	0.001
cg27540841	chr16:11350112	SOCS1	< 0.001	0.001
cg03553358	chr16:11350115	SOCS1	0.676	0.024
cg12642006	chr16:11350118	SOCS1	0.434	0.006
cg26233182	chr16:11350235	SOCS1	0.170	0.002
cg01330880	chr16:11350249	SOCS1	0.049	0.000
cg06082432	chr16:11350275	SOCS1	0.106	0.001
cg06295404	chr16:11350337	SOCS1	0.021	0.000
cg09567644	chr16:11350363	SOCS1	0.033	0.002
cg03195600	chr16:11350371	SOCS1	0.101	0.008
cg05766667	chr16:11350740	SOCS1	< 0.001	0.001
cg00674265	chr16:11350746	SOCS1	0.205	0.000
cg26301908	chr16:11350915	SOCS1	0.618	0.007
cg10513253	chr16:11351136	SOCS1	0.696	0.000
cg05181221	chr16:11351158	SOCS1	0.926	0.001
cg04289163	chr16:11351165	SOCS1	0.280	0.001
cg04609482	chr16:11351169	SOCS1	0.956	0.017
cg05902273	chr16:11351199	SOCS1	0.542	0.003
cg05730996	chr16:11351330	SOCS1	0.155	0.007
cg01490918	chrX:122992224	XIAP	0.804	0.005
cg23933262	chrX:122993037	XIAP	0.262	0.086
cg02386862	chrX:122993369	XIAP	0.522	0.045
cg00920960	chrX:122993419	XIAP	0.504	0.071
cg21494642	chrX:122993581	XIAP	0.527	0.066

cg03966760	chrX:122993583	XIAP	0.581	0.036
cg06772067	chrX:122993679	XIAP	0.526	0.088
cg21945665	chrX:122993695	XIAP	0.463	0.026
cg10873964	chrX:122993706	XIAP	0.442	0.094
cg08282969	chrX:122993891	XIAP	0.633	0.080
cg07874334	chrX:122993913	XIAP	0.464	0.086
cg04302178	chrX:122993941	XIAP	0.480	0.110
cg17033281	chrX:122993976	XIAP	0.528	0.072
cg17703220	chrX:122993978	XIAP	0.458	0.073
cg21711683	chrX:122994030	XIAP	0.477	0.083
cg16778620	chrX:122994037	XIAP	0.851	0.067
cg09950034	chrX:122994069	XIAP	0.968	0.079
cg12375308	chrX:122994071	XIAP	0.606	0.078
cg21723696	chrX:122994177	XIAP	0.464	0.044
cg25514427	chrX:122994594	XIAP	0.485	0.092
cg05198817	chrX:122995701	XIAP	0.862	0.041
cg11315455	chrX:122997585	XIAP	0.056	0.018
cg10095090	chrX:123008155	XIAP	0.513	0.021
cg11585623	chrX:123008314	XIAP	0.261	0.012
cg06895894	chrX:123011980	XIAP	0.559	0.049
cg20908014	chrX:123019820	XIAP	0.610	0.012
cg12794939	chrX:123033885	XIAP	0.155	0.018
cg13886984	chrX:123038397	XIAP	0.623	0.009
cg06742180	chrX:123039177	XIAP	0.308	0.074
cg03170186	chrX:123039334	XIAP	0.041	0.003
cg14311341	chrX:123040677	XIAP	0.644	0.072
cg02877744	chrX:123047040	XIAP	0.660	0.008

Notes: CpG ID corresponds to the unique CpG site identifier in the HumanMethylationEPIC array (Illumina). Chromosomal location denoted according human reference assembly GRCh37/hg19. All depicted P values are FDR adjusted P values. CpG-sites with an absolute mean methylation beta value difference > 0.15 and FDR adjusted P value < 0.05 were considered statistically significant.

*Included studies:

Chou J, Platt CD, Habiballah S, et al. Mechanisms underlying genetic susceptibility to multisystem inflammatory syndrome in children (MIS-C). *J Allergy Clin Immunol* 2021; **148**(3): 732–738.e1.

Lee PY, Platt CD, Weeks S, et al. Immune dysregulation and multisystem inflammatory syndrome in children (MIS-C) in individuals with haploinsufficiency of SOCS1. *J Allergy Clin Immunol* 2020; **146**(5): 1194–1200.e1.

Supplementary Table S6. EPIMISC derived-CpGs in the validation and entire cohorts, according to the study pipeline described in Supplementary Figure S1.

CpG ID	Chromosome location	Gene name	Validation cohort		Entire cohort	
			P value	Absolute mean methylation difference	P value	Absolute mean methylation difference
cg03776649	chr5:125465533		0.005	0.369	< 0.001	0.248
cg20995564	chr2:145172035	ZEB2	0.001	0.284	< 0.001	0.277
cg22203628	chr11:69241075		0.003	0.265	< 0.001	0.213
cg20292908	chr5:172203421		0.001	0.262	< 0.001	0.285
cg26830054	chr10:119762394		0.003	0.244	< 0.001	0.215
cg15033511	chr17:54994023		0.003	0.237	< 0.001	0.210
cg06135068	chr16:31034016		< 0.001	0.233	< 0.001	0.223
cg21860329	chr13:42265546	VWA8	0.003	0.218	< 0.001	0.182
cg13178755	chr2:174023580	ZAK	0.004	0.192	< 0.001	0.166
cg04544473	chr4:153021978	LOC100996286	0.004	0.189	< 0.001	0.164
cg16729631	chr8:131000261		0.004	0.185	< 0.001	0.163
cg20059012	chr12:53613154	RARG	0.009	0.181	< 0.001	0.162
cg11023668	chr2:25095040	ADCY3	0.004	0.178	< 0.001	0.160
cg17279147	chr6:14739369		0.016	0.176	< 0.001	0.159
cg22994883	chr7:130615334		0.003	0.171	< 0.001	0.157
cg01297684	chr12:56069634		0.003	0.165	< 0.001	0.177
cg14887853	chr6:139794538	LOC645434	0.006	0.164	< 0.001	0.162
cg12662084	chr6:17809126	KIF13A	0.003	0.162	< 0.001	0.159
cg20361768	chr3:156819083	LINC00880	0.003	0.152	< 0.001	0.171
cg17515347	chr1:159047163	AIM2	0.008	0.151	< 0.001	0.151
cg21963178	chr10:75571738	NDST2	0.419	0.114	0.001	0.156
cg18066211	chr4:99369082		0.016	0.114	< 0.001	0.139
cg05712639	chr14:52819386		0.027	0.114	< 0.001	0.150
cg24433124	chr6:30755968		0.393	0.113	0.001	0.152
cg16402757	chr10:35311004	CUL2	0.677	0.087	0.017	0.173
cg16600909	chr1:173145001		0.466	0.074	0.003	0.129
cg01062020	chr1:162382848	SH2D1B	0.295	0.068	< 0.001	0.152
cg08776296	chr7:134856544	CYREN	0.364	0.055	0.003	0.120
cg06386482	chr6:47624117	GPR111	0.587	0.049	0.007	0.104
cg02578087	chr3:8671361	SSUH2	0.373	0.048	0.004	0.139
cg13910785	chr6:32549849	HLA-DRB1	0.987	0.032	0.078	0.104
cg07167872	chr1:205819463	PM20D1	0.930	0.011	0.053	0.105
cg03192273	chr5:150618948		0.995	0.004	0.054	0.111

Notes: CpG ID corresponds to the unique CpG site identifier in the HumanMethylationEPIC array (Illumina). Chromosomal location denoted according human reference assembly GRCh37/hg19. All depicted P values are FDR adjusted P values. CpG-sites with an absolute mean methylation beta value difference > 0.15 and FDR adjusted P value < 0.05 were considered statistically significant.

Supplementary Table S7. Description of the 1350 CpG sites in the 33 genes related to Kawasaki disease, based on published studies*.

CpG ID	Chromosome location	Gene name	P value	Absolute mean methylation difference
cg04986849	chr8:11350092	BLK	0.718	0.014
cg10147394	chr8:11350297	BLK	0.962	0.003
cg15683292	chr8:11350515	BLK	0.297	0.002
cg15742700	chr8:11350853	BLK	0.985	0.020
cg13440894	chr8:11351135	BLK	0.154	0.012
cg22826986	chr8:11351507	BLK	0.572	0.009
cg03860768	chr8:11351571	BLK	0.608	0.005
cg18591982	chr8:11351583	BLK	0.695	0.006
cg27406521	chr8:11351770	BLK	0.071	0.003
cg07959027	chr8:11351835	BLK	0.918	0.008
cg19882315	chr8:11351846	BLK	0.521	0.018
cg00580089	chr8:11355562	BLK	0.157	0.017
cg00018682	chr8:11359569	BLK	0.575	0.007
cg05321338	chr8:11365455	BLK	0.124	0.000
cg04578612	chr8:11365915	BLK	0.486	0.004
cg21497594	chr8:11366745	BLK	0.922	0.008
cg10812376	chr8:11367985	BLK	0.306	0.005
cg09205687	chr8:11370624	BLK	0.053	0.006
cg21701351	chr8:11374773	BLK	0.815	0.003
cg02034205	chr8:11382291	BLK	0.884	0.004
cg12571819	chr8:11387351	BLK	0.804	0.003
cg14921801	chr8:11389209	BLK	0.007	0.015
cg23507676	chr8:11395642	BLK	0.489	0.016
cg08361089	chr8:11396323	BLK	0.003	0.014
cg07608611	chr8:11402219	BLK	0.034	0.009
cg03002059	chr8:11402647	BLK	0.319	0.001
cg10343719	chr8:11403233	BLK	0.164	0.016
cg01879562	chr8:11403516	BLK	0.308	0.003
cg21410383	chr8:11404830	BLK	0.794	0.003
cg13835576	chr8:11407354	BLK	0.295	0.048
cg19384726	chr8:11409058	BLK	0.221	0.003
cg12641275	chr8:11411487	BLK	0.698	0.006
cg01538039	chr8:11411788	BLK	0.620	0.013
cg20652749	chr8:11411795	BLK	0.335	0.009
cg16554881	chr8:11412199	BLK	0.259	0.005
cg15685006	chr8:11413043	BLK	0.655	0.003
cg26161004	chr8:11413186	BLK	0.927	0.002
cg14702231	chr8:11413234	BLK	0.288	0.021
cg02978838	chr8:11413591	BLK	0.092	0.009
cg03286662	chr8:11414004	BLK	0.993	0.005

cg07623233	chr8:11415543	BLK	0.438	0.095
cg11899815	chr8:11415578	BLK	0.184	0.098
cg10037292	chr8:11415838	BLK	0.881	0.010
cg20900678	chr8:11416319	BLK	0.142	0.003
cg13533935	chr8:11416365	BLK	0.007	0.002
cg04335815	chr8:11417517	BLK	0.401	0.002
cg19655051	chr8:11418372	BLK	0.516	0.001
cg07764073	chr8:11419707	BLK	0.887	0.001
cg21175976	chr8:11421337	BLK	0.880	0.009
cg02910793	chr8:11421934	BLK	0.003	0.002
cg13339708	chr4:185549367	CASP3	0.322	0.002
cg20176042	chr4:185552304	CASP3	0.303	0.007
cg25028736	chr4:185556747	CASP3	0.965	0.083
cg09185219	chr4:185566292	CASP3	0.351	0.006
cg19035151	chr4:185566711	CASP3	0.206	0.002
cg19290403	chr4:185567382	CASP3	0.209	0.006
cg07234880	chr4:185570559	CASP3	0.313	0.001
cg00444120	chr4:185570857	CASP3	0.018	0.001
cg23259381	chr4:185570876	CASP3	0.004	0.001
cg20811273	chr4:185570979	CASP3	0.117	0.001
cg21490662	chr4:185571520	CASP3	0.181	0.004
cg12167823	chr4:185571549	CASP3	0.398	0.008
cg15171237	chr4:185571958	CASP3	0.420	0.001
cg01149415	chr20:44745522	CD40	0.074	0.010
cg09053081	chr20:44746392	CD40	0.262	0.001
cg17929951	chr20:44746681	CD40	0.133	0.002
cg11841529	chr20:44746751	CD40	0.230	0.005
cg06571407	chr20:44746823	CD40	0.574	0.026
cg22232207	chr20:44746825	CD40	0.593	0.027
cg24575067	chr20:44746902	CD40	0.837	0.004
cg01943874	chr20:44746944	CD40	0.298	0.006
cg21601405	chr20:44747006	CD40	0.118	0.004
cg16686951	chr20:44747351	CD40	0.047	0.003
cg20119505	chr20:44748928	CD40	0.305	0.015
cg13858266	chr20:44750927	CD40	0.942	0.005
cg06218285	chr20:44751033	CD40	0.127	0.010
cg07222575	chr20:44757985	CD40	0.007	0.012
cg25875565	chr3:139078139	COPB2	0.860	0.005
cg16290059	chr3:139082156	COPB2	0.551	0.003
cg17206056	chr3:139103460	COPB2	0.949	0.012
cg16064982	chr3:139106137	COPB2	0.996	0.015
cg17463863	chr3:139106943	COPB2	0.058	0.004
cg23211065	chr3:139107044	COPB2	0.201	0.009
cg17358529	chr3:139107113	COPB2	0.911	0.000
cg25474358	chr3:139107739	COPB2	0.563	0.009
cg18313110	chr3:139108398	COPB2	0.016	0.001

cg09695652	chr3:139108473	COPB2	0.133	0.016
cg12377709	chr3:139108535	COPB2	0.003	0.001
cg06812153	chr3:139108540	COPB2	< 0.001	0.001
cg16624187	chr3:139108582	COPB2	0.047	0.001
cg00490450	chr3:139108681	COPB2	0.047	0.001
cg15131784	chr3:139108705	COPB2	0.033	0.003
cg09650667	chr3:139108740	COPB2	0.007	0.003
cg16609941	chr3:139108945	COPB2	0.021	0.004
cg14871365	chr3:139108972	COPB2	0.233	0.001
cg07203320	chr3:139108984	COPB2	0.048	0.003
cg14937036	chr3:139109058	COPB2	0.002	0.002
cg23171871	chr5:169063935	DOCK2	0.282	0.001
cg26503653	chr5:169064024	DOCK2	0.099	0.002
cg23598132	chr5:169064038	DOCK2	0.138	0.002
cg21771173	chr5:169064071	DOCK2	0.799	0.004
cg24512013	chr5:169064133	DOCK2	0.230	0.005
cg12800762	chr5:169064311	DOCK2	0.145	0.000
cg08862890	chr5:169064451	DOCK2	0.009	0.002
cg23338503	chr5:169064530	DOCK2	< 0.001	0.007
cg21233722	chr5:169064660	DOCK2	0.001	0.005
cg23184477	chr5:169064897	DOCK2	0.585	0.018
cg16277944	chr5:169068404	DOCK2	0.263	0.004
cg07674170	chr5:169071225	DOCK2	0.002	0.022
cg04780741	chr5:169073733	DOCK2	0.092	0.002
cg24463026	chr5:169074977	DOCK2	0.978	0.049
cg01986936	chr5:169075768	DOCK2	0.499	0.058
cg25365401	chr5:169075924	DOCK2	0.875	0.068
cg13604628	chr5:169076577	DOCK2	0.043	0.067
cg11387564	chr5:169078121	DOCK2	0.001	0.002
cg24125386	chr5:169079813	DOCK2	0.039	0.006
cg14784010	chr5:169083252	DOCK2	0.147	0.014
cg25213601	chr5:169095178	DOCK2	0.043	0.001
cg12627202	chr5:169096691	DOCK2	0.464	0.002
cg05780155	chr5:169098837	DOCK2	0.414	0.002
cg06795948	chr5:169109554	DOCK2	0.554	0.003
cg27028475	chr5:169113219	DOCK2	0.390	0.002
cg02363593	chr5:169123192	DOCK2	0.853	0.001
cg15907287	chr5:169127853	DOCK2	0.063	0.011
cg14693941	chr5:169128366	DOCK2	0.865	0.003
cg25076039	chr5:169128937	DOCK2	0.670	0.008
cg12194596	chr5:169129467	DOCK2	0.451	0.002
cg06740179	chr5:169129904	DOCK2	0.774	0.003
cg17103579	chr5:169130793	DOCK2	0.418	0.003
cg05049849	chr5:169130921	DOCK2	0.123	0.002
cg27063969	chr5:169133966	DOCK2	0.007	0.059
cg04730794	chr5:169144438	DOCK2	0.796	0.095

cg07775790	chr5:169145735	DOCK2	0.620	0.007
cg26982433	chr5:169146570	DOCK2	0.149	0.013
cg12476579	chr5:169172387	DOCK2	0.203	0.011
cg04218566	chr5:169172394	DOCK2	0.435	0.001
cg15540410	chr5:169173591	DOCK2	0.089	0.011
cg16655018	chr5:169180894	DOCK2	0.207	0.001
cg04622239	chr5:169186713	DOCK2	0.258	0.003
cg04188510	chr5:169187197	DOCK2	0.034	0.004
cg08477000	chr5:169187218	DOCK2	0.267	0.004
cg10388716	chr5:169187711	DOCK2	0.759	0.001
cg16512449	chr5:169189786	DOCK2	0.419	0.003
cg01057132	chr5:169190508	DOCK2	0.176	0.004
cg16734895	chr5:169190729	DOCK2	0.977	0.001
cg22405527	chr5:169195272	DOCK2	0.302	0.003
cg16294838	chr5:169195305	DOCK2	0.305	0.006
cg21081460	chr5:169205645	DOCK2	0.055	0.010
cg03727248	chr5:169206480	DOCK2	0.104	0.004
cg05032797	chr5:169206942	DOCK2	0.010	0.013
cg16642321	chr5:169207494	DOCK2	0.951	0.005
cg22244703	chr5:169207793	DOCK2	0.301	0.000
cg02732152	chr5:169217409	DOCK2	0.673	0.006
cg04712944	chr5:169222165	DOCK2	0.203	0.000
cg05008366	chr5:169225981	DOCK2	0.065	0.007
cg16425799	chr5:169230162	DOCK2	1.000	0.080
cg12064462	chr5:169233972	DOCK2	0.450	0.002
cg22004241	chr5:169235259	DOCK2	0.652	0.013
cg23477774	chr5:169235791	DOCK2	0.116	0.009
cg17203784	chr5:169254018	DOCK2	0.981	0.007
cg01045896	chr5:169255151	DOCK2	0.228	0.017
cg21131622	chr5:169259493	DOCK2	0.641	0.011
cg04935901	chr5:169275460	DOCK2	0.752	0.002
cg25429945	chr5:169277675	DOCK2	0.510	0.000
cg19260875	chr5:169283683	DOCK2	0.439	0.019
cg18460239	chr5:169287675	DOCK2	0.166	0.027
cg07442040	chr5:169289365	DOCK2	0.016	0.013
cg25513379	chr5:169308566	DOCK2	0.341	0.001
cg04974097	chr5:169331330	DOCK2	0.513	0.006
cg14881923	chr5:169341945	DOCK2	0.400	0.005
cg06987842	chr5:169350993	DOCK2	0.010	0.012
cg26954905	chr5:169372282	DOCK2	0.920	0.000
cg13691641	chr5:169372529	DOCK2	0.713	0.012
cg02909952	chr5:169404456	DOCK2	0.325	0.001
cg10849016	chr5:169416192	DOCK2	0.009	0.021
cg10677105	chr5:169428710	DOCK2	0.087	0.023
cg01376424	chr5:169434574	DOCK2	0.795	0.007
cg23345454	chr5:169435441	DOCK2	0.318	0.005

cg12472117	chr5:169435729	DOCK2	0.460	0.004
cg25458323	chr5:169467478	DOCK2	0.881	0.002
cg04853571	chr5:169482268	DOCK2	0.540	0.002
cg26794227	chr5:169483131	DOCK2	0.363	0.012
cg18458373	chr5:169483281	DOCK2	0.350	0.012
cg14933717	chr5:169494557	DOCK2	0.269	0.016
cg09175734	chr5:169510081	DOCK2	0.011	0.006
cg09513877	chr5:96111541	ERAP1	0.105	0.001
cg08618451	chr5:96117388	ERAP1	0.496	0.001
cg03624563	chr5:96119711	ERAP1	0.022	0.018
cg10827181	chr5:96120299	ERAP1	0.839	0.056
cg08986950	chr5:96120312	ERAP1	0.454	0.084
cg21479525	chr5:96120323	ERAP1	0.730	0.004
cg21221855	chr5:96122486	ERAP1	0.426	0.007
cg00783142	chr5:96122717	ERAP1	0.017	0.011
cg16020556	chr5:96126443	ERAP1	0.017	0.001
cg16492584	chr5:96139282	ERAP1	0.790	0.002
cg00188032	chr5:96141721	ERAP1	0.277	0.013
cg17335701	chr5:96142712	ERAP1	0.006	0.001
cg05466640	chr5:96143017	ERAP1	0.202	0.001
cg08783793	chr5:96143578	ERAP1	0.059	0.000
cg14161426	chr5:96143592	ERAP1	0.109	0.001
cg23215014	chr5:96143598	ERAP1	0.015	0.000
cg08099655	chr5:96143683	ERAP1	0.001	0.001
cg23602777	chr5:96143688	ERAP1	0.506	0.000
cg09882587	chr5:96143769	ERAP1	0.011	0.000
cg26474110	chr5:96143884	ERAP1	0.761	0.005
cg05612616	chr5:96143890	ERAP1	0.514	0.004
cg05806375	chr5:96144043	ERAP1	0.058	0.002
cg16138854	chr5:96144076	ERAP1	0.001	0.001
cg17522249	chr5:96144101	ERAP1	0.016	0.007
cg12596782	chr5:96144134	ERAP1	0.051	0.003
cg08802853	chr5:96144179	ERAP1	< 0.001	0.001
cg01142811	chr5:96144364	ERAP1	0.055	0.002
cg11789040	chr5:96149885	ERAP1	0.561	0.008
cg11346550	chr5:96149902	ERAP1	0.991	0.029
cg02301737	chr5:96150434	ERAP1	0.864	0.003
cg20661523	chr5:96150454	ERAP1	0.538	0.006
cg22272711	chr5:96150588	ERAP1	0.100	0.002
cg02771117	chr8:11279352	FAM167A	0.096	0.055
cg17014292	chr8:11280085	FAM167A	0.685	0.061
cg07195258	chr8:11281040	FAM167A	0.026	0.002
cg19520497	chr8:11282761	FAM167A	0.625	0.002
cg16105891	chr8:11287161	FAM167A	0.595	0.006
cg05878854	chr8:11288512	FAM167A	0.947	0.005
cg17653522	chr8:11289465	FAM167A	0.616	0.003

cg24094087	chr8:11290839	FAM167A	0.399	0.001
cg18080662	chr8:11290916	FAM167A	0.230	0.000
cg07012910	chr8:11291279	FAM167A	0.239	0.003
cg01153199	chr8:11291623	FAM167A	0.277	0.008
cg06253903	chr8:11295210	FAM167A	0.673	0.006
cg10787818	chr8:11296220	FAM167A	0.148	0.003
cg02621357	chr8:11296980	FAM167A	0.161	0.001
cg10349166	chr8:11301373	FAM167A	0.786	0.024
cg15137355	chr8:11302030	FAM167A	0.865	0.012
cg16824901	chr8:11302243	FAM167A	0.025	0.004
cg15828860	chr8:11302429	FAM167A	0.023	0.000
cg01838251	chr8:11302452	FAM167A	0.240	0.003
cg26274995	chr8:11302983	FAM167A	0.763	0.006
cg06551905	chr8:11303039	FAM167A	0.775	0.026
cg11661318	chr8:11303067	FAM167A	0.005	0.000
cg14356811	chr8:11303794	FAM167A	0.155	0.001
cg11879734	chr8:11304095	FAM167A	0.182	0.012
cg06618371	chr8:11304969	FAM167A	0.345	0.002
cg24453881	chr8:11308943	FAM167A	0.042	0.002
cg17444442	chr8:11310246	FAM167A	0.134	0.005
cg08224773	chr8:11311868	FAM167A	0.885	0.017
cg22022922	chr8:11314934	FAM167A	0.103	0.044
cg24869116	chr8:11315529	FAM167A	0.049	0.068
cg23307893	chr8:11315562	FAM167A	0.322	0.048
cg25973153	chr8:11319359	FAM167A	0.495	0.004
cg11944933	chr8:11320112	FAM167A	0.782	0.004
cg02462837	chr8:11322452	FAM167A	0.782	0.073
cg03803614	chr8:11322979	FAM167A	0.386	0.002
cg01383082	chr8:11323474	FAM167A	0.443	0.007
cg27224195	chr8:11324244	FAM167A	0.010	0.001
cg10220992	chr8:11324327	FAM167A	0.030	0.001
cg08050536	chr8:11324435	FAM167A	0.117	0.000
cg12094006	chr8:11324446	FAM167A	0.071	0.004
cg07392449	chr8:11324666	FAM167A	0.646	0.000
cg12265959	chr8:11324687	FAM167A	0.042	0.007
cg20717059	chr8:11324817	FAM167A	0.020	0.003
cg04048557	chr8:11325201	FAM167A	0.371	0.001
cg05840795	chr8:11325356	FAM167A	0.053	0.001
cg03496850	chr1:161474143	FCGR2A	0.037	0.006
cg25378117	chr1:161474586	FCGR2A	0.113	0.002
cg06350097	chr1:161474970	FCGR2A	0.481	0.016
cg25255913	chr1:161475031	FCGR2A	0.793	0.051
cg04008427	chr1:161475057	FCGR2A	0.585	0.042
cg27470554	chr1:161475240	FCGR2A	0.526	0.042
cg27565811	chr1:161475869	FCGR2A	0.118	0.014
cg13817826	chr1:161475991	FCGR2A	0.201	0.014

cg14831003	chr1:161481799	FCGR2A	0.004	0.018
cg13890597	chr1:161481847	FCGR2A	0.055	0.003
cg14628911	chr1:161482791	FCGR2A	0.461	0.005
cg18073883	chr6:32780861	HLA-DOB	0.656	0.003
cg03530983	chr6:32781171	HLA-DOB	0.318	0.003
cg03083146	chr6:32781235	HLA-DOB	0.104	0.002
cg22016094	chr6:32781261	HLA-DOB	0.124	0.000
cg11239749	chr6:32781307	HLA-DOB	0.066	0.000
cg09889987	chr6:32782136	HLA-DOB	0.857	0.011
cg21454965	chr6:32782405	HLA-DOB	0.599	0.020
cg02010152	chr6:32782617	HLA-DOB	0.645	0.008
cg03477130	chr6:32782796	HLA-DOB	0.497	0.005
cg10294956	chr6:32782845	HLA-DOB	0.347	0.002
cg11469594	chr6:32783046	HLA-DOB	0.984	0.038
cg08905753	chr6:32783347	HLA-DOB	0.670	0.001
cg00409309	chr6:32783551	HLA-DOB	0.144	0.004
cg27246453	chr6:32783881	HLA-DOB	0.480	0.073
cg04576021	chr6:32784255	HLA-DOB	0.329	0.014
cg01530082	chr6:32784327	HLA-DOB	0.045	0.005
cg17369196	chr6:32784487	HLA-DOB	0.031	0.006
cg01493678	chr6:32784825	HLA-DOB	0.267	0.044
cg19301366	chr6:32627845	HLA-DQB1	0.381	0.050
cg14323910	chr6:32628305	HLA-DQB1	0.781	0.026
cg09555323	chr6:32629786	HLA-DQB1	0.891	0.004
cg21493951	chr6:32632338	HLA-DQB1	0.630	0.018
cg24376311	chr6:32632788	HLA-DQB1	0.042	0.001
cg03461499	chr6:32632790	HLA-DQB1	0.469	0.007
cg23967822	chr6:32632961	HLA-DQB1	0.041	0.000
cg03403720	chr6:32632970	HLA-DQB1	0.293	0.004
cg27196273	chr6:32632992	HLA-DQB1	0.356	0.000
cg13047157	chr6:32633114	HLA-DQB1	0.262	0.007
cg24593918	chr6:32633157	HLA-DQB1	0.634	0.006
cg23464743	chr6:32633163	HLA-DQB1	0.337	0.009
cg04696840	chr6:32633354	HLA-DQB1	0.650	0.010
cg13353717	chr6:32634276	HLA-DQB1	0.999	0.027
cg11986643	chr6:32634316	HLA-DQB1	0.906	0.012
cg23336481	chr6:32634344	HLA-DQB1	0.888	0.001
cg05724777	chr6:32634362	HLA-DQB1	0.756	0.005
cg15752756	chr6:32634481	HLA-DQB1	0.613	0.029
cg04090745	chr6:32724369	HLA-DQB2	0.012	0.044
cg17360552	chr6:32725332	HLA-DQB2	0.519	0.005
cg07739876	chr6:32725377	HLA-DQB2	0.861	0.000
cg21013323	chr6:32726567	HLA-DQB2	0.961	0.011
cg24307229	chr6:32726626	HLA-DQB2	0.611	0.003
cg20985082	chr6:32727265	HLA-DQB2	0.784	0.002
cg12296550	chr6:32728862	HLA-DQB2	0.950	0.005

cg06423300	chr6:32729059	HLA-DQB2	0.117	0.003
cg25136631	chr6:32729105	HLA-DQB2	0.776	0.009
cg14255617	chr6:32729118	HLA-DQB2	0.948	0.009
cg07180897	chr6:32729130	HLA-DQB2	0.780	0.004
cg04418355	chr6:32729174	HLA-DQB2	0.626	0.016
cg20645912	chr6:32729274	HLA-DQB2	0.092	0.034
cg13896204	chr6:32729442	HLA-DQB2	0.647	0.013
cg09739413	chr6:32729498	HLA-DQB2	0.871	0.027
cg14441480	chr6:32729500	HLA-DQB2	0.863	0.020
cg02964065	chr6:32729545	HLA-DQB2	0.821	0.039
cg15255946	chr6:32729563	HLA-DQB2	0.919	0.022
cg18026055	chr6:32729596	HLA-DQB2	0.860	0.023
cg18609891	chr6:32729647	HLA-DQB2	0.149	0.009
cg03638120	chr6:32729720	HLA-DQB2	0.883	0.032
cg17475271	chr6:32729741	HLA-DQB2	0.951	0.009
cg04322111	chr6:32729764	HLA-DQB2	0.573	0.011
cg25327122	chr6:32729782	HLA-DQB2	0.731	0.007
cg10218546	chr6:32729823	HLA-DQB2	0.576	0.012
cg19939773	chr6:32729876	HLA-DQB2	0.282	0.002
cg11530659	chr6:32730001	HLA-DQB2	0.770	0.004
cg15615586	chr6:32730040	HLA-DQB2	0.318	0.000
cg10298215	chr6:32730046	HLA-DQB2	0.970	0.004
cg23831898	chr6:32730130	HLA-DQB2	0.097	0.028
cg11505404	chr6:32730243	HLA-DQB2	0.810	0.002
cg21810004	chr6:32730299	HLA-DQB2	0.214	0.000
cg21038932	chr6:32730859	HLA-DQB2	0.263	0.011
cg24892091	chr6:32731127	HLA-DQB2	0.722	0.007
cg23743845	chr6:32732247	HLA-DQB2	0.096	0.003
cg16315262	chr6:32732371	HLA-DQB2	0.019	0.006
cg21566383	chr6:32732764	HLA-DQB2	0.790	0.006
cg06530742	chr19:41223047	ITPKC	0.012	0.001
cg00384577	chr19:41223078	ITPKC	0.457	0.000
cg08162337	chr19:41223262	ITPKC	0.056	0.002
cg16477324	chr19:41223331	ITPKC	0.361	0.001
cg11601297	chr19:41224147	ITPKC	0.018	0.037
cg21869046	chr19:41225005	ITPKC	0.147	0.003
cg27160007	chr19:41225374	ITPKC	0.248	0.013
cg20228862	chr19:41226560	ITPKC	0.672	0.087
cg24099241	chr19:41241624	ITPKC	0.137	0.028
cg01481182	chr19:41245074	ITPKC	0.619	0.026
cg21202522	chr19:41245574	ITPKC	0.252	0.027
cg12879242	chr5:113696517	KCNN2	0.008	0.003
cg14511800	chr5:113696564	KCNN2	0.022	0.003
cg01395723	chr5:113696634	KCNN2	0.015	0.000
cg16430072	chr5:113696863	KCNN2	0.010	0.005
cg26063563	chr5:113697330	KCNN2	0.003	0.001

cg01939336	chr5:113697487	KCNN2	0.003	0.001
cg23615676	chr5:113697632	KCNN2	0.212	0.013
cg05457273	chr5:113697649	KCNN2	0.016	0.001
cg07131533	chr5:113697861	KCNN2	< 0.001	0.002
cg18717628	chr5:113697892	KCNN2	0.002	0.001
cg01894048	chr5:113697895	KCNN2	0.026	0.001
cg18422875	chr5:113697897	KCNN2	0.012	0.000
cg23037611	chr5:113697946	KCNN2	< 0.001	0.007
cg12373617	chr5:113698140	KCNN2	0.003	0.002
cg27403635	chr5:113698291	KCNN2	0.115	0.018
cg24830738	chr5:113698445	KCNN2	0.003	0.008
cg07035165	chr5:113698506	KCNN2	0.001	0.000
cg03264444	chr5:113698664	KCNN2	0.010	0.004
cg26691307	chr5:113698769	KCNN2	0.246	0.013
cg15026311	chr5:113698775	KCNN2	0.015	0.009
cg20930366	chr5:113698847	KCNN2	0.215	0.003
cg24304919	chr5:113699194	KCNN2	< 0.001	0.015
cg04996277	chr5:113699342	KCNN2	0.128	0.019
cg15851052	chr5:113699543	KCNN2	0.011	0.004
cg25194822	chr5:113699604	KCNN2	0.949	0.002
cg11712506	chr5:113699683	KCNN2	0.904	0.001
cg11204913	chr5:113717301	KCNN2	0.608	0.002
cg22140583	chr5:113723647	KCNN2	0.926	0.001
cg11128704	chr5:113725326	KCNN2	0.624	0.011
cg19062099	chr5:113744106	KCNN2	0.417	0.021
cg04843564	chr5:113748800	KCNN2	0.492	0.019
cg23248051	chr5:113753120	KCNN2	0.581	0.001
cg08899474	chr5:113754185	KCNN2	0.405	0.015
cg23216963	chr5:113767813	KCNN2	0.995	0.000
cg25243496	chr5:113770075	KCNN2	0.015	0.022
cg08918534	chr5:113783269	KCNN2	0.056	0.000
cg14558673	chr5:113785032	KCNN2	0.977	0.004
cg01624235	chr5:113785786	KCNN2	0.056	0.007
cg14959824	chr5:113786081	KCNN2	0.033	0.008
cg25486361	chr5:113805552	KCNN2	0.057	0.017
cg17431952	chr5:113806337	KCNN2	0.235	0.033
cg10365469	chr6:168417097	KIF25	0.057	0.002
cg19252565	chr6:168417126	KIF25	0.701	0.001
cg18009322	chr6:168417574	KIF25	0.763	0.007
cg15371806	chr6:168417634	KIF25	0.985	0.002
cg04680494	chr6:168417646	KIF25	0.305	0.001
cg02073839	chr6:168417743	KIF25	0.062	0.003
cg17001430	chr6:168418276	KIF25	0.280	0.006
cg16912129	chr6:168418398	KIF25	0.224	0.006
cg12707343	chr6:168418439	KIF25	0.503	0.002
cg11186282	chr6:168418442	KIF25	0.701	0.001

cg03270619	chr6:168418454	KIF25	0.485	0.003
cg05392263	chr6:168418527	KIF25	0.585	0.018
cg13770417	chr6:168418632	KIF25	0.778	0.009
cg03662475	chr6:168419522	KIF25	0.708	0.003
cg09545394	chr6:168419691	KIF25	0.774	0.001
cg16038269	chr6:168420197	KIF25	0.259	0.005
cg01878651	chr6:168420296	KIF25	0.891	0.006
cg18761763	chr6:168424818	KIF25	0.166	0.006
cg10532440	chr6:168424986	KIF25	0.210	0.001
cg05031152	chr6:168425279	KIF25	0.341	0.004
cg16895216	chr6:168426063	KIF25	0.358	0.005
cg04090349	chr6:168426082	KIF25	0.091	0.008
cg21962025	chr6:168426201	KIF25	0.241	0.006
cg10146514	chr6:168426308	KIF25	0.846	0.005
cg11942669	chr6:168426326	KIF25	0.247	0.006
cg05060686	chr6:168426413	KIF25	0.809	0.019
cg03001276	chr6:168426653	KIF25	0.900	0.002
cg21906947	chr6:168426664	KIF25	0.167	0.002
cg13314057	chr6:168428430	KIF25	0.727	0.002
cg05282968	chr6:168428477	KIF25	0.827	0.001
cg23823000	chr6:168433191	KIF25	0.609	0.006
cg21477075	chr6:168433436	KIF25	0.707	0.012
cg09573356	chr6:168433798	KIF25	0.428	0.004
cg11470213	chr6:168434524	KIF25	0.018	0.010
cg01134139	chr6:168435636	KIF25	0.050	0.009
cg24246628	chr6:168435914	KIF25	0.130	0.013
cg18319852	chr6:168436099	KIF25	0.022	0.006
cg14316629	chr6:168436353	KIF25	0.145	0.042
cg17263665	chr6:168436795	KIF25	0.110	0.011
cg09187944	chr6:168437166	KIF25	0.505	0.010
cg21832294	chr6:168438078	KIF25	0.332	0.005
cg04775383	chr6:168439228	KIF25	0.198	0.003
cg16320173	chr6:168443003	KIF25	0.214	0.002
cg25008524	chr6:168443212	KIF25	0.069	0.004
cg20456465	chr6:168443223	KIF25	0.015	0.001
cg15770012	chr6:168443352	KIF25	0.871	0.006
cg01259258	chr6:168443958	KIF25	0.184	0.013
cg02325163	chr6:168444241	KIF25	0.171	0.009
cg03119088	chr6:168445176	KIF25	0.743	0.008
cg02172773	chr6:168445570	KIF25	0.500	0.005
cg21045072	chr6:168445596	KIF25	0.465	0.004
cg11762760	chr6:168445614	KIF25	0.209	0.003
cg07744273	chr6:31538524	LTA	0.006	0.000
cg20330447	chr6:31539539	LTA	0.078	0.039
cg14441276	chr6:31539735	LTA	0.468	0.073
cg09621572	chr6:31539973	LTA	0.901	0.084

cg14437551	chr6:31539986	LTA	0.895	0.090
cg14597739	chr6:31539998	LTA	0.848	0.065
cg16219283	chr6:31540002	LTA	0.813	0.072
cg21999229	chr6:31540014	LTA	0.672	0.084
cg17169196	chr6:31540026	LTA	0.504	0.084
cg02402436	chr6:31540051	LTA	0.735	0.071
cg09736959	chr6:31540114	LTA	0.765	0.039
cg24216966	chr6:31540121	LTA	0.468	0.060
cg11586857	chr6:31540136	LTA	0.899	0.045
cg22318806	chr6:31540411	LTA	0.607	0.037
cg13815684	chr6:31540440	LTA	0.931	0.036
cg17709873	chr6:31540456	LTA	0.666	0.042
cg26348243	chr6:31540461	LTA	0.536	0.059
cg00501919	chr6:31540750	LTA	0.132	0.054
cg03599224	chr6:31541349	LTA	0.004	0.017
cg21240855	chr6:31541461	LTA	0.257	0.007
cg14910524	chr6:31541948	LTA	0.970	0.011
cg08191289	chr9:139981328	MAN1B1	0.789	0.001
cg10443823	chr9:139981335	MAN1B1	0.005	0.001
cg14604489	chr9:139982149	MAN1B1	0.489	0.001
cg12849357	chr9:139982935	MAN1B1	0.230	0.002
cg22768226	chr9:139983579	MAN1B1	0.024	0.007
cg09911052	chr9:139983593	MAN1B1	0.201	0.003
cg15332579	chr9:139984577	MAN1B1	0.002	0.007
cg13669188	chr9:139984628	MAN1B1	0.069	0.010
cg20376312	chr9:139987318	MAN1B1	0.266	0.002
cg03936816	chr9:139988774	MAN1B1	0.196	0.001
cg04127242	chr9:139990710	MAN1B1	0.231	0.094
cg13614712	chr9:139994787	MAN1B1	0.861	0.001
cg10706861	chr9:139996153	MAN1B1	0.658	0.005
cg17660010	chr9:139997767	MAN1B1	0.821	0.004
cg00000658	chr9:139997924	MAN1B1	0.230	0.003
cg17391845	chr9:139998169	MAN1B1	0.676	0.003
cg14864856	chr9:139998656	MAN1B1	0.561	0.005
cg14244793	chr9:139999038	MAN1B1	0.942	0.008
cg13712518	chr9:140001096	MAN1B1	0.229	0.001
cg09325123	chr9:140001553	MAN1B1	0.067	0.002
cg14578358	chr9:140001730	MAN1B1	0.071	0.010
cg13907059	chr9:140002893	MAN1B1	0.457	0.001
cg14015002	chr9:140003378	MAN1B1	0.082	0.002
cg13592750	chr9:140003531	MAN1B1	0.364	0.006
cg19986979	chr19:41280014	MIA	0.567	0.004
cg24734430	chr19:41280661	MIA	0.521	0.000
cg25152942	chr19:41281080	MIA	0.171	0.002
cg24083702	chr19:41281104	MIA	0.082	0.001
cg19023552	chr19:41281446	MIA	0.015	0.009

cg26783251	chr19:41282678	MIA	0.004	0.008
cg26588045	chr19:41283090	MIA	0.080	0.014
cg01967317	chr3:174576186	NAALADL2	0.913	0.024
cg15622493	chr3:174576569	NAALADL2	0.051	0.034
cg20480368	chr3:174576768	NAALADL2	0.117	0.002
cg04862404	chr3:174577336	NAALADL2	0.316	0.009
cg21145449	chr3:174580620	NAALADL2	0.368	0.001
cg16636878	chr3:174581008	NAALADL2	0.512	0.002
cg05816018	chr3:174609779	NAALADL2	0.628	0.010
cg11632652	chr3:174648902	NAALADL2	0.031	0.007
cg00315255	chr3:174675825	NAALADL2	0.219	0.000
cg19325051	chr3:174691490	NAALADL2	0.592	0.008
cg03525956	chr3:174720287	NAALADL2	0.109	0.010
cg14508517	chr3:174722125	NAALADL2	0.156	0.007
cg11775608	chr3:174759453	NAALADL2	0.111	0.011
cg24575651	chr3:174764477	NAALADL2	0.036	0.013
cg09602541	chr3:174792068	NAALADL2	0.074	0.006
cg25488905	chr3:174838992	NAALADL2	0.036	0.014
cg17095685	chr3:174841409	NAALADL2	0.849	0.006
cg15294279	chr3:174842010	NAALADL2	0.187	0.038
cg16964064	chr3:174844917	NAALADL2	0.013	0.009
cg07601747	chr3:174849894	NAALADL2	0.011	0.014
cg17371709	chr3:174910525	NAALADL2	0.240	0.019
cg06588542	chr3:174911832	NAALADL2	0.969	0.008
cg01295999	chr3:174926334	NAALADL2	0.331	0.003
cg11358672	chr3:174930635	NAALADL2	0.455	0.003
cg01379656	chr3:174970696	NAALADL2	0.316	0.041
cg04784618	chr3:174988389	NAALADL2	0.868	0.001
cg08398040	chr3:175005497	NAALADL2	0.471	0.002
cg01546106	chr3:175052712	NAALADL2	0.839	0.002
cg09890077	chr3:175056652	NAALADL2	0.580	0.006
cg14368211	chr3:175056871	NAALADL2	0.775	0.002
cg09506255	chr3:175060294	NAALADL2	0.011	0.042
cg16953031	chr3:175064540	NAALADL2	0.271	0.006
cg14249533	chr3:175064553	NAALADL2	0.900	0.005
cg12680346	chr3:175065007	NAALADL2	0.819	0.014
cg12817517	chr3:175067212	NAALADL2	0.735	0.007
cg04108951	chr3:175074521	NAALADL2	0.429	0.015
cg14531201	chr3:175104640	NAALADL2	0.313	0.002
cg09620585	chr3:175115181	NAALADL2	0.730	0.004
cg19776822	chr3:175115484	NAALADL2	0.843	0.009
cg05279806	chr3:175128616	NAALADL2	0.820	0.005
cg01150105	chr3:175139160	NAALADL2	0.326	0.005
cg24063405	chr3:175202526	NAALADL2	0.046	0.016
cg16994572	chr3:175204162	NAALADL2	0.588	0.025
cg20051133	chr3:175213208	NAALADL2	0.364	0.010

cg19399866	chr3:175213444	NAALADL2	0.286	0.000
cg14250740	chr3:175244596	NAALADL2	0.007	0.000
cg18290666	chr3:175308718	NAALADL2	0.363	0.007
cg09439444	chr3:175326342	NAALADL2	0.681	0.005
cg17590835	chr3:175326760	NAALADL2	0.332	0.022
cg08076814	chr3:175326825	NAALADL2	0.628	0.017
cg16226037	chr3:175338261	NAALADL2	0.828	0.005
cg22464200	chr3:175338357	NAALADL2	0.993	0.014
cg06296773	chr3:175338547	NAALADL2	0.651	0.004
cg22156300	chr3:175338899	NAALADL2	0.232	0.008
cg09717471	chr3:175338908	NAALADL2	0.503	0.004
cg08995613	chr3:175390218	NAALADL2	0.726	0.001
cg02245488	chr3:175449439	NAALADL2	0.764	0.009
cg19812670	chr3:175461357	NAALADL2	0.762	0.021
cg15672986	chr3:175465989	NAALADL2	0.566	0.015
cg00363658	chr3:175484022	NAALADL2	0.487	0.012
cg09969640	chr3:175484137	NAALADL2	0.013	0.026
cg04998970	chr3:175484712	NAALADL2	0.734	0.009
cg02313589	chr10:21073164	NEBL	0.518	0.005
cg05459252	chr10:21095355	NEBL	0.422	0.000
cg00693776	chr10:21105179	NEBL	0.496	0.006
cg12354307	chr10:21106596	NEBL	0.592	0.008
cg08607662	chr10:21106764	NEBL	0.091	0.009
cg10710988	chr10:21108410	NEBL	0.414	0.000
cg13319203	chr10:21112834	NEBL	0.068	0.009
cg18710812	chr10:21115407	NEBL	0.238	0.006
cg21920074	chr10:21116172	NEBL	0.298	0.001
cg04344600	chr10:21135411	NEBL	0.631	0.005
cg05933215	chr10:21144465	NEBL	0.421	0.008
cg15773746	chr10:21174847	NEBL	0.120	0.001
cg06379027	chr10:21179133	NEBL	0.791	0.002
cg26448411	chr10:21180052	NEBL	0.114	0.011
cg03484402	chr10:21186180	NEBL	0.421	0.019
cg11647420	chr10:21186217	NEBL	0.204	0.002
cg09916763	chr10:21186822	NEBL	0.018	0.047
cg19246545	chr10:21186915	NEBL	0.069	0.026
cg03287579	chr10:21187442	NEBL	0.892	0.002
cg17967996	chr10:21187652	NEBL	0.208	0.014
cg04612110	chr10:21188349	NEBL	0.602	0.012
cg14246963	chr10:21188788	NEBL	0.172	0.001
cg05758203	chr10:21218413	NEBL	0.107	0.016
cg06331574	chr10:21237556	NEBL	0.991	0.003
cg24616879	chr10:21238185	NEBL	0.454	0.019
cg02675443	chr10:21239090	NEBL	0.959	0.002
cg16075753	chr10:21243460	NEBL	0.001	0.001
cg12116749	chr10:21245178	NEBL	0.474	0.002

cg13581027	chr10:21246375	NEBL	0.284	0.003
cg19864972	chr10:21250957	NEBL	0.665	0.000
cg14036154	chr10:21259671	NEBL	0.460	0.001
cg07702770	chr10:21260833	NEBL	0.076	0.030
cg19163398	chr10:21274961	NEBL	0.053	0.010
cg13996738	chr10:21282419	NEBL	0.253	0.005
cg19636344	chr10:21304773	NEBL	0.817	0.004
cg10898402	chr10:21309578	NEBL	0.134	0.005
cg13973039	chr10:21310754	NEBL	0.562	0.004
cg09992949	chr10:21317918	NEBL	0.300	0.005
cg21369919	chr10:21318882	NEBL	0.747	0.004
cg10420290	chr10:21331199	NEBL	0.313	0.003
cg13769883	chr10:21333786	NEBL	0.204	0.006
cg16208523	chr10:21354627	NEBL	0.712	0.005
cg01421400	chr10:21358885	NEBL	0.942	0.008
cg08762432	chr10:21358991	NEBL	0.698	0.009
cg13371840	chr10:21359107	NEBL	0.473	0.022
cg22466310	chr10:21359360	NEBL	0.075	0.015
cg15664767	chr10:21359483	NEBL	0.541	0.011
cg07977230	chr10:21359702	NEBL	0.059	0.013
cg04461058	chr10:21367869	NEBL	0.033	0.006
cg00186224	chr10:21369318	NEBL	0.134	0.022
cg13932637	chr10:21386726	NEBL	0.046	0.008
cg05701192	chr10:21388541	NEBL	0.573	0.020
cg07100050	chr10:21390114	NEBL	0.704	0.029
cg12310370	chr10:21435534	NEBL	0.095	0.004
cg16433788	chr10:21448790	NEBL	0.077	0.012
cg25200453	chr10:21450066	NEBL	0.817	0.003
cg15969702	chr10:21450711	NEBL	0.986	0.008
cg07815856	chr10:21456020	NEBL	0.718	0.005
cg06411879	chr10:21456039	NEBL	0.545	0.023
cg25073701	chr10:21456093	NEBL	0.899	0.010
cg14978741	chr10:21460005	NEBL	0.367	0.001
cg00833352	chr10:21461979	NEBL	0.315	0.004
cg16189346	chr10:21462094	NEBL	0.651	0.001
cg22278087	chr10:21462145	NEBL	0.365	0.008
cg17426273	chr10:21462441	NEBL	0.779	0.004
cg06731125	chr10:21462653	NEBL	0.007	0.003
cg27495903	chr10:21463009	NEBL	< 0.001	0.006
cg11787785	chr10:21463243	NEBL	0.038	0.001
cg01960926	chr10:21463412	NEBL	0.200	0.004
cg23565942	chr10:21463485	NEBL	0.026	0.000
cg18192417	chr10:21463756	NEBL	0.870	0.004
cg24625388	chr10:21463858	NEBL	0.667	0.036
cg09012594	chr10:21464258	NEBL	0.058	0.009
cg13744793	chr10:21464296	NEBL	0.113	0.020

cg13101088	chr2:64320678	PELI1	0.707	0.004
cg12126471	chr2:64322159	PELI1	0.373	0.001
cg14842190	chr2:64327804	PELI1	0.219	0.001
cg20016036	chr2:64332207	PELI1	0.810	0.033
cg24953418	chr2:64336109	PELI1	0.802	0.003
cg21954684	chr2:64341404	PELI1	0.202	0.107
cg08485236	chr2:64343276	PELI1	0.780	0.023
cg22496989	chr2:64348245	PELI1	0.049	0.003
cg00638280	chr2:64349780	PELI1	0.610	0.004
cg12094129	chr2:64356545	PELI1	0.029	0.027
cg16878351	chr2:64360223	PELI1	0.645	0.014
cg18721342	chr2:64361374	PELI1	0.159	0.003
cg17634995	chr2:64362666	PELI1	0.960	0.053
cg19835049	chr2:64365411	PELI1	0.904	0.009
cg11727482	chr2:64368910	PELI1	0.816	0.018
cg22603436	chr2:64370307	PELI1	0.949	0.020
cg26248645	chr2:64370690	PELI1	0.055	0.004
cg22795569	chr2:64371175	PELI1	0.987	0.000
cg17364044	chr2:64371202	PELI1	0.003	0.005
cg16513855	chr2:64371511	PELI1	0.005	0.001
cg22352474	chr2:64371530	PELI1	0.187	0.010
cg09976445	chr2:64371643	PELI1	0.036	0.001
cg00446684	chr2:64371646	PELI1	0.001	0.005
cg14735242	chr2:64371651	PELI1	0.010	0.004
cg20739223	chr2:64371680	PELI1	0.016	0.001
cg02063828	chr2:64371766	PELI1	0.005	0.002
cg14060441	chr2:64371806	PELI1	0.413	0.006
cg01689928	chr2:64371810	PELI1	0.198	0.004
cg18706544	chr2:64371842	PELI1	0.452	0.006
cg08471738	chr2:64373042	PELI1	0.462	0.049
cg00222124	chr6:28248578	PGBD1	0.664	0.072
cg07970287	chr6:28248806	PGBD1	0.521	0.003
cg00836800	chr6:28249106	PGBD1	0.625	0.003
cg10680411	chr6:28249142	PGBD1	0.738	0.002
cg07119389	chr6:28249167	PGBD1	0.227	0.005
cg09002836	chr6:28249205	PGBD1	0.946	0.002
cg11259833	chr6:28249221	PGBD1	0.620	0.001
cg05452243	chr6:28249239	PGBD1	0.967	0.001
cg00017002	chr6:28249251	PGBD1	0.236	0.003
cg09804108	chr6:28249271	PGBD1	0.027	0.000
cg06314658	chr6:28249428	PGBD1	0.002	0.003
cg20029652	chr6:28249545	PGBD1	0.008	0.000
cg11664467	chr6:28249704	PGBD1	0.359	0.001
cg14242556	chr6:28253513	PGBD1	0.267	0.006
cg06579459	chr6:28263113	PGBD1	< 0.001	0.005
cg09853112	chr20:8111582	PLCB1	0.286	0.001

cg09703163	chr20:8111613	PLCB1	0.203	0.000
cg17252911	chr20:8112362	PLCB1	< 0.001	0.002
cg22001510	chr20:8112368	PLCB1	< 0.001	0.000
cg14805807	chr20:8112713	PLCB1	< 0.001	0.003
cg12496245	chr20:8112732	PLCB1	0.007	0.002
cg15528186	chr20:8112749	PLCB1	0.006	0.002
cg23657409	chr20:8112754	PLCB1	0.010	0.001
cg01431260	chr20:8112760	PLCB1	0.001	0.001
cg21844450	chr20:8112956	PLCB1	0.028	0.003
cg17510056	chr20:8112972	PLCB1	0.950	0.001
cg27591117	chr20:8113191	PLCB1	< 0.001	0.001
cg23797439	chr20:8113355	PLCB1	< 0.001	0.002
cg13570585	chr20:8113573	PLCB1	0.032	0.002
cg07008386	chr20:8113630	PLCB1	0.019	0.003
cg09286797	chr20:8116999	PLCB1	0.179	0.006
cg17341800	chr20:8125274	PLCB1	0.798	0.010
cg25556087	chr20:8126124	PLCB1	0.037	0.053
cg13788583	chr20:8132217	PLCB1	0.795	0.013
cg18246521	chr20:8133048	PLCB1	0.885	0.079
cg02524009	chr20:8166645	PLCB1	0.145	0.019
cg16712789	chr20:8189900	PLCB1	0.072	0.001
cg16514642	chr20:8194986	PLCB1	0.072	0.005
cg24732404	chr20:8225365	PLCB1	0.548	0.102
cg13836922	chr20:8227029	PLCB1	0.759	0.002
cg05957477	chr20:8228450	PLCB1	0.379	0.018
cg14326161	chr20:8229198	PLCB1	0.198	0.012
cg21174128	chr20:8229345	PLCB1	0.021	0.001
cg23849443	chr20:8230364	PLCB1	0.246	0.025
cg23434070	chr20:8243123	PLCB1	0.977	0.006
cg22748852	chr20:8255893	PLCB1	0.017	0.021
cg01538049	chr20:8280552	PLCB1	0.249	0.020
cg01212167	chr20:8286191	PLCB1	0.032	0.023
cg10236030	chr20:8286464	PLCB1	0.263	0.023
cg00026239	chr20:8286497	PLCB1	0.956	0.003
cg19579870	chr20:8287236	PLCB1	0.645	0.001
cg11041018	chr20:8290507	PLCB1	0.216	0.008
cg23535804	chr20:8312668	PLCB1	0.039	0.006
cg06033759	chr20:8315973	PLCB1	0.201	0.017
cg05099100	chr20:8341703	PLCB1	0.151	0.024
cg19666328	chr20:8350135	PLCB1	0.455	0.006
cg22384781	chr20:8368897	PLCB1	0.021	0.001
cg21897703	chr20:8374208	PLCB1	0.609	0.015
cg05672540	chr20:8375030	PLCB1	0.996	0.042
cg25731395	chr20:8386100	PLCB1	0.599	0.069
cg18963268	chr20:8404512	PLCB1	0.985	0.002
cg15925374	chr20:8412846	PLCB1	0.994	0.010

cg02415650	chr20:8424307	PLCB1	0.665	0.012
cg15885965	chr20:8428952	PLCB1	0.110	0.002
cg10325053	chr20:8434698	PLCB1	0.720	0.020
cg03104820	chr20:8435207	PLCB1	0.646	0.041
cg18428814	chr20:8445060	PLCB1	0.372	0.021
cg01511124	chr20:8464652	PLCB1	0.143	0.010
cg24237636	chr20:8481650	PLCB1	0.048	0.000
cg08518792	chr20:8504581	PLCB1	0.142	0.011
cg10372668	chr20:8516552	PLCB1	0.290	0.006
cg24477484	chr20:8564675	PLCB1	0.094	0.006
cg22446538	chr20:8570923	PLCB1	0.064	0.019
cg15343777	chr20:8582553	PLCB1	0.159	0.000
cg12188187	chr20:8583715	PLCB1	0.623	0.010
cg08600661	chr20:8584079	PLCB1	0.482	0.001
cg24635752	chr20:8588663	PLCB1	0.145	0.010
cg23459486	chr20:8589150	PLCB1	0.050	0.001
cg13670924	chr20:8589665	PLCB1	0.185	0.002
cg13841889	chr20:8608448	PLCB1	0.305	0.006
cg02970423	chr20:8608498	PLCB1	0.106	0.001
cg21881956	chr20:8608891	PLCB1	0.419	0.004
cg26624712	chr20:8619946	PLCB1	0.262	0.002
cg03698089	chr20:8626828	PLCB1	0.042	0.004
cg10840513	chr20:8630028	PLCB1	0.107	0.006
cg04320983	chr20:8638302	PLCB1	0.042	0.003
cg17847724	chr20:8638883	PLCB1	0.971	0.087
cg04645556	chr20:8638980	PLCB1	0.850	0.095
cg03330328	chr20:8639212	PLCB1	0.284	0.004
cg25659817	chr20:8639249	PLCB1	0.021	0.005
cg19237818	chr20:8639258	PLCB1	0.055	0.008
cg20915818	chr20:8645472	PLCB1	0.819	0.002
cg26359813	chr20:8695977	PLCB1	0.238	0.004
cg07321742	chr20:8696066	PLCB1	0.032	0.027
cg20589039	chr20:8696415	PLCB1	0.253	0.001
cg00404538	chr20:8702075	PLCB1	0.257	0.007
cg06469817	chr20:8703029	PLCB1	0.515	0.001
cg02447937	chr20:8716243	PLCB1	0.546	0.005
cg10068041	chr20:8721036	PLCB1	0.064	0.033
cg03531194	chr20:8733029	PLCB1	0.909	0.003
cg12696295	chr20:8737895	PLCB1	0.217	0.001
cg00995241	chr20:8739884	PLCB1	0.094	0.006
cg07621806	chr20:8770879	PLCB1	0.489	0.010
cg03143830	chr20:8790045	PLCB1	0.992	0.005
cg18291850	chr20:8793439	PLCB1	0.312	0.041
cg06748688	chr20:8810250	PLCB1	0.538	0.004
cg21332848	chr20:8810501	PLCB1	0.979	0.000
cg03181525	chr20:8825072	PLCB1	0.566	0.010

cg27178677	chr20:8834803	PLCB1	0.961	0.008
cg14067086	chr20:8861389	PLCB1	0.465	0.003
cg04794674	chr20:9048614	PLCB4	0.742	0.005
cg01139925	chr20:9048845	PLCB4	0.438	0.018
cg12895255	chr20:9049669	PLCB4	0.098	0.001
cg06529883	chr20:9049686	PLCB4	0.128	0.001
cg23685416	chr20:9049689	PLCB4	0.241	0.000
cg24231038	chr20:9049691	PLCB4	0.301	0.002
cg27031864	chr20:9049819	PLCB4	0.010	0.004
cg08576827	chr20:9075493	PLCB4	0.123	0.006
cg20484832	chr20:9075810	PLCB4	0.434	0.000
cg03100801	chr20:9075962	PLCB4	0.208	0.000
cg24736099	chr20:9077730	PLCB4	0.041	0.006
cg19492010	chr20:9100498	PLCB4	0.560	0.047
cg24351677	chr20:9128188	PLCB4	0.719	0.011
cg13456056	chr20:9129940	PLCB4	0.230	0.026
cg09143713	chr20:9141615	PLCB4	0.731	0.043
cg03832855	chr20:9146980	PLCB4	0.815	0.004
cg03973404	chr20:9149533	PLCB4	0.295	0.019
cg11758280	chr20:9153867	PLCB4	0.644	0.043
cg16480239	chr20:9160245	PLCB4	0.096	0.040
cg19943221	chr20:9181020	PLCB4	0.981	0.038
cg17742409	chr20:9181105	PLCB4	0.830	0.029
cg23898322	chr20:9191844	PLCB4	0.063	0.027
cg12164029	chr20:9195836	PLCB4	0.307	0.010
cg25279093	chr20:9196445	PLCB4	0.120	0.005
cg15168017	chr20:9196718	PLCB4	0.024	0.014
cg26806367	chr20:9197593	PLCB4	0.196	0.000
cg08886490	chr20:9197927	PLCB4	0.100	0.037
cg03225601	chr20:9198009	PLCB4	0.162	0.014
cg04037197	chr20:9198061	PLCB4	0.066	0.033
cg06899578	chr20:9198363	PLCB4	0.890	0.004
cg13312062	chr20:9199290	PLCB4	0.997	0.008
cg21296708	chr20:9216201	PLCB4	0.698	0.004
cg24823920	chr20:9287042	PLCB4	0.012	0.009
cg25295273	chr20:9287061	PLCB4	0.575	0.008
cg19904783	chr20:9287445	PLCB4	0.410	0.029
cg09222486	chr20:9288096	PLCB4	0.254	0.003
cg25947253	chr20:9288423	PLCB4	0.011	0.006
cg26238857	chr20:9304477	PLCB4	0.256	0.003
cg17781668	chr20:9334016	PLCB4	0.342	0.016
cg22989419	chr20:9340396	PLCB4	0.356	0.003
ch.20.209864F	chr20:9344219	PLCB4	0.075	0.005
cg15981619	chr20:9345648	PLCB4	0.086	0.008
cg06642126	chr20:9374112	PLCB4	0.991	0.060
cg10575488	chr20:9378659	PLCB4	0.762	0.010

cg00821851	chr20:9384559	PLCB4	0.022	0.012
cg23939000	chr20:9389788	PLCB4	0.115	0.009
cg08700482	chr20:9393279	PLCB4	0.386	0.002
cg12416274	chr20:9423494	PLCB4	0.643	0.000
cg19238141	chr20:9441312	PLCB4	0.831	0.002
cg16864832	chr20:9449312	PLCB4	0.724	0.001
cg19416290	chr20:9459569	PLCB4	0.528	0.008
cg18870258	chr20:9460935	PLCB4	0.097	0.006
cg12124543	chr11:48001298	PTPRJ	0.587	0.028
cg00394658	chr11:48001669	PTPRJ	0.858	0.044
cg13646917	chr11:48001761	PTPRJ	0.936	0.021
cg19851384	chr11:48001835	PTPRJ	0.058	0.000
cg06890484	chr11:48001940	PTPRJ	< 0.001	0.000
cg02616975	chr11:48001954	PTPRJ	< 0.001	0.002
cg10457846	chr11:48002566	PTPRJ	0.002	0.001
cg06780358	chr11:48003065	PTPRJ	0.188	0.001
cg14042131	chr11:48003169	PTPRJ	0.022	0.004
cg23026023	chr11:48005149	PTPRJ	0.842	0.039
cg17428739	chr11:48007978	PTPRJ	0.726	0.005
cg11185765	chr11:48009578	PTPRJ	0.848	0.054
cg16357878	chr11:48009653	PTPRJ	0.961	0.076
cg16549335	chr11:48011604	PTPRJ	0.010	0.000
cg02980360	chr11:48012728	PTPRJ	0.147	0.016
cg04222440	chr11:48012950	PTPRJ	0.305	0.018
cg08025630	chr11:48016827	PTPRJ	0.193	0.006
cg09410691	chr11:48018661	PTPRJ	0.824	0.002
cg16027310	chr11:48019123	PTPRJ	0.646	0.001
cg11545521	chr11:48019467	PTPRJ	0.166	0.015
cg01000627	chr11:48023430	PTPRJ	0.159	0.085
cg22111167	chr11:48026889	PTPRJ	0.220	0.015
cg15813528	chr11:48028299	PTPRJ	0.004	0.066
cg06566905	chr11:48029442	PTPRJ	0.909	0.050
cg20563072	chr11:48030718	PTPRJ	0.018	0.083
cg00695187	chr11:48032704	PTPRJ	0.006	0.037
cg11468953	chr11:48034953	PTPRJ	0.711	0.021
cg07401324	chr11:48036852	PTPRJ	0.776	0.083
cg19205376	chr11:48037488	PTPRJ	0.949	0.070
cg03278516	chr11:48037855	PTPRJ	0.031	0.035
cg21620736	chr11:48037919	PTPRJ	0.241	0.008
cg19268720	chr11:48038449	PTPRJ	0.818	0.015
cg10238692	chr11:48039247	PTPRJ	0.003	0.006
cg22976120	chr11:48040772	PTPRJ	0.496	0.018
cg19067682	chr11:48041043	PTPRJ	0.788	0.025
cg01372366	chr11:48041783	PTPRJ	0.585	0.050
cg01519847	chr11:48042101	PTPRJ	0.908	0.040
cg00120747	chr11:48046162	PTPRJ	0.883	0.002

cg24404318	chr11:48053509	PTPRJ	0.920	0.068
cg15300753	chr11:48057861	PTPRJ	0.058	0.002
cg05474665	chr11:48062997	PTPRJ	0.676	0.002
cg14802758	chr11:48063916	PTPRJ	0.126	0.012
cg08320911	chr11:48065094	PTPRJ	0.707	0.029
cg02956479	chr11:48065711	PTPRJ	0.346	0.001
cg14997711	chr11:48065851	PTPRJ	0.810	0.005
cg23656969	chr11:48066198	PTPRJ	0.003	0.023
cg10951214	chr11:48069893	PTPRJ	0.244	0.006
cg08383438	chr11:48071665	PTPRJ	0.041	0.003
cg02133150	chr11:48072560	PTPRJ	0.386	0.011
cg21032823	chr11:48073477	PTPRJ	0.133	0.001
cg12057634	chr11:48073699	PTPRJ	0.114	0.008
cg26468891	chr11:48077221	PTPRJ	0.002	0.019
cg14815299	chr11:48077914	PTPRJ	0.325	0.023
cg23646614	chr11:48083100	PTPRJ	0.988	0.060
cg26197915	chr11:48085137	PTPRJ	0.922	0.054
cg00325914	chr11:48088520	PTPRJ	0.699	0.024
cg27008438	chr11:48094786	PTPRJ	0.793	0.005
cg22985571	chr11:48096309	PTPRJ	0.002	0.043
cg00526281	chr11:48101308	PTPRJ	0.280	0.018
cg03808749	chr11:48102652	PTPRJ	0.275	0.007
cg02372723	chr11:48103048	PTPRJ	0.999	0.079
cg21687008	chr11:48106202	PTPRJ	0.012	0.004
cg01456695	chr11:48108785	PTPRJ	0.086	0.006
cg07143125	chr11:48110045	PTPRJ	0.931	0.056
cg19325637	chr11:48112253	PTPRJ	0.977	0.001
cg06298729	chr11:48118041	PTPRJ	0.776	0.004
cg24320937	chr11:48118658	PTPRJ	0.278	0.003
cg27663642	chr11:48119692	PTPRJ	0.481	0.026
cg18477635	chr11:48122845	PTPRJ	0.239	0.006
ch.11.1014025R	chr11:48124683	PTPRJ	0.898	0.010
cg21141258	chr11:48125522	PTPRJ	0.044	0.010
cg01106056	chr11:48126141	PTPRJ	0.564	0.021
cg18725092	chr11:48127161	PTPRJ	0.004	0.014
cg14287039	chr11:48128164	PTPRJ	0.671	0.021
cg04515608	chr11:48129067	PTPRJ	0.416	0.036
cg09173617	chr11:48131664	PTPRJ	0.147	0.001
cg12730832	chr11:48145766	PTPRJ	0.692	0.006
cg05165365	chr11:48151361	PTPRJ	0.527	0.135
cg20667822	chr11:48153832	PTPRJ	0.072	0.022
cg17335440	chr11:48161380	PTPRJ	0.907	0.006
cg23547656	chr11:48161861	PTPRJ	0.104	0.020
cg07999939	chr11:48163116	PTPRJ	0.093	0.006
cg15418976	chr11:48165989	PTPRJ	0.196	0.003
cg02063420	chr11:48166513	PTPRJ	0.240	0.013

cg07012578	chr11:48168673	PTPRJ	0.395	0.011
cg21469197	chr11:48175868	PTPRJ	0.217	0.000
cg02299195	chr11:48185069	PTPRJ	0.427	0.003
cg05982345	chr11:48191068	PTPRJ	0.257	0.007
cg09960747	chr19:41283407	RAB4B	0.122	0.000
cg02975986	chr19:41283506	RAB4B	0.023	0.002
cg06439809	chr19:41283578	RAB4B	0.810	0.001
cg24958765	chr19:41283667	RAB4B	0.269	0.001
cg07515241	chr19:41284000	RAB4B	0.142	0.003
cg20611850	chr19:41284006	RAB4B	0.055	0.008
cg10787145	chr19:41284032	RAB4B	0.033	0.000
cg22032540	chr19:41284057	RAB4B	0.247	0.001
cg15535636	chr19:41284070	RAB4B	0.090	0.002
cg13332130	chr19:41284234	RAB4B	< 0.001	0.001
cg20951968	chr19:41284348	RAB4B	0.158	0.002
cg16853712	chr19:41286530	RAB4B	0.041	0.002
cg24062310	chr19:41289994	RAB4B	0.009	0.004
cg00831560	chr19:41290997	RAB4B	0.044	0.006
cg25621094	chr19:41292592	RAB4B	0.189	0.001
cg04671526	chr19:41292694	RAB4B	0.224	0.000
cg19873111	chr19:41294244	RAB4B	0.035	0.007
cg26741226	chr19:41295838	RAB4B	0.542	0.000
cg05301494	chr19:41302185	RAB4B	0.578	0.001
cg13355779	chr20:35806326	RPN2	0.089	0.029
cg16553051	chr20:35806496	RPN2	0.038	0.020
cg15627502	chr20:35806795	RPN2	0.134	0.002
cg12895429	chr20:35806810	RPN2	0.446	0.003
cg10873891	chr20:35806913	RPN2	0.997	0.004
cg02930037	chr20:35807377	RPN2	0.048	0.001
cg16176383	chr20:35807410	RPN2	0.382	0.000
cg05533115	chr20:35807416	RPN2	0.071	0.001
cg08120289	chr20:35807421	RPN2	0.204	0.000
cg13946860	chr20:35807510	RPN2	0.001	0.001
cg16623656	chr20:35807575	RPN2	0.001	0.009
cg13173903	chr20:35807784	RPN2	0.011	0.001
cg20079898	chr20:35807905	RPN2	0.031	0.004
cg01140293	chr20:35807911	RPN2	0.007	0.006
cg15389461	chr20:35818328	RPN2	0.114	0.007
cg18016202	chr20:35824027	RPN2	0.348	0.004
cg24469719	chr20:35833182	RPN2	0.409	0.001
cg18549386	chr20:35833196	RPN2	0.078	0.004
cg06889272	chr20:35833202	RPN2	0.117	0.007
cg16831894	chr20:35833240	RPN2	0.145	0.002
cg08531864	chr20:35841852	RPN2	0.068	0.019
cg16605933	chr20:35842190	RPN2	0.081	0.009
cg19233297	chr20:35851412	RPN2	0.639	0.001

cg19632636	chr20:35856276	RPN2	0.072	0.005
cg20067831	chr20:35856858	RPN2	0.314	0.002
cg05623559	chr20:35857024	RPN2	0.473	0.012
cg02058787	chr20:35857341	RPN2	0.436	0.004
cg24308162	chr20:35862530	RPN2	0.782	0.013
cg24377945	chr20:35867528	RPN2	0.441	0.009
cg09890930	chr12:56149396	SARNP	0.052	0.004
cg05511733	chr12:56151190	SARNP	0.727	0.003
cg10101707	chr12:56154315	SARNP	0.113	0.022
cg10267968	chr12:56155142	SARNP	0.046	0.017
cg14182058	chr12:56156904	SARNP	0.764	0.001
cg25366834	chr12:56157878	SARNP	0.842	0.004
cg12292972	chr12:56158737	SARNP	0.140	0.005
cg23689080	chr12:56159704	SARNP	0.039	0.002
cg13458087	chr12:56165672	SARNP	0.808	0.007
cg03694302	chr12:56191228	SARNP	0.847	0.013
cg15945863	chr12:56191502	SARNP	0.349	0.002
cg12799537	chr12:56207909	SARNP	0.150	0.001
cg23184344	chr12:56211634	SARNP	0.501	0.000
cg16660717	chr12:56211781	SARNP	0.730	0.000
cg05584675	chr12:56211824	SARNP	0.095	0.001
cg18710288	chr12:56211992	SARNP	0.382	0.001
cg21995174	chr12:56212070	SARNP	0.816	0.016
cg06990019	chr12:56212084	SARNP	0.748	0.003
cg08799692	chr12:56212243	SARNP	0.069	0.000
cg22225546	chr12:56212269	SARNP	0.034	0.004
cg03744975	chr12:56212418	SARNP	0.210	0.012
cg06904727	chr12:56212550	SARNP	0.042	0.039
cg08214038	chr22:24666470	SPECC1L	0.003	0.003
cg10973068	chr22:24666818	SPECC1L	0.036	0.012
cg11978118	chr22:24669106	SPECC1L	0.251	0.060
cg19921814	chr22:24670119	SPECC1L	0.192	0.027
cg22466032	chr22:24681310	SPECC1L	0.811	0.052
cg21636253	chr22:24685775	SPECC1L	0.023	0.035
cg24173387	chr22:24688117	SPECC1L	0.977	0.006
cg15942313	chr22:24737278	SPECC1L	0.366	0.002
cg04248621	chr22:24737382	SPECC1L	0.077	0.021
cg00127028	chr22:24737496	SPECC1L	0.105	0.001
cg10763737	chr22:24737582	SPECC1L	0.119	0.002
cg13752382	chr22:24737646	SPECC1L	0.841	0.000
cg20412029	chr22:24737906	SPECC1L	0.234	0.001
cg12521217	chr22:24737946	SPECC1L	0.003	0.003
cg25580335	chr22:24738240	SPECC1L	0.765	0.025
cg15548624	chr22:24755907	SPECC1L	0.994	0.000
cg16230918	chr22:24759060	SPECC1L	0.062	0.003
cg26353903	chr5:134784604	TIFAB	0.599	0.007

cg12481536	chr5:134784606	TIFAB	0.455	0.006
cg16339915	chr5:134785391	TIFAB	0.019	0.006
cg02122200	chr5:134785604	TIFAB	0.293	0.008
cg09288778	chr5:134786107	TIFAB	0.410	0.030
cg12738534	chr5:134786112	TIFAB	0.096	0.039
cg16750772	chr5:134786269	TIFAB	0.003	0.038
cg09875213	chr5:134786596	TIFAB	0.081	0.031
cg16176675	chr5:134786942	TIFAB	0.473	0.028
cg03541914	chr5:134787465	TIFAB	0.100	0.009
cg22979041	chr5:134787907	TIFAB	0.922	0.012
cg03510194	chr5:134788007	TIFAB	0.834	0.039
cg18957078	chr5:134788128	TIFAB	0.449	0.027
cg00766507	chr5:134788607	TIFAB	0.133	0.005
cg13691824	chr5:134788649	TIFAB	0.034	0.020
cg06324419	chr5:134788694	TIFAB	0.400	0.001
cg19949797	chr5:134789115	TIFAB	0.804	0.002
cg01288904	chr8:80947440	TPD52	0.189	0.003
cg23408678	chr8:80955164	TPD52	0.885	0.005
cg00370891	chr8:80960387	TPD52	0.121	0.002
cg06350472	chr8:80963803	TPD52	0.414	0.008
cg19935724	chr8:80964035	TPD52	0.790	0.004
cg07930159	chr8:80964882	TPD52	0.702	0.027
cg19925215	chr8:80964918	TPD52	0.442	0.038
cg22887915	chr8:80965595	TPD52	0.516	0.003
cg07132557	chr8:80967149	TPD52	0.522	0.050
cg26384678	chr8:80977008	TPD52	0.318	0.022
cg22692608	chr8:80978343	TPD52	0.773	0.031
cg05084750	chr8:80979548	TPD52	0.279	0.005
cg18676805	chr8:80990403	TPD52	0.556	0.004
cg06634867	chr8:80990459	TPD52	0.386	0.003
cg02549588	chr8:80992699	TPD52	0.887	0.001
cg16773115	chr8:80992750	TPD52	0.600	0.001
cg07892425	chr8:80992982	TPD52	0.101	0.013
cg17540250	chr8:80993543	TPD52	0.998	0.011
cg13155823	chr8:80993721	TPD52	0.794	0.012
cg26428748	chr8:80993726	TPD52	0.080	0.019
cg14507357	chr8:80994056	TPD52	0.230	0.008
cg04963948	chr8:80997426	TPD52	0.008	0.004
cg27415032	chr8:80997524	TPD52	0.463	0.007
cg04068975	chr8:81001528	TPD52	0.336	0.015
cg15628415	chr8:81003747	TPD52	0.026	0.003
cg15183852	chr8:81009091	TPD52	0.036	0.024
cg11138606	chr8:81013230	TPD52	0.177	0.002
cg21595409	chr8:81014522	TPD52	0.214	0.012
cg09737525	chr8:81014736	TPD52	0.348	0.046
cg10933148	chr8:81015199	TPD52	0.437	0.005

cg11669824	chr8:81020215	TPD52	0.524	0.006
cg03817394	chr8:81033597	TPD52	0.368	0.005
cg16976808	chr8:81033743	TPD52	0.158	0.012
cg13009608	chr8:81034420	TPD52	0.025	0.001
cg01490091	chr8:81035207	TPD52	0.057	0.010
cg04714581	chr8:81047040	TPD52	0.988	0.078
cg14075772	chr8:81049968	TPD52	0.571	0.004
cg14587889	chr8:81051024	TPD52	0.418	0.033
cg04384543	chr8:81053048	TPD52	0.498	0.016
cg20615753	chr8:81053284	TPD52	0.398	0.033
cg21582824	chr8:81054395	TPD52	0.951	0.021
cg16846740	chr8:81055067	TPD52	0.413	0.006
cg03193834	chr8:81057597	TPD52	0.791	0.003
cg04112878	chr8:81059932	TPD52	0.160	0.007
cg22904437	chr8:81064304	TPD52	0.359	0.027
cg12951110	chr8:81065540	TPD52	0.338	0.034
cg11217679	chr8:81070832	TPD52	0.739	0.015
cg23342367	chr8:81077480	TPD52	0.756	0.099
cg09540738	chr8:81080281	TPD52	0.615	0.083
cg21250232	chr8:81082895	TPD52	0.117	0.006
cg23295127	chr8:81083339	TPD52	0.025	0.003
cg07587588	chr8:81083356	TPD52	0.125	0.006
cg16008931	chr8:81083431	TPD52	0.165	0.008
cg16638571	chr8:81083534	TPD52	< 0.001	0.002
cg01341301	chr8:81083577	TPD52	0.001	0.001
cg04341084	chr8:81083671	TPD52	0.001	0.001
cg22273042	chr8:81083829	TPD52	0.026	0.006
cg02399584	chr8:81083852	TPD52	0.007	0.001
cg19420321	chr8:81084048	TPD52	0.040	0.016
cg18459342	chr8:81084056	TPD52	0.094	0.017
cg09619309	chr8:81084440	TPD52	0.535	0.002
cg16391871	chr8:81084488	TPD52	0.149	0.002
cg12332167	chr8:81084554	TPD52	0.008	0.004
cg16496585	chr8:81084705	TPD52	0.026	0.006
cg19319780	chr8:81084882	TPD52	0.032	0.002
cg24729352	chr13:19747959	TUBA3C	0.754	0.006
cg24828246	chr13:19749816	TUBA3C	0.599	0.003
cg19925741	chr13:19751465	TUBA3C	0.271	0.005
cg08756887	chr13:19754434	TUBA3C	0.878	0.001
cg10802118	chr13:19754926	TUBA3C	0.780	0.003
cg11511819	chr13:19755670	TUBA3C	0.487	0.002
cg22675150	chr13:19755900	TUBA3C	0.070	0.012
cg04131456	chr13:19755916	TUBA3C	0.796	0.001
cg06437862	chr13:19756096	TUBA3C	0.876	0.008
cg05006718	chr13:19756122	TUBA3C	0.837	0.007
cg15615022	chr13:19756254	TUBA3C	0.685	0.022

cg22274414	chr13:19756318	TUBA3C	0.964	0.009
cg14392476	chr13:19756329	TUBA3C	0.305	0.007
cg04269057	chr10:72971005	UNC5B	0.798	0.005
cg16786583	chr10:72971759	UNC5B	0.641	0.010
cg07477077	chr10:72971873	UNC5B	0.521	0.019
cg05871136	chr10:72971961	UNC5B	0.275	0.014
cg08342721	chr10:72972076	UNC5B	0.005	0.001
cg15929693	chr10:72972132	UNC5B	0.176	0.000
cg09470230	chr10:72972137	UNC5B	0.030	0.000
cg20254483	chr10:72972204	UNC5B	0.353	0.000
cg26450586	chr10:72972207	UNC5B	0.038	0.000
cg18858343	chr10:72972901	UNC5B	0.001	0.000
cg14042687	chr10:72973260	UNC5B	0.099	0.001
cg08905239	chr10:72973765	UNC5B	0.883	0.028
cg04177244	chr10:72973894	UNC5B	0.945	0.000
cg07462866	chr10:72974629	UNC5B	0.043	0.014
cg25343399	chr10:72974694	UNC5B	0.058	0.002
cg12873350	chr10:72976676	UNC5B	0.276	0.019
cg18579564	chr10:72977378	UNC5B	0.058	0.006
cg23967873	chr10:72977537	UNC5B	0.055	0.011
cg14957186	chr10:72977773	UNC5B	0.234	0.002
cg18389931	chr10:72978724	UNC5B	0.034	0.014
cg24591279	chr10:72982627	UNC5B	0.010	0.002
cg26328326	chr10:72983319	UNC5B	0.280	0.016
cg10319211	chr10:72983571	UNC5B	0.129	0.007
cg22238397	chr10:72989211	UNC5B	0.338	0.012
cg11390659	chr10:72990169	UNC5B	0.011	0.050
cg17220314	chr10:72992083	UNC5B	0.034	0.031
cg04478997	chr10:72992359	UNC5B	0.065	0.013
cg19673155	chr10:72995181	UNC5B	0.541	0.020
cg12481560	chr10:72997126	UNC5B	0.550	0.016
cg08206047	chr10:72997755	UNC5B	0.409	0.001
cg27135218	chr10:73000505	UNC5B	0.931	0.039
cg10893388	chr10:73001413	UNC5B	0.481	0.020
cg13185473	chr10:73003572	UNC5B	0.006	0.022
cg09539094	chr10:73003617	UNC5B	0.282	0.001
cg26152017	chr10:73004558	UNC5B	0.749	0.031
cg24839386	chr10:73004576	UNC5B	0.792	0.029
cg08913389	chr10:73005575	UNC5B	0.038	0.012
cg11201708	chr10:73010487	UNC5B	0.868	0.099
cg19343212	chr10:73012347	UNC5B	0.276	0.015
cg02440719	chr10:73013749	UNC5B	0.008	0.010
cg21732487	chr10:73013792	UNC5B	0.097	0.008
cg13094293	chr10:73013827	UNC5B	0.052	0.003
cg04904026	chr10:73015078	UNC5B	0.720	0.007
cg17668756	chr10:73020218	UNC5B	0.053	0.007

cg16759416	chr10:73022276	UNC5B	0.782	0.000
cg22567444	chr10:73023373	UNC5B	0.932	0.002
cg02378006	chr10:73026288	UNC5B	0.373	0.014
cg09903766	chr10:73027051	UNC5B	0.216	0.013
cg27413180	chr10:73028540	UNC5B	0.010	0.017
cg20194733	chr10:73033477	UNC5B	0.100	0.012
cg19808533	chr10:73035408	UNC5B	0.552	0.012
cg02978938	chr10:73036974	UNC5B	0.129	0.003
cg17477835	chr10:73038012	UNC5B	0.015	0.033
cg18317005	chr10:73039285	UNC5B	0.890	0.019
ch.10.1515233F	chr10:73039321	UNC5B	0.191	0.002
cg16180595	chr10:73040496	UNC5B	0.019	0.006
cg13618472	chr10:73042210	UNC5B	0.067	0.008
cg16301256	chr10:73045356	UNC5B	0.044	0.011
cg17551083	chr10:73048291	UNC5B	0.277	0.004
cg13824565	chr10:73048387	UNC5B	0.740	0.008
cg08814830	chr10:73049277	UNC5B	0.083	0.003
cg16817550	chr10:73051053	UNC5B	0.005	0.006
cg25514423	chr10:73052493	UNC5B	0.616	0.003
cg23622162	chr10:73059249	UNC5B	0.942	0.041
cg17132554	chr10:73061004	UNC5B	0.906	0.003
cg05227773	chr16:72817808	ZFHX3	0.081	0.012
cg03045067	chr16:72819254	ZFHX3	0.525	0.005
cg09761690	chr16:72820681	ZFHX3	0.324	0.004
cg05543909	chr16:72820921	ZFHX3	0.805	0.002
cg08961664	chr16:72821141	ZFHX3	0.832	0.004
cg02246055	chr16:72821353	ZFHX3	0.015	0.001
cg08512490	chr16:72821498	ZFHX3	< 0.001	0.002
cg16630989	chr16:72821566	ZFHX3	0.049	0.003
cg04814450	chr16:72821665	ZFHX3	0.031	0.001
cg01229752	chr16:72822033	ZFHX3	0.900	0.000
cg19594635	chr16:72822362	ZFHX3	0.976	0.053
cg07667790	chr16:72823692	ZFHX3	0.519	0.000
cg00075615	chr16:72827803	ZFHX3	0.155	0.001
cg03382246	chr16:72827884	ZFHX3	0.089	0.009
cg04994202	chr16:72828024	ZFHX3	0.362	0.004
cg08452002	chr16:72829368	ZFHX3	0.030	0.002
cg03224276	chr16:72829831	ZFHX3	0.189	0.002
cg16372869	chr16:72829901	ZFHX3	0.099	0.001
cg07005580	chr16:72830034	ZFHX3	0.170	0.000
cg02372821	chr16:72830233	ZFHX3	0.971	0.005
cg04101363	chr16:72830284	ZFHX3	0.730	0.002
cg18315961	chr16:72839653	ZFHX3	0.117	0.005
cg03752425	chr16:72840504	ZFHX3	0.437	0.000
cg12993369	chr16:72840594	ZFHX3	0.589	0.002
cg01312273	chr16:72852527	ZFHX3	0.071	0.024

cg20053399	chr16:72853514	ZFHX3	0.333	0.011
cg27273821	chr16:72854773	ZFHX3	0.522	0.003
cg22253600	chr16:72856057	ZFHX3	0.316	0.010
cg23410343	chr16:72856353	ZFHX3	0.285	0.003
cg14881826	chr16:72856936	ZFHX3	0.316	0.003
cg07696190	chr16:72857124	ZFHX3	0.737	0.008
cg08963581	chr16:72863301	ZFHX3	0.150	0.016
cg07457984	chr16:72867265	ZFHX3	0.397	0.005
cg08508319	chr16:72870964	ZFHX3	0.871	0.007
cg20915517	chr16:72871902	ZFHX3	0.273	0.002
cg00908120	chr16:72872925	ZFHX3	0.292	0.001
cg03379631	chr16:72873149	ZFHX3	0.588	0.002
cg25038295	chr16:72874571	ZFHX3	0.588	0.015
cg05946791	chr16:72878556	ZFHX3	0.023	0.003
cg05969150	chr16:72882495	ZFHX3	0.094	0.022
cg27182230	chr16:72882824	ZFHX3	0.602	0.025
cg11615523	chr16:72883588	ZFHX3	0.283	0.002
cg16397028	chr16:72884581	ZFHX3	0.255	0.013
cg24651536	chr16:72888448	ZFHX3	0.311	0.041
cg05723075	chr16:72890498	ZFHX3	0.516	0.009
cg10472320	chr16:72892765	ZFHX3	0.311	0.004
cg09223396	chr16:72893924	ZFHX3	0.430	0.000
cg07213145	chr16:72893945	ZFHX3	0.234	0.003
cg06301789	chr16:72901431	ZFHX3	0.850	0.004
cg08968069	chr16:72903003	ZFHX3	0.523	0.009
cg00390483	chr16:72907786	ZFHX3	0.243	0.003
cg20539444	chr16:72909135	ZFHX3	0.047	0.012
cg21815992	chr16:72910199	ZFHX3	0.847	0.039
cg15959611	chr16:72911371	ZFHX3	0.711	0.021
cg09293559	chr16:72911729	ZFHX3	0.910	0.004
cg08939976	chr16:72912494	ZFHX3	0.974	0.065
cg08858926	chr16:72918832	ZFHX3	0.526	0.020
cg24058010	chr16:72923910	ZFHX3	0.314	0.007
cg26731575	chr16:72929007	ZFHX3	0.030	0.003
cg01779559	chr16:72930084	ZFHX3	0.097	0.002
cg09681300	chr16:72930200	ZFHX3	0.563	0.002
cg05568868	chr16:72930479	ZFHX3	0.947	0.001
cg14312104	chr16:72938620	ZFHX3	0.201	0.003
cg18636647	chr16:72939842	ZFHX3	0.153	0.016
cg24301278	chr16:72942625	ZFHX3	0.155	0.015
cg08645153	chr16:72942661	ZFHX3	0.877	0.006
cg07203886	chr16:72945431	ZFHX3	0.149	0.003
cg12190404	chr16:72948114	ZFHX3	0.863	0.000
cg03888640	chr16:72955161	ZFHX3	0.053	0.018
cg10104480	chr16:72955250	ZFHX3	0.064	0.010
cg05918327	chr16:72955295	ZFHX3	0.125	0.010

cg10775849	chr16:72957911	ZFHX3	0.159	0.002
cg09837951	chr16:72958530	ZFHX3	0.159	0.001
cg23625230	chr16:72958938	ZFHX3	0.175	0.022
cg03138635	chr16:72960528	ZFHX3	0.104	0.008
cg07434438	chr16:72961899	ZFHX3	0.505	0.024
cg26465289	chr16:72963399	ZFHX3	0.072	0.002
cg08709228	chr16:72965762	ZFHX3	0.986	0.020
cg15099137	chr16:72966213	ZFHX3	0.954	0.000
cg02605232	chr16:72967269	ZFHX3	0.231	0.005
cg27231624	chr16:72967626	ZFHX3	0.719	0.049
cg27623978	chr16:72968803	ZFHX3	0.764	0.038
cg15600244	chr16:72970175	ZFHX3	0.113	0.003
cg10246450	chr16:72970503	ZFHX3	0.352	0.012
cg05972185	chr16:72972644	ZFHX3	0.071	0.020
cg17088908	chr16:72972932	ZFHX3	0.867	0.076
cg26909217	chr16:72974588	ZFHX3	0.556	0.036
cg13744691	chr16:72976215	ZFHX3	0.125	0.006
cg04211829	chr16:72981203	ZFHX3	0.005	0.013
cg04052706	chr16:72981543	ZFHX3	0.684	0.008
cg26644885	chr16:72981599	ZFHX3	< 0.001	0.010
cg09858224	chr16:72981996	ZFHX3	0.719	0.002
cg04667640	chr16:72982116	ZFHX3	0.469	0.003
cg08692430	chr16:72982398	ZFHX3	0.450	0.010
cg10298059	chr16:72982446	ZFHX3	0.383	0.017
cg07051796	chr16:72982538	ZFHX3	0.014	0.001
cg01678913	chr16:72984472	ZFHX3	0.045	0.004
cg16651606	chr16:72984650	ZFHX3	0.832	0.002
cg05364570	chr16:72984764	ZFHX3	0.335	0.004
cg23661268	chr16:72987028	ZFHX3	0.241	0.035
cg06185532	chr16:72987360	ZFHX3	0.034	0.008
cg11136145	chr16:72988399	ZFHX3	0.033	0.034
cg13179077	chr16:72990563	ZFHX3	0.564	0.004
cg02736186	chr16:72990977	ZFHX3	0.269	0.013
cg05764102	chr16:72991344	ZFHX3	0.532	0.002
cg26573797	chr16:72991926	ZFHX3	0.414	0.000
cg08080174	chr16:72992580	ZFHX3	0.381	0.009
cg18527588	chr16:72993161	ZFHX3	0.877	0.003
cg05704496	chr16:72993237	ZFHX3	0.116	0.025
cg05366475	chr16:72993541	ZFHX3	0.019	0.001
cg14462566	chr16:72993790	ZFHX3	0.508	0.004
cg08813197	chr16:72993866	ZFHX3	0.199	0.003
cg06381966	chr16:72994598	ZFHX3	0.750	0.002
cg16563255	chr16:72995177	ZFHX3	0.667	0.007
cg14707682	chr16:73002013	ZFHX3	0.546	0.000
cg02969609	chr16:73003832	ZFHX3	0.296	0.002
cg20069798	chr16:73010721	ZFHX3	0.363	0.003

cg23274883	chr16:73010808	ZFHX3	0.768	0.010
cg23987077	chr16:73012207	ZFHX3	0.516	0.008
cg16057662	chr16:73017823	ZFHX3	0.461	0.010
cg02284258	chr16:73017982	ZFHX3	0.081	0.018
cg12703081	chr16:73018646	ZFHX3	0.540	0.007
cg19693972	chr16:73018672	ZFHX3	0.082	0.027
cg10298741	chr16:73019173	ZFHX3	0.060	0.024
cg22070783	chr16:73022010	ZFHX3	0.041	0.005
cg08094551	chr16:73023727	ZFHX3	0.452	0.005
cg17555914	chr16:73023997	ZFHX3	0.619	0.011
cg15938946	chr16:73024202	ZFHX3	0.836	0.049
cg08170679	chr16:73025828	ZFHX3	0.104	0.013
cg24805342	chr16:73029229	ZFHX3	0.828	0.005
cg06649004	chr16:73029234	ZFHX3	0.290	0.009
cg03901393	chr16:73029288	ZFHX3	0.889	0.051
cg10932166	chr16:73031218	ZFHX3	0.986	0.070
cg16829616	chr16:73035242	ZFHX3	0.549	0.009
cg03808233	chr16:73035287	ZFHX3	0.597	0.006
cg26091825	chr16:73044002	ZFHX3	0.188	0.002
cg13752020	chr16:73046138	ZFHX3	0.265	0.003
cg22460989	chr16:73049971	ZFHX3	0.696	0.001
cg16959721	chr16:73053290	ZFHX3	0.424	0.011
cg10415570	chr16:73053779	ZFHX3	0.479	0.004
cg05639334	chr16:73056524	ZFHX3	0.741	0.016
cg23130717	chr16:73057684	ZFHX3	0.597	0.011
cg09618520	chr16:73057948	ZFHX3	0.190	0.002
cg17492658	chr16:73058311	ZFHX3	0.037	0.003
cg03339247	chr16:73059888	ZFHX3	0.160	0.001
cg27262054	chr16:73067525	ZFHX3	0.528	0.004
cg02013904	chr16:73067772	ZFHX3	0.292	0.006
cg13121670	chr16:73069878	ZFHX3	0.313	0.008
cg06477050	chr16:73071300	ZFHX3	0.331	0.002
cg17877577	chr16:73071822	ZFHX3	0.001	0.002
cg09392505	chr16:73072333	ZFHX3	0.932	0.025
cg27364780	chr16:73074423	ZFHX3	0.027	0.001
cg03943249	chr16:73076266	ZFHX3	0.003	0.002
cg03805182	chr16:73078071	ZFHX3	0.116	0.011
cg10483525	chr16:73080909	ZFHX3	0.372	0.015
cg03878654	chr16:73081124	ZFHX3	0.086	0.011
cg03214730	chr16:73081558	ZFHX3	0.803	0.013
cg09306186	chr16:73081722	ZFHX3	0.281	0.005
cg03490839	chr16:73082051	ZFHX3	0.004	0.001
cg20631181	chr16:73082402	ZFHX3	0.299	0.002
cg05556210	chr16:73082405	ZFHX3	0.992	0.006
cg14912709	chr16:73082434	ZFHX3	0.011	0.000
cg08767708	chr16:73082503	ZFHX3	0.331	0.006

cg07427605	chr16:73082535	ZFHX3	0.828	0.005
cg04910662	chr16:73083014	ZFHX3	0.092	0.004
cg01743592	chr16:73083278	ZFHX3	0.154	0.002
cg07294323	chr16:73083327	ZFHX3	0.039	0.010
cg09754243	chr16:73083863	ZFHX3	0.025	0.001
cg05516390	chr16:73088677	ZFHX3	0.339	0.005
cg25152254	chr16:73090085	ZFHX3	0.790	0.047
cg10092685	chr16:73090591	ZFHX3	0.089	0.009
cg06086177	chr16:73090646	ZFHX3	0.080	0.003
cg04700292	chr16:73090723	ZFHX3	0.692	0.011
cg05915866	chr16:73090838	ZFHX3	0.071	0.037
cg07464477	chr16:73091565	ZFHX3	0.014	0.001
cg03202064	chr16:73091917	ZFHX3	< 0.001	0.001
cg07786668	chr16:73092391	ZFHX3	0.093	0.009
cg00614832	chr16:73092394	ZFHX3	0.012	0.013
cg21122147	chr16:73092500	ZFHX3	< 0.001	0.001
cg26474270	chr16:73092823	ZFHX3	0.167	0.003
cg11804517	chr16:73092997	ZFHX3	0.203	0.004
cg02310882	chr16:73093296	ZFHX3	0.465	0.011
cg07996255	chr16:73093309	ZFHX3	0.357	0.005
cg20213863	chr16:73093470	ZFHX3	0.461	0.014
cg13416486	chr16:73093652	ZFHX3	0.869	0.007

Notes: CpG ID corresponds to the unique CpG site identifier in the HumanMethylationEPIC array (Illumina). Chromosomal location denoted according human reference assembly GRCh37/hg19. All depicted P values are FDR adjusted P values. CpG-sites with an absolute mean methylation beta value difference > 0.15 and FDR adjusted P value < 0.05 were considered statistically significant.

*Included studies:

Buda P, Chyb M, Smorczewska-Kiljan A, et al. Association Between rs12037447, rs146732504, rs151078858, rs55723436, and rs6094136 Polymorphisms and Kawasaki Disease in the Population of Polish Children. *Front Pediatr* 2021; **9**: 624798.

Burgner D, Davila S, Breunis WB, et al. A genome-wide association study identifies novel and functionally related susceptibility Loci for Kawasaki disease. *PLoS Genet* 2009; **5**(1): e1000319.

Hoggart C, Shimizu C, Galassini R, et al. Identification of novel locus associated with coronary artery aneurysms and validation of loci for susceptibility to Kawasaki disease. *Eur J Hum Genet* 2021; **29**(12):1734–1744.

Johnson TA, Mashimo Y, Wu JY, et al. Association of an IGHV3-66 gene variant with Kawasaki disease. *J Hum Genet* 2021; **66**(5): 475–489.

Khor CC, Davila S, Breunis WB, et al. Genome-wide association study identifies FCGR2A as a susceptibility locus for Kawasaki disease. *Nat Genet* 2011; **43**(12): 1241–6.

Kim JJ, Hong YM, Sohn S, et al. A genome-wide association analysis reveals 1p31 and 2p13.3 as susceptibility loci for Kawasaki disease. *Hum Genet* 2011; **129**(5): 487–95.

Kim JJ, Park YM, Yoon D, et al. Identification of KCNN2 as a susceptibility locus for coronary artery aneurysms in Kawasaki disease using genome-wide association analysis. *J Hum Genet* 2013; **58**(8): 521–5.

Kuo HC, Li SC, Guo MM, et al. Genome-Wide Association Study Identifies Novel Susceptibility Genes Associated with Coronary Artery Aneurysm Formation in Kawasaki Disease. *PLoS One* 2016; **11**(5): e0154943.

Kwon YC, Kim JJ, Yu JJ, et al. Identification of the TIFAB Gene as a Susceptibility Locus for Coronary Artery Aneurysm in Patients with Kawasaki Disease. *Pediatr Cardiol* 2019; **40**(3): 483–488.

Kwon YC, Kim JJ, Yun SW, et al. Male-specific association of the FCGR2A His167Arg polymorphism with Kawasaki disease. *PLoS One* 2017; **12**(9): e0184248.

Lee YC, Kuo HC, Chang JS, et al. Two new susceptibility loci for Kawasaki disease identified through genome-wide association analysis. *Nat Genet* 2012; **44**(5): 522–5.

Onouchi Y, Ozaki K, Burns JC, et al. A genome-wide association study identifies three new risk loci for Kawasaki disease. *Nat Genet* 2012; **44**(5): 517–21.

Tsai FJ, Lee YC, Chang JS, et al. Identification of novel susceptibility Loci for kawasaki disease in a Han chinese population by a genome-wide association study. *PLoS One* 2011; **6**(2):e16853.

Supplementary Methods

Study protocol

Whole blood samples and clinical data from 43 MIS-C patients were retrospectively collected between April 16th, 2020 and August 17th, 2021 in seven Hospitals in Spain organized into collaborative groups. Whole blood samples were also obtained from 15 pediatric COVID-19 cases with no evidence of MIS-C, and from 69 healthy children and adolescents collected during the pre-COVID-19 era (before December 2019). Approvals were obtained by the corresponding Ethical Committees (PR052/21, Hospital Universitari de Bellvitge; PR(AMI)388/2016, Hospital Universitari Vall d'Hebron; PI_2020/35, Complejo Hospitalario de Navarra; CEIm 2314, Hospital Universitario de Burgos; PI-21-160, Hospital Universitari Germans Trias i Pujol). According to the Biomedical Research Law 14/2007, informed consents were signed to donate biological material for research purposes at the reference center. Clinical information has been collected, processed and stored under confidentiality policies, in accordance with the National Organic Law 3/2018, on the protection of personal data and guarantee of digital rights. Clinical data and biological samples arrived at our institution pseudonymized (de-identified) by the clinician or personnel authorized at the healthcare institution. Sensitive patient information showing the identity of the patient was only recorded at the healthcare institution. Biological samples were systematically collected and appropriately preserved for research studies. To this end, peripheral blood samples were drawn in EDTA Vacutainer® blood collection tubes and stored at - 80ºC until DNA extraction.

DNA methylation data

The DNA methylation status of the studied samples was obtained using the Infinium MethylationEPIC Array (~850,000 CpG sites), following the manufacturer's instructions for the automated processing of arrays with a liquid handler (Illumina Infinium HD Methylation Assay Experienced User Card, Automated Protocol 15019521 v01) (Moran et al., 2016). DNA methylation beta values were obtained from the raw IDAT files using the minfi package (v1.36.0) in R. Briefly, the pre-processing of the methylation data performed with the minfi package in R involved removal of erratic probe signals such as failed probes (probes with a detection value of $P > 0.01$), cross-reacting probes and probes that overlapped SNPs within ±1 base pair of CpG sites. Those probes that failed in more than 10% of samples were removed from the analysis, whereas the beta value of the probes that failed in less than 10% of samples was imputed using the median. XY chromosome probes were also removed. Finally, background correction and dye-based normalization were performed using ssNoob algorithm. The genomic analysis presented in the study was performed using the GRCh37 – hg19 human genome reference build, as described in the Illumina manifest file associated with the DNA methylation EPIC microarray.

Computational analyses

The MIS-C epigenetic signature, referred to hereafter as EPIMISC, was obtained by first identifying the probes differentially methylated between MIS-C cases and healthy control donors, filtering out in a second step those probes found to be differentially methylated between pediatric COVID-19 cases and healthy controls (**Figure S1**). This approach enabled us to effectively discover the differentially methylated probes between MIS-C and non-MIS-C cases. This involved deriving a linear model adjusted by the age covariate with the limma R package (v3.46.0) using the methylation values of the discovery dataset. A significance threshold for CpGs with a False Discovery Rate (FDR) adjusted P value < 0.05 and an absolute mean methylation beta difference between groups of > 0.15 was established. The significantly differential DNA methylation sites (**Table S3**) were used to train a supervised classification model based on a ridge-regularized logistic regression to predict MIS-C diagnosis using the glmnet R package (v4.1-1). Thus, the methylation beta values of the differential DNA methylation sites (**Table S3**) are the predictors of the ridge-regularized logistic regression and the outcome is the presence or absence of the EPIMISC signature. The classification model was optimized by tuning parameters (best performance with alpha = 0 from ridge regression, and regularization parameter lambda = 0.1) after resampling with 10-fold cross-validation carried out three times using the caret package in R (v6.0-86). To this end, we used the createFolds caret function to generate balanced cross-validation groupings from the discovery dataset in order to perform the 10-fold cross-validation. Once the model and tuning parameters values have been defined after resampling, our model performance was assessed using the receiver operating characteristic (ROC) and calibration curves. In the next step, the classification model was tested in the validation cohort and a corresponding confusion matrix derived.

Likewise, a supervised classification model was trained and optimized with the same parameters as previously described, but this time using the differentially methylated CpGs present in the Infinium Human Methylation 450K BeadChip to test it in external datasets from the Gene Expression Omnibus (GEO) for which only 450K data were available. The performance of the 450K model was also assessed using the ROC curve of the resamples. Finally, hierarchical clustering analysis was performed using the Ward.D clustering method with Manhattan distances in the gplots (v3.1.1) package in R. All analyses were performed within the R statistical environment (v4.0.3).

Gene Set Enrichment analysis

Gene set enrichment analysis was conducted using the Enrichr tool (Kuleshov et al., 2016) by performing hypergeometrical tests (one-tailed Fisher´s exact test) using Gene Ontology

Biological Process gene set. Enrichments were considered significant at Benjamini & Hochberg False Discovery Rate (FDR) adjusted $P < 0.05$.

Cell type deconvolution analysis

Cell type deconvolution analysis to calculate particular hematological cell populations based on blood-derived DNA methylation signatures (Salas et al., 2018) was performed using estimateCellCounts2 function from FlowSorted.Blood.EPIC (v1.8.0) package in R.

Statistical analyses

The entire cohort (N=127) was divided into a discovery (N=85) and validation cohorts (N=42) using the createDataPartition function from the caret R package (v6.0-86). We used this function to create one hundred (66% discovery and 33% validation) disease status and age-balanced splits of the entire dataset, so that the random sampling occurred within each disease status (MIS-C vs non-MIS-C) and age category (age groups were defined by 5 years intervals), preserving the overall class distribution of the data. We randomly selected one of the 100 balanced splits to divide samples into discovery and validation cohorts. This was done to avoid disparity in the frequencies of the observed classes (disease status and age) that can have a significant negative impact on model fitting.

We estimated the power of our EPIC array DNA methylation study to demonstrate the hypotheses according to an epigenome-wide association study (EWAS) power calculation technique previously described (Mansell et al., 2019). Thus, we determined that 97.4% of sites in the EPIC array (~850,000 sites) have power >90% to detect a 10% mean methylation difference (effect size) with N=85 samples (our discovery cohort sample size) at a significance threshold of $P<0.0001$.

Principal component analysis (PCA) was performed using PCAtools R package (v2.2.0) as an unsupervised method for data exploration in order to detect the greatest sources of variation in our dataset. We explored all the clinical variables available (**Table 1**) and found that disease status and age were the two main sources of variation in our dataset. Thus, we adjusted our analysis by age to correct its confounding effect.

To assess whether the assumptions of linear regression are satisfied, we used the gvlma R package (v1.0.0.3) considering a significance threshold ($P < 9.42 \times 10^{-8}$) previously described for the EPIC array (Mansell et al., 2019). As a result, 99.99% of sites in the EPIC array fulfill the linearity assumption (linear relationship between independent variables (both disease status and age) and dependent variable (CpG site methylation beta value)), 98.9% of sites

show homoscedasticity (constant variance of the residuals), 90.63% of sites present a symmetrical normal distribution of the residuals and 86.21% present a bell-shaped normal distribution of the residuals. Finally, a global test was performed providing an omnibus test of the four individual statistical tests, showing that 83.24% of sites in the EPIC array fulfill all linearity assumptions.

Furthermore, the differential methylation analysis between MIS-C and non-MIS-C cases was applied to beta values employing an empirical Bayesian framework linear model from the limma R package (v3.46.0), a model suitable for DNA methylation data (Mansell et al., 2019). limma operates on a matrix of methylation values, where each row is a probe (EPIC array site) and each column corresponds to a sample, and it fits a linear model to each row of data (Ritchie et al., 2015). For each probe, we have a vector of DNA methylation values and a design matrix that relates these values to some coefficients of interest (disease status and age). Thus, the dependent variable in the linear model is the methylation value and the independent variables are disease status and age.

References

Kuleshov MV, Jones MR, Rouillard AD, et al. Enrichr: a comprehensive gene set enrichment analysis web server 2016 update. *Nucleic Acids Research* 2016; **44** (W1): W90–7.

Mansell G, Gorrie-Stone TJ, Bao Y, et al. Guidance for DNA methylation studies: statistical insights from the Illumina EPIC array. *BMC Genomics* 2019; **20**: 366.

Moran S, Arribas C, Esteller M. Validation of a DNA methylation microarray for 850,000 CpG sites of the human genome enriched in enhancer sequences. *Epigenomics* 2016 Mar;**8**(3): 389–99.

Ritchie ME, Phipson B, Wu D, Hu Y, Law CW, Shi W, et al. limma powers differential expression analyses for RNA-sequencing and microarray studies. *Nucleic Acids Res* 2015 Apr 20; **43**(7): e47.

Salas LA, Koestler DC, Butler RA, Hansen HM, Wiencke JK, Kelsey KT, Christensen BC. An optimized library for reference-based deconvolution of whole-blood biospecimens assayed using the Illumina HumanMethylationEPIC BeadArray. *Genome Biol* 2018; **19**: 64.

References of genetic loci associated to COVID-19 included in the study

1. Bastard P, Rosen LB, Zhang Q, et al. Autoantibodies against type I IFNs in patients with life-threatening COVID-19. *Science* 2020; **370**: eabd4585.
2. Mousa M, Vurivi H, Kannout H, et al. Genome-wide association study of hospitalized COVID-19 patients in the United Arab Emirates. *EBioMedicine* 2021; **74**: 103695.
3. Pairo-Castineira E, Clohisey S, Klaric L, et al. Genetic mechanisms of critical illness in Covid-19. *Nature* 2020; **591**: 92–98.
4. Severe Covid-19 GWAS Group, Ellinghaus D, Degenhardt F, et al. Genomewide association study of severe Covid-19 with respiratory failure. *N Engl J* Med 2020; **383**: 1522–34.
5. Singh H, Choudhari R, Nema V, Khan AA. ACE2 and TMPRSS2 polymorphisms in various diseases with special reference to its impact on COVID-19 disease. *Microb Pathog* 2020; **150**: 104621.

6. van der Made CI, Simons A, Schuurs-Hoeijmakers J, et al. Presence of genetic variants among young men with severe COVID-19. *JAMA* 2020; **324**: 1–11.
7. Zhang Q, Bastard P, Liu Z, et al. Inborn errors of type I IFN immunity in patients with life-threatening COVID-19. *Science* 2020; **370**: eabd4570.

References of genetic loci associated to MIS-C included in the study

1. Chou J, Platt CD, Habiballah S, et al. Mechanisms underlying genetic susceptibility to multisystem inflammatory syndrome in children (MIS-C). *J Allergy Clin Immunol* 2021; **148**(3): 732–738.e1.
2. Lee PY, Platt CD, Weeks S, et al. Immune dysregulation and multisystem inflammatory syndrome in children (MIS-C) in individuals with haploinsufficiency of SOCS1. *J Allergy Clin Immunol* 2020; **146**(5): 1194–1200.e1.

References of genetic loci associated to Kawasaki disease included in the study

1. Buda P, Chyb M, Smorczewska-Kiljan A, et al. Association Between rs12037447, rs146732504, rs151078858, rs55723436, and rs6094136 Polymorphisms and Kawasaki Disease in the Population of Polish Children. *Front Pediatr* 2021; **9**: 624798.
2. Burgner D, Davila S, Breunis WB, et al. A genome-wide association study identifies novel and functionally related susceptibility Loci for Kawasaki disease. *PLoS Genet* 2009; **5**(1): e1000319.
3. Hoggart C, Shimizu C, Galassini R, et al. Identification of novel locus associated with coronary artery aneurysms and validation of loci for susceptibility to Kawasaki disease. *Eur J Hum Genet* 2021; **29**(12): 1734-1744.
4. Johnson TA, Mashimo Y, Wu JY, et al. Association of an IGHV3-66 gene variant with Kawasaki disease. *J Hum Genet* 2021; **66**(5): 475-489.
5. Khor CC, Davila S, Breunis WB, et al. Genome-wide association study identifies FCGR2A as a susceptibility locus for Kawasaki disease. *Nat Genet* 2011; **43**(12): 1241-6.
6. Kim JJ, Hong YM, Sohn S, et al. A genome-wide association analysis reveals 1p31 and 2p13.3 as susceptibility loci for Kawasaki disease. *Hum Genet* 2011; **129**(5): 487-95.
7. Kim JJ, Park YM, Yoon D, et al. Identification of KCNN2 as a susceptibility locus for coronary artery aneurysms in Kawasaki disease using genome-wide association analysis. *J Hum Genet*. 2013; **58**(8): 521-5.
8. Kuo HC, Li SC, Guo MM, et al. Genome-Wide Association Study Identifies Novel Susceptibility Genes Associated with Coronary Artery Aneurysm Formation in Kawasaki Disease. *PLoS One* 2016; **11**(5): e0154943.
9. Kwon YC, Kim JJ, Yu JJ, et al. Identification of the TIFAB Gene as a Susceptibility Locus for Coronary Artery Aneurysm in Patients with Kawasaki Disease. *Pediatr Cardiol* 2019; **40**(3): 483-488.
10. Kwon YC, Kim JJ, Yun SW, et al. Male-specific association of the FCGR2A His167Arg polymorphism with Kawasaki disease. *PLoS One* 2017; **12**(9): e0184248.
11. Lee YC, Kuo HC, Chang JS, et al. Two new susceptibility loci for Kawasaki disease identified through genome-wide association analysis. *Nat Genet* 2012; **44**(5): 522-5.
12. Onouchi Y, Ozaki K, Burns JC, et al. A genome-wide association study identifies three new risk loci for Kawasaki disease. *Nat Genet* 2012; **44**(5): 517-21.
13. Tsai FJ, Lee YC, Chang JS, et al. Identification of novel susceptibility Loci for kawasaki disease in a Han chinese population by a genome-wide association study. *PLoS One* 2011; **6**(2): e16853.
